# Air- and Water-Stable Heteroleptic Copper (I) Complexes Bearing Bis(indazol-1-yl)methane Ligands: Synthesis, Characterisation, and Computational Studies

**DOI:** 10.3390/molecules29010047

**Published:** 2023-12-20

**Authors:** David Moreno-da Costa, César Zúñiga-Loyola, Federico Droghetti, Stephania Robles, Alondra Villegas-Menares, Nery Villegas-Escobar, Ivan Gonzalez-Pavez, Elies Molins, Mirco Natali, Alan R. Cabrera

**Affiliations:** 1 Departamento de Química Inorgánica, Facultad de Química y de Farmacia, Pontificia Universidad Católica de Chile, Avenida Vicuña Mackenna 4860, Macul, Santiago 7820436, Chile; aevillegas@uc.cl; 2 Departamento de Química de Los Materiales, Facultad de Química y Biología, Universidad de Santiago de Chile, Casilla 40, Correo 33, Sucursal Matucana, Santiago 9170022, Chile; cesar.zunigal@usach.cl (C.Z.-L.); stephania.robles@usach.cl (S.R.); 3 Department of Chemical, Pharmaceutical and Agricultural Sciences, University of Ferrara, via L. Borsari 46, 44121 Ferrara, Italy; federico.droghetti@unife.it; 4 Departamento de Físico-Química, Facultad de Ciencias Químicas, Universidad de Concepción, Edmundo Larenas 129, Concepción 4070371, Chile; neryvillegas@udec.cl; 5 Departamento de Química, Facultad de Ciencias Naturales, Matemática y del Medio Ambiente, Universidad Tecnológica Metropolitana, Las Palmeras 3360, Ñuñoa, Santiago 7800003, Chile; igonzalezp@utem.cl; 6 Institut de Ciència de Materials de Barcelona, Consejo Superior de Investigaciones Científicas, Campus de la UAB, 08193 Barcelona, Spain; elies.molins@icmab.es

**Keywords:** bis(indazol-1-yl)methane, Cu(I), heteroleptic complex, time-dependent DFT

## Abstract

A series of four novel heteroleptic Cu(I) complexes, bearing bis(1*H*-indazol-1-yl)methane analogues as *N*,*N* ligands and DPEPhos as the *P,P* ligand, were synthesised in high yields under mild conditions and characterised by spectroscopic and spectrometric techniques. In addition, the position of the carboxymethyl substituent in the complexes and its effect on the electrochemical and photophysical behaviour was evaluated. As expected, the homoleptic copper (I) complexes with the *N*,*N* ligands showed air instability. In contrast, the obtained heteroleptic complexes were air- and water-stable in solid and solution. All complexes displayed green-yellow luminescence in CH_2_Cl_2_ at room temperature due to ligand-centred (LC) phosphorescence in the case of the Cu(I) complex with an unsubstituted *N,N* ligand and metal-to-ligand charge transfer (MLCT) phosphorescence for the carboxymethyl-substituted complexes. Interestingly, proper substitution of the bis(1*H*-indazol-1-yl)methane ligand enabled the achievement of a remarkable luminescent yield (2.5%) in solution, showcasing the great potential of this novel class of copper(I) complexes for potential applications in luminescent devices and/or photocatalysis.

## 1. Introduction

Photoactive and stable complexes based on transition metals have been studied for their applications in advanced technology and synthetic methodology [1,2,3,4,5,6,7,8]. In this context, ruthenium and iridium complexes are two of the most efficient species for photoactive applications because they (i) are relatively easy to prepare, (ii) have reversible electrochemical behaviour, (iii) absorb light in the visible spectral region (VIS), (iv) have electronically durable excited states, (v) exhibit intense luminescence, and (vi) have high redox potentials in the excited state [9,10]. It is worth pointing out that an essential drawback of these metals is their relatively elevated cost and toxicity [11,12]. However, Earth-abundant photoactive compounds have emerged as an alternative solution, enhancing research on this topic in recent years [3,4,13,14].

In this regard, copper complexes have played a significant role in reducing the toxicity and the costs of obtaining photoactive materials [15]. Two of the most studied fields for copper complexes are electrical/light energy conversion (e.g., active materials in LEEC devices) [16] and synthetic methodologies such as photoredox catalysts [17,18,19]. Two challenges are identifiable in obtaining stable and photoactive Cu(I) complexes. The first is the capacity of the ligands to ensure an electronic environment to avoid the oxidation of the metal centre by changing the geometry from a distorted tetrahedral shape to distorted square planar. The second challenge is tuning the optoelectronic properties of the copper(I) complexes for the desired purposes. These challenges converge in the engineering and appropriate selection of the ligands.

Bis(1*H*-indazol-1-yl)methane and bis(2*H*-indazol-2-yl)methane have been used as *N,N* ligands in several metal complexes due to their two *N*-donor bidentate capacity [20,21]. Trofimenko et al. reported the use of poly(pyrazol-1-yl)alkane ligands in the 1960s. Using these ligands, a six-membered ring is formed when a bis(indazolyl)alkane coordinates to the metal centre in a bidentate coordination. Importantly, bis(indazolyl)alkane ligands offer the possibility of modulating the steric hindrance and the electronic properties by incorporating substituents in the heterocycle rings and at the central methylene fragment [22,23,24]. Furthermore, bridging spacers can be introduced in the bis(pyrazol-1-yl) moieties to generate flexible di- and polytopic ligands with the capacity to afford complexes with different nuclearity based on several *d*-block metals, showing unusual magnetic properties [25,26,27]. Regarding bis(indazol-1-yl)methane ligands, Ballesteros et al. reported a mild synthesis of these compounds in 1985 using the nucleophilic addition to aldehydes producing 1-(hydroxymethyl)-1*H*-indazole followed by the nucleophilic substitution of the hydroxyl group by another indazole equivalent [28].

The scope of using bis(indazol-1-yl)methane ligands is well known in coordinative complexes. For instance, López et al. reported bis(indazolyl)methane ligands substituted in the methylene scaffold by one pyridine unit and used in the synthesis of a rhodium tripodal complex [29,30]. On the other hand, Tabassum et al. prepared homobinuclear iron(III) and copper(II) complexes bearing bis(indazolyl)methanes as ligands, which exhibited oxygen carrier properties [31].

Focusing on obtaining easily accessible Cu(I) complexes and studying their photophysical properties, herein we report the synthesis and characterisation of a series of heteroleptic Cu(I) complexes bearing bis(indazol-1-yl)methane analogues as *N,N* ligands and bis[(2-diphenylphosphino)phenyl] ether (DPEPhos) as the *P,P* auxiliary ligand. In the present work, different *N,N* ligand substitution patterns were evaluated with the carboxymethyl moiety as the electron-withdrawing group. The purpose of the present study is to characterise and give insights into the effect of the substituent’s position in the copper(I) complexes of the type [Cu(*N,N*)(*P,P*)]^+^ in terms of electrochemical and photophysical behaviour.

## 2. Results and Discussion

### 2.1. Synthesis and Characterisation of Compounds

#### 2.1.1. Synthesis and Characterisation of Bis(indazol-1-yl)methane Analogues Ligands

**Scheme 1:**
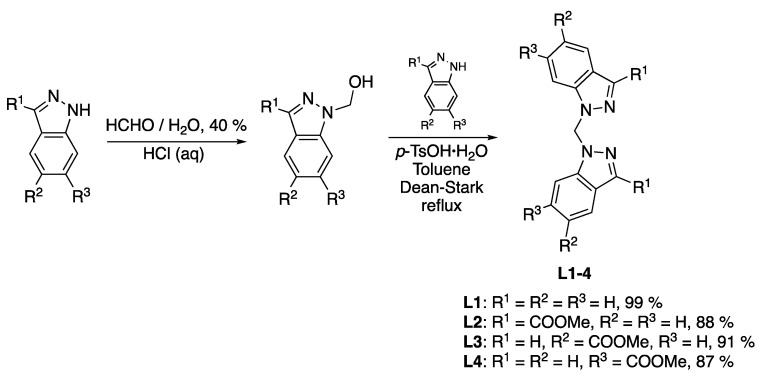
Synthetic route to obtaining bis(1*H*-indazol-1-yl)methane ligands **L1-4**.

The bis(indazol-1-yl)methane analogues **L1-4** were synthesised according to the procedure of Ballesteros et al. [28]. The first step corresponds to the nucleophilic addition of the respective indazole into formaldehyde in acidic media, resulting in an indazolilmethanol moiety. After the isolation, the indazolilmethanol is submitted to react with a second equivalent of the initial indazole analogue in Dean–Stark conditions with toluene to produce the desired bis(indazol-1-yl)methanes **L1-4** (Scheme 1).

The bis(1*H*-indazol-1-yl)methane ligands were identified for specific moieties in the ^1^H and ^13^C NMR analysis. The methyl group in the ester moiety was found at 3.19 ppm for **L2**, 3.89 for **L3**, and 3.98 ppm for **L4**, revealing a low field displacement of the chemical shift by the change in the group’s position. On the other hand, the carbon atom in the methyl group was found at 52.4 ppm in **L2**, 52.6 ppm in **L3**, and 52.5 ppm in **L4**. The protons of the methylene bridge are in the range of 60.0–63.0 ppm, with specific chemical shifts at 63.6 ppm (**L2**), 62.0 ppm (**L3**), and 61.4 ppm (**L4**). The *ESI* describes detailed NMR assignments and other characterisation techniques. For **L3**, the molecular structure was confirmed by single-crystal X-ray diffraction (XRD), as shown in Figure S9.

#### 2.1.2. Synthesis and Characterisation of Heteroleptic Cu(I) Complexes with Bis(indazol-1-yl)methane Ligands

**Scheme 2:**
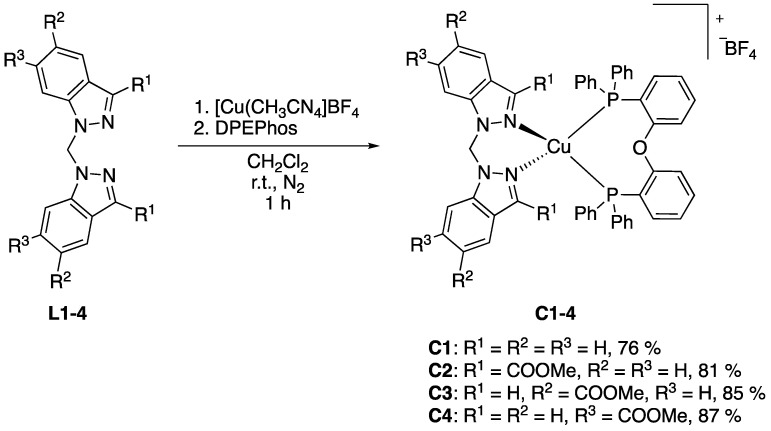
Synthetic route to obtaining Cu(I) complexes **C1-4**.

The synthesis of the desired complexes required a stoichiometric control of each reagent due to the complexation reaction by ligand exchange. The first step was the exchange of two acetonitrile moieties in [Cu(CH_3_CN)_4_]BF_4_ with one unit of the analogues **L1-4** in a dry CH_2_Cl_2_/CH_3_CN mixture with constant stirring for 30 min under a nitrogen atmosphere. The second step was the addition of DPEPhos, where the bidentate biphosphine removed the acetonitrile moieties to produce the heteroleptic Cu(I) complexes **C1-4** (Scheme 2), obtained in good isolated yields (76–87%).

All the complexes were analysed by ^1^H and ^13^C NMR in one- and two-dimensional experiments together with ^19^F, ^11^B, and ^31^P NMRs. In general, the **C2**-**4** aromatic hydrogens in the coordinated *N,N* ligands were in the high field zone between 8.84 and 8.03 ppm; the aromatic hydrogens in the *P,P* ligand were displaced to the lower field, between 7.30 and 6.89 ppm. On the contrary, **C1** did not present this order of chemical shift in ^1^H NMR between the aromatic hydrogens of both ligands coordinated to the metal centre. The methylene bridge appeared at 7.07/57.9 ppm (^1^H/^13^C NMR) for **C1**, 6.88–6.85/63.1 ppm for **C2**, 7.09/58.4 ppm for **C3**, and 7.08/58.5 ppm for **C4**. On the other hand, the methyl group in the ester moiety for **C2-4** was observed at 3.90/52.5 ppm, 4.03/52.8 ppm, and 4.02/52.8 ppm, respectively. Additionally, FT-IR and HRMS analyses were performed, which confirmed the obtaining of the desired complexes. The complete characterisation of **C1-4** is described in the *ESI*.

**Figure 1:**
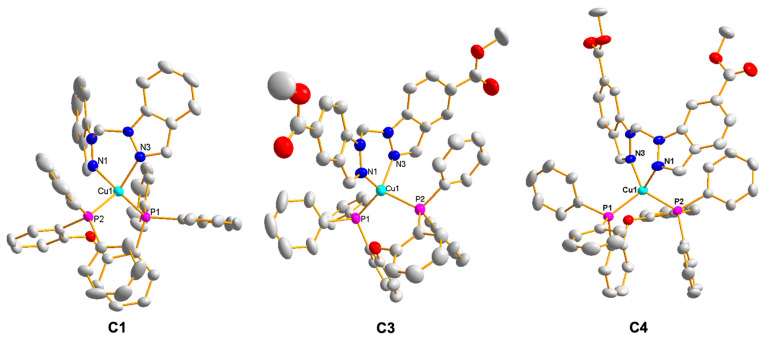
Molecular structures of **C1**, **C3**, and **C4** obtained by XRD. Hydrogen atoms, solvent molecules, and counterion were omitted for clarity. Thermal ellipsoids are at 30% probability.

In addition, X-ray diffraction analysis was performed on single crystals of **C1**, **C3**, and **C4**, obtaining their molecular structures as shown in Figure 1. The suitable single crystals for measuring were obtained by slow evaporation of the dichloromethane solution of the compounds into toluene. All complexes exhibited pseudo-tetrahedral geometry and a heteroleptic mononuclear coordination centre, with τ_4_ values of 0.86 [32]. For further details on bond distances, angles, and torsion, see the *ESI*.

### 2.2. Cyclic Voltammetry

The electrochemical characterisation of each complex was performed using cyclic voltammetry (CV) at room temperature in N_2_-saturated CH_2_Cl_2_ solution using 0.1 mol L^–1^ TBAPF_6_ as the supporting electrolyte (Figures S49–S52 of the *ESI*). All the relevant redox potentials are summarised in Table 1. Upon anodic scan, all **C1**-**4** complexes exhibited irreversible waves, which can be assigned to Cu(I)/(II) oxidation processes in agreement with previous works [33,34,35,36,37]. The potentials extracted were weakly dependent on the nature of the *N*,*N* ligand, suggesting no relevant electronic effects of the substituents on the oxidation process. Upon cathodic scan, irreversible processes were observed, which can be assigned to the reduction of the *N*,*N* ligand (L/L^−^) [37,38]. In this case, remarkable differences were recorded depending on the nature of the metal complex (Table 1). A more negative reduction potential was indeed measured for complex **C1**. At the same time, less cathodic values were registered for the remaining complexes **C2-4**, reflecting the presence of the electron-withdrawing methoxycarbonyl group in the *N*,*N* ligand. Interestingly, a less negative reduction potential was recorded for complex **C4** when compared to the same process in the **C3** isomer, suggesting that the position of the substituent also has an important role in the energetics of the metal complexes.

**Figure 2:**
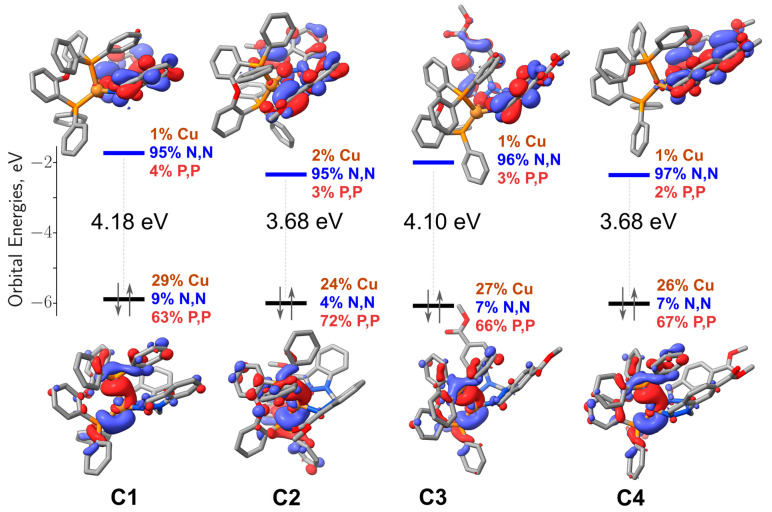
Frontier molecular orbitals iso-surfaces (0.03 a.u.) for **C1**-**4**, orbital contributions for the metal centre (Cu), and *N,N* and *P,P* ligands are also shown. The energy gap is reported in eV.

To obtain deeper knowledge of the electronic structure of complexes **C1**-**4**, standard and time-dependent DFT computations were performed for the B3LYP/6-31G(d,p)-LANL2DZ(Cu) using CH_2_Cl_2_ as solvent (see the computational section for further details). Frontier molecular orbital iso-surfaces, atomic orbital contributions, and HOMO–LUMO energy gaps (Δ_HL_) are displayed for all compounds in Figure 2. A full orbital composition analysis for relevant orbitals is given in Table S9 in the *ESI*. As can be seen from Figure 2, for all complexes, the HOMO is predominantly composed of the *P,P* ligand (~67% contribution on average) and the *d*-orbitals of Cu(I) (~26%). Conversely, a minor contribution (below 10%) to the HOMO was observed from the *N,N* ligand. The HOMO energies are −5.95 eV (**C1**), −6.06 eV (**C2**), −6.13 eV (**C3**), and −6.07 eV (**C4**). It is worth noting that the HOMO energy in complexes **C2-4** is roughly independent of the position of the methoxycarbonyl groups in the *N,N* ligand, in good agreement with the electrochemical data. On the other hand, the LUMO iso-surfaces were predominantly located on the *N,N* ligand, with over 95% contribution compared to the *P,P* ligand (~3%) and the metal centre (~1%) (see Figure 2). Contrarily to the HOMO energies, the LUMO energies were found to be modulated by the position of the −COOCH_3_ group and therefore represent the major source of the variation in the observed energy gaps, as expected according to the electrochemical experiments. The obtained LUMO energies were −1.77 eV (**C1**), −2.38 eV (**C2**), −2.03 eV (**C3**), and −2.40 eV (**C4**). In general, the methoxycarbonyl group decreases the HOMO-LUMO gap for **C2**, **C4**, and **C3** compared to the parent complex **C1**. The electron-withdrawing nature of the −COOCH_3_ group triggers the stabilisation of the LUMO energy.

### 2.3. UV-Visible Absorption Studies

**Figure 3:**
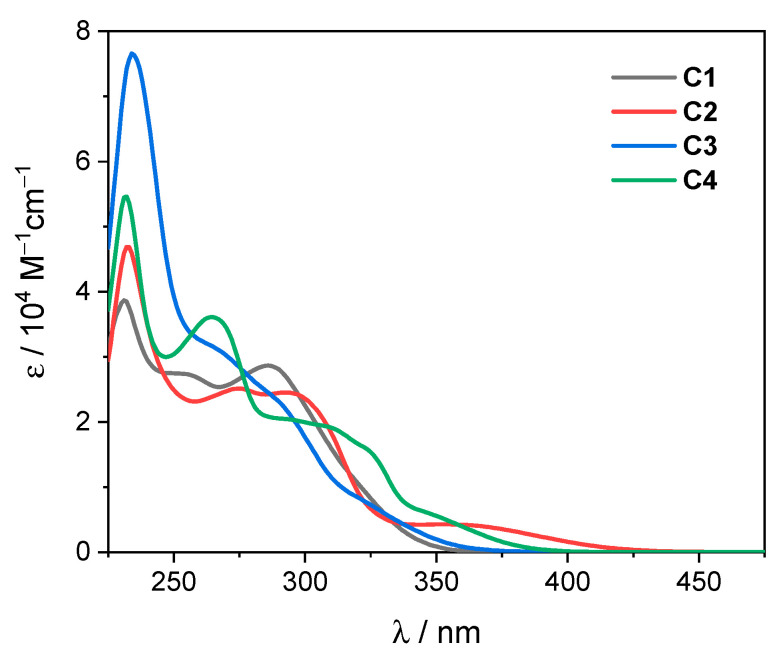
Absorption spectra of **C1-4** in CH_2_Cl_2_.

The **C1-4** complexes were characterised by UV-Vis spectrophotometry in dichloromethane as the solvent. The obtained UV-Vis spectra are shown in Figure 3. Complex **C1** displays the more blue-shifted absorptions, in agreement with the largest HOMO-LUMO energy gap predicted by electrochemistry and DFT computations (see above). Three bands can be observed with relative maxima at 230, 252, and 290 nm, associated with spin-allowed ligand-centred (^1^LC) transitions involving both the *N,N* and the *P,P* ligands (attenuation coefficients of ε > 20,000 M^−1^cm^−1^). At the same time, a tail at longer wavelengths can be ascribed to spin-allowed metal-to-ligand charge transfer transitions (^1^MLCT). Complex **C2**, on the other hand, displays similar spectral patterns below 300 nm due to LC transitions and a red-shifted MLCT band centred at 357 nm (attenuation coefficients of ε = 4200 M^−1^cm^−1^), consistent with a lower HOMO-LUMO energy gap imparted by the presence of the methoxycarbonyl group. Finally, complexes **C3** and **C4** exhibit spin-allowed LC transitions below 310 and 340 nm, respectively, with a pronounced shoulder at longer wavelengths assigned to spin-allowed MLCT absorptions.

**Figure 4:**
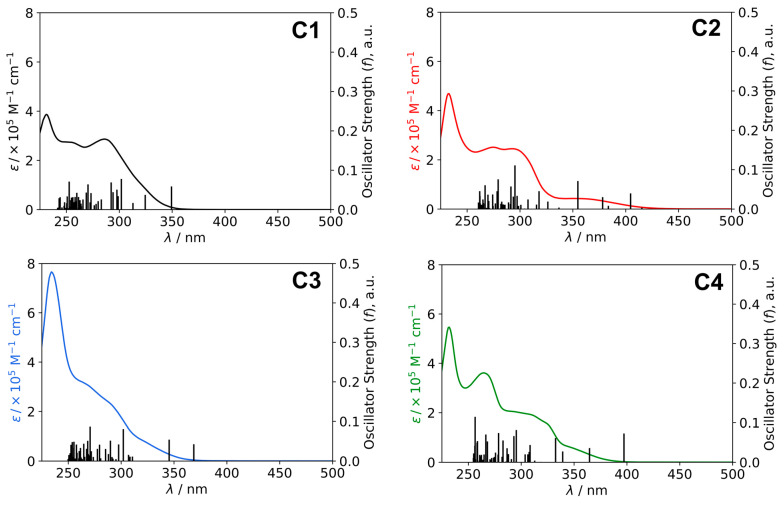
Normalised absorption spectra of complexes **C1-4** in CH_2_Cl_2_. Vertical electronic transitions and oscillator strengths (*f*, in a.u.) computed at the B3LYP-D3/6-31G(d,p)-LANL2DZ(Cu) are also shown.

Time-dependent DFT computations were performed to characterise the electronic transitions that gave rise to the experimental absorption spectra. Characterisation of relevant excited states for complexes **C1**-**4** is reported in Table 2. Figure 4 overlaps the experimental absorption spectra with computed TD-DFT vertical absorptions for **C1**-**4**. From Table 3, it can be seen for all systems that the S_1_ state arises predominantly from an HOMO→LUMO electronic transition. For **C2**, an extra HOMO −1→LUMO electronic transition also contributes to the lowest energy excited state. All complexes showed an S_1_ excited state composed of mixed ^1^MLCT and ^1^LLCT electronic transitions. In particular, the ^1^MLCT transition involves electron shift from the *d*-electrons of the metal centre to the π* orbital on the *N,N* ligand. At the same time, the ^1^LLCT is composed of a transition from the *P,P* ligand to the *N,N* ligand (π*) (see orbital composition analysis in the *ESI*). For the parent system **C1**, no transitions were observed above 350 nm (Figure 4). Quantum chemistry computations corroborate these experimental findings with the lowest energy excitation at 349 nm arising from a HOMO-LUMO transition (*f* = 0.059 au) of mixed ^1^MLCT and ^1^LLCT characters. As expected, including the methoxycarbonyl groups in complexes **C2**-**4** helps activate red-shifted transitions. Notably, the three isomers differ in the number of allowed electronic transitions near the visible region. For **C2**, four electronic transitions were found to be responsible for the band observed above 350 nm in the absorption spectrum (see Figure 4). Detailed information about crucial excited states is provided in Table 3. The results show that S_1_ and S_2_ states are formed by excitations from the HOMO –1 and HOMO to the LUMO, while for S_3_ and S_4_, electrons from the same occupied orbitals populate the LUMO + 1. For **C3** and **C4**, the formation of S_1_ and S_2_ states are given by HOMO-to-LUMO and HOMO-to-LUMO + 1 electronic transitions, respectively.

**Figure 5:**
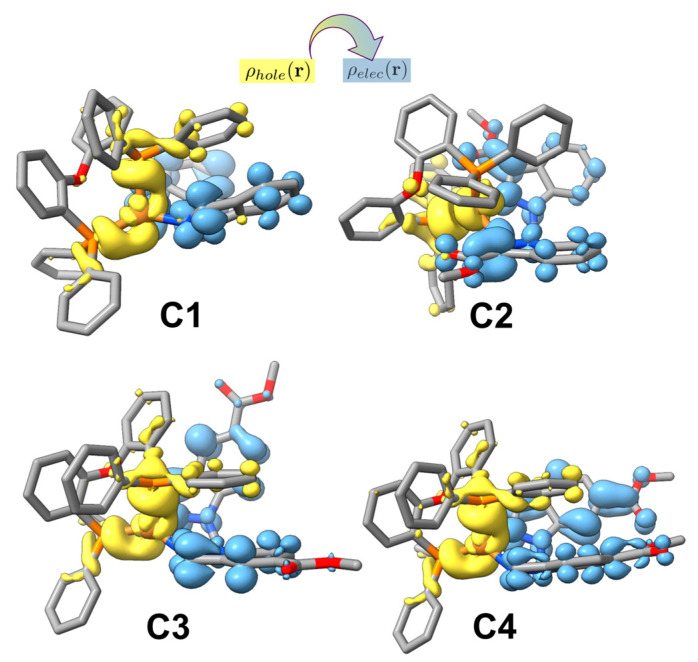
Hole–electron distributions, in yellow and blue, respectively, of the first excited state for systems **C1**-**4** (isovalue 0.0015 a.u.).

As two predominant electronic transitions generate the first excited state of **C2**, compared to the regular HOMO→LUMO transition exhibited in the remaining complexes, hole–electron distributions were computed for a fair comparison. Computed hole (yellow)–electron (blue) distributions are displayed in Figure 5. From the hole–electron distributions, the charge transfer nature of the S_1_ state is evident. More precisely, the electronic excitation from the metal centre and the *P,P* ligand (hole) to the *N,N* ligand (electron) in a mixed-MLCT/LLCT transition near 350 nm can be verified. On the other hand, from the hole–electron distributions, it can be observed that the methoxycarbonyl groups in **C3** and **C4** help to delocalise the electron density, enhancing the charge transfer character of the resulting bands. For **C4**, the slightly improved delocalisation of the electron distribution compared to **C3** coincides with a more allowed electronic transition (*f* = 0.073) due to the different positions of the methoxycarbonyl group. Contrarily, in **C2**, the same group condenses the electron distribution closer to the metal centre. This results in a lower charge transfer character and a much less intense band (*f* = 0.004), in agreement with the lower attenuation coefficients experimentally measured (Figure 3). Hole–electron distributions for other excited states of **C2** are provided in the *ESI*.

### 2.4. Emission Studies

**Figure 6:**
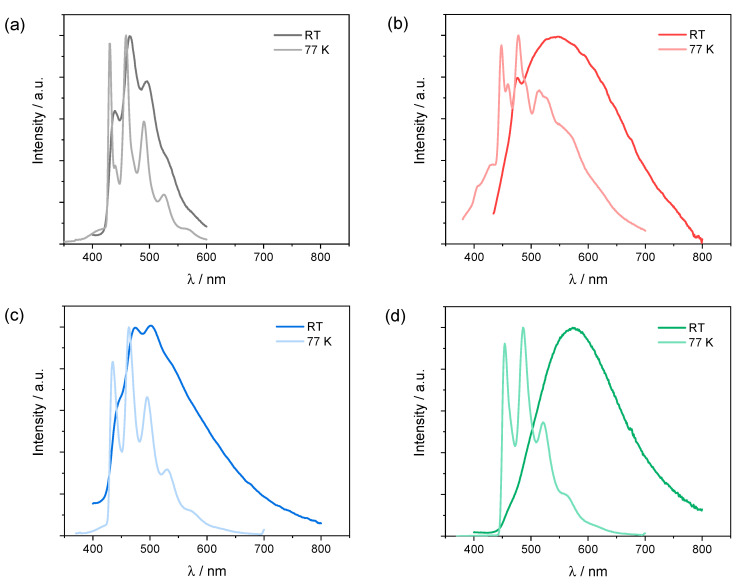
Luminescence spectra of complexes **C1-4** in N_2_-purged CH_2_Cl_2_ and in a rigid matrix (2-MeTHF) at 77 K: (**a**) **C1** (excitation at 320 nm at RT, 330 nm at 77 K), (**b**) **C2** (excitation at 410 nm at RT, 360 nm at 77 K), (**c**) **C3** (excitation at 355 nm at RT, 350 nm at 77 K), and (**d**) **C4** (excitation at 355 nm at RT, 350 nm at 77 K).

The luminescence properties of complexes **C1-4** were studied in degassed CH_2_Cl_2_ solution and in a 2-MeTHF glassy matrix at 77 K by means of both steady-state and time-resolved emission techniques. Figure 6 depicts the luminescence spectra of the complexes, while all the relevant luminescence data are collected in Table 3.

Complex **C1** displays a structured emission in CH_2_Cl_2_ solution at room temperature (Figure 6a), independent of the excitation wavelength, with three maxima at 440, 466, and 495 nm and a lifetime of 4.2 μs (Figure S53). This luminescence is similar to that recorded in the rigid matrix at 77 K regarding spectral shape and energy, and is compatible with the spectral patterns expected for the phosphorescence of indazole [39]. This evidence indicates that the observed emission in **C1** is assignable to phosphorescence from a triplet excited state of LC nature involving the *N*,*N* ligand.

On the other hand, in the case of complexes **C2-4**, the emission spectra at room temperature are generally broad and can be preferentially assigned to phosphorescence from a triplet excited state of MLCT character. Instead, a structured emission is observed at 77 K, akin to complex **C1**, whose spectral characteristics point to a triplet LC involving the *N*,*N* ligand [39]. The change in the emitting excited state at room temperature compared to the prototype complex **C1** can be associated with the presence of the methyl–ester functional groups. These have indeed been observed to shift the reduction potential of the *N*,*N* ligand to less negative values, thus decreasing the energy of the triplet MLCT excited state in **C2-4** in contrast to **C1**. The change in the emission nature between room temperature and 77 K conditions can be related to the presence of a lowest-lying triplet MLCT state close to the LC state involving the *N*,*N* ligand and the increase in energy of the charge transfer state at 77 K due to the rigid matrix conditions. Consistent with this hypothesis, for all complexes **C2-4** in CH_2_Cl_2,_ a bi-exponential decay was detected using time-resolved emission spectroscopy (Figures S54–S56 and Table 3), with the two lifetimes likely reflecting the kinetics of mutual population and ground state decay of both triplet MLCT and LC excited states [40,41]. Interestingly, in the case of complex **C3**, the proportion of the two kinetic components strongly depends on the analysis wavelength (Figure S55), suggesting that the emission band can be the result of a superposition of luminescence features from the two different excited states (see below).

The interplay between MLCT and LC excited states is observed to impact the efficiencies of the luminescence process in solution. As a matter of fact, low emission quantum yields were measured for complexes **C2** and **C3** (see Table 3). The net decrease in emission yield observed in the case of **C2** can also be determined by the non-optimal bite angle of the *N*,*N* ligand induced by the ester substituents, resulting in enhanced non-radiative deactivation routes involving the triplet MLCT excited state [42]. On the other hand, a larger emission yield was observed for complex **C4** (viz. 2.5%), consistent with the (i) lower energy of the emission band and the lower redox gap (ΔE_ox-red_, Table 1) in the **C2-4** series, (ii) the correspondingly lower energy of the MLCT excited state, and (iii) the resulting lower interaction with the local triplet LC state of the *N*,*N* ligand.

**Figure 7:**
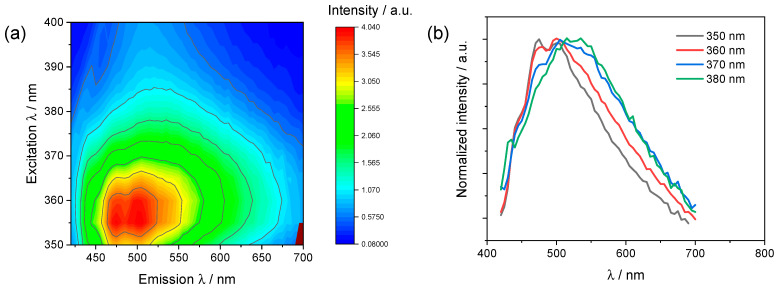
(**a**) Emission map of complex **C3** in CH_2_Cl_2_ and (**b**) relevant emission spectra.

Interestingly, a further confirmation of the interplay between triplet MLCT and LC excited state comes from the detailed analysis of the emission properties of complex **C3** in CH_2_Cl_2_. While the emission spectra of both complexes **C2** and **C4** are independent of the excitation wavelength, subtle differences were observed in the case of complex **C3**. Figure 7 reports the emission map of complex **C3** in CH_2_Cl_2_ along with the relevant emission spectra. Inspection of the data shows that the emission spectrum of **C3** changes progressively as a function of the excitation wavelength (Figure 7b). Notably, a structured spectrum featuring two relative maxima at 475 and 502 nm can be discerned upon excitation at 350 nm, which nicely matches the vibrational progressions observed in the phosphorescence of the complex at 77 K (Figure 6c) and can be thus associated with emission from a triplet LC excited state.

On the other hand, a less structured spectrum progressively forms along with the disappearance of the 475 nm band when moving the excitation towards longer wavelengths, until a featureless band with a maximum at 533 nm can be distinguished. This latter spectrum can be assigned to phosphorescence of triplet MLCT character. Hence, this experimental evidence supports the strong interplay between the triplet LC and MLCT previously envisioned and shows that for **C3**, the contribution of each emitting state also depends on the intrinsic population of the corresponding spin-allowed electronic transition.

## 3. Materials and Methods

### 3.1. General Information and Materials

The organic compounds and copper (I) complexes were synthesised according to the reported procedures [28]. All reagents and solvents were purchased from commercial sources unless otherwise specified and used as received. All ligands and complexes were characterised by spectroscopic and spectrometric analyses. NMR spectra were recorded on NMR Bruker AV 400 MHz. Chemical shifts are given in parts per million relatives to TMS (^1^H and ^13^C, δ(SiMe_4_) = 0) or an external standard [δ(CFCl_3_) = 0 for ^19^F NMR and δ(H_3_PO_4_) = 0 for ^31^P NMR]. Most NMR assignments were supported by additional 2D experiments. HRMS-ESI-MS experiments were carried out using a Thermo Scientific Exactive Plus Orbitrap Spectrometer. FT-IR spectra were recorded on a Shimadzu Tracer 100 spectrophotometer using KBr pellets. Purification of organic compounds was carried out by column chromatography using Merck silica gel 60 (70–230 mesh). UV-Vis spectra were registered using an Agilent Technologies UV-Vis-NIR spectrophotometer. Cyclic voltammograms were obtained using an Eco Chemie PGSAT 101 Potentiostat in a three-electrode cell configuration; the working electrode was a glassy carbon electrode, Pt was used as the counter electrode, and a silver wire was used as a quasi-reference electrode. Ferrocene (Fc) was added as an internal standard, and the potentials were referenced to the Fc^+^/Fc redox couple. The supporting electrolyte was 0.1 mol L^−1^ tetrabutylammonium hexafluorophosphate (TBAPF_6_) in dichloromethane, while a 1 mmol L^–1^ complex concentration was used. Photoluminescence spectra were taken on an Edinburgh Instrument spectrofluorometer. Time-resolved luminescence data were taken on a laser flash photolysis apparatus comprised of a Continuum Surelite II Nd:YAG laser (excitation at 355 nm, FWHM = 6–8 ns, was provided by THG from the 1064 nm fundamental). Light emitted by the sample was focused onto the entrance slit of a 300 mm focal length Acton SpectraPro 2300i triple grating, flat field, and double exit monochromator equipped with a photomultiplier detector (Hamamatsu R3896). The photomultiplier’s signals were processed by means of a TeledyneLeCroy 604Zi (400 MHz, 20 GS/s) digital oscilloscope. The CH_2_Cl_2_ solutions of metal complexes **C1-4** were purged using nitrogen gas for 20 min before steady-state and time-resolved emission measurements. Crystallographic data were collected on a Bruker APEX-II CCD diffractometer, using a graphite monochromated Mo Ka radiation (λ = 0.71073 Å), at 294(2) K. Data reduction was performed using SAINT V6.45A and SORTAV [43] in the diffractometer package. The collected data were corrected for Lorentz and polarization effects as well as absorption using SADABS software, version 2.03 [44]. The structural resolution procedure was conducted using SHELXT [45]. Full details can be found in the Electronic Supporting Information (*ESI*) and in the independently deposited crystallography information files (cif), CCDC numbers: 2304682 for **L3**, 2304684 for **C1**, 2304682 for **C3**, and 2304683 for **C4**.

### 3.2. General Synthetic Procedure for Bis(indazol-1-yl)methane Analogues ***L1-4***

To a solution of the corresponding indazole analogue in 20% hydrochloric acid, 40% formalin was added with stirring. A slurry was formed, and it was necessary to stir vigorously. After 1 h, the mixture was diluted with water. The precipitate was collected and washed with cold water. Recrystallisation from water and subsequent liquid–liquid extractions of the mother liquor yielded 92–98% of the corresponding l-(hydroxyalky1)azole analogue precursor. The latter and the corresponding azole analogue were poured into a one-necked round-bottomed flask equipped with a Dean–Stark water separator. The flask was charged with 10 mol% of *p*-toluenesulfonic acid (TsOH) and dried toluene. The mixture was heated under reflux for 12 h. After that, the solvent was evaporated under reduced pressure, and the crude product was crystallised from water/1,4-dioxane and the mother liquor was extracted to give the desired product. Samples for analysis were previously purified by chromatographic column with a gradient of 0% to 50% ethyl acetate–hexane mixtures as eluents.

Ligand **L2**. Yellowish-white-coloured solid, 88% yield. Melting point: 218.8–219.5 °C (not corrected). ^1^H NMR (400 MHz, CDCl_3_, 298 K) δ ppm: 7.27 (d, *J* = 8.0 Hz, 2H), 6.98 (d, *J* = 8.5 Hz, 2H), 6.57 (ddd, *J* = 8.4, 7.0, 1.1 Hz, 2H), 6.42 (ddd, *J* = 8.4, 7.0 1.1 Hz 2H), 6.22 (s, 2H), 3.19 (s, 6H). ^13^C{^1^H} NMR (101 MHz, CDCl_3_, 298 K) δ ppm: 162.8, 140.6, 136.6, 128.2, 124.2, 124.1, 122.3, 110.7, 63.5, 52.4. HRMS (ESI): calculated for C_19_H_16_N_4_O_4_: 364.1172, found: [M + 1] 365.1246.

Ligand **L3**. Pink-coloured solid, 91% yield. Melting point: 147.9–148.3 °C (not corrected). ^1^H NMR (400 MHz, CDCl_3_, 298 K) δ ppm: 8.44 (s, 2H), 8.11 (s, 2H), 8.08 (dd, *J* = 8.9, 1.5 Hz, 2H), 7.87 (d, *J* = 8.9 Hz, 2H), 6.92 (s, 2H), 3.89 (s, 6H). ^13^C{^1^H} NMR (101 MHz, CDCl_3_, 298 K) δ ppm: 167.3, 136.7, 128.3, 125.1, 124.8, 124.7, 110.4, 62.0, 52.6. HRMS (ESI): calculated for C_19_H_16_N_4_O_4_: 364.1172, found: [M + 1] 365.1245.

Ligand **L4**. Pale-yellow-coloured solid, 87% yield. Melting point: 249.6–250.4 °C (not corrected). ^1^H NMR (400 MHz, CDCl_3_, 298 K) δ ppm: 8.59 (s, 2H), 8.07 (s, 2H), 7.83 (dd, *J* = 8.5, 1.1 Hz, 2H), 7.71 (dd, *J* = 8.5, 1.1 Hz, 2H), 6.98 (s, 2H), 3.98 (s, 6H). ^13^C{^1^H} NMR (101 MHz, CDCl_3_, 298 K) δ ppm: 167.2, 139.2, 135.0, 129.1, 127.4, 122.4, 121.1, 112.3, 61.4, 52.5. HRMS (ESI): calculated for C_19_H_16_N_4_O_4_: 364.1172, found: [M + 1] 365.1248.

### 3.3. General Synthetic Procedure for Complexes ***C1-4***

To a solution of 1 equiv. of [Cu(CH_3_CN)]BF_4_ in CH_3_CN (3 mL), the respective **L1-4** ligand dissolved in CH_2_Cl_2_ (5 mL) was added dropwise under a nitrogen atmosphere. The reaction mixture was stirred for 30 min, and then a solution of 1 equiv. of DPEPhos in CH_2_Cl_2_ (3 mL) was added and stirred for another 30 min. After this time, the volatiles were removed under reduced pressure. The crude product was dissolved in CH_2_Cl_2_ and toluene was added, allowing it to crystallise at cold temperatures (−20 °C).

Complex **C1**. White-coloured solid, 76% yield. ^1^H NMR (400 MHz, CDCl_3_, 298 K) δ ppm: 8.26 (d, *J* = 8.5 Hz, 2H), 7.59 (t, *J* = 7.7 Hz, 2H), 7.37 (s, 2H), 7.32 (m, 2H), 7.26 (m, 12H), 7.18 (d, *J* = 8.0 Hz, 2H), 7.16 (dt, *J* = 8.1, 5.7 Hz, 8H), 7.15 (m, 2H), 7.13. (m, 2H), 7.07 (d, *J* = 5.9 Hz, 2H), 7.01 (t, *J* = 7.6 Hz, 2H), 6.83 (dtd, *J* = 7.9, 4.0, 1.6 Hz, 2H). ^13^C{^1^H} NMR (101 MHz, CDCl_3_, 298 K) δ ppm: 158.3 (t, *J^C−P^* = 6.0 Hz), 139.9, 138.0, 137.2, 134.7, 133.3 (t, *J^C−P^* = 8.0 Hz), 132.1, 130.7 (t, *J^C−P^* = 16.7 Hz), 130.5, 129.8, 129.1 (t, *J^C−P^* = 4.7 Hz), 128.8 (d, *J^C-P^* = 81.5 Hz), 125.4, 125.2, 124.4 (t, *J^C−P^* = 14.3 Hz), 122.8, 120.7, 120.3, 110.6, 57.9. ^19^F{^1^H} NMR (400 MHz, CDCl_3_, 298 K) δ ppm: −152.15. ^31^P{^1^H} NMR (160 MHz, CDCl_3_, 298 K) δ ppm = −13.36. ^11^B{^1^H} NMR (128 MHz, CDCl_3_, 298 K) δ ppm = −0.57. FT IR $\tilde{\nu }$_max_ cm^−1^ = 1620.2 (C=N), 1589.3 (C=C), 1396.5 (C–N), 1377.2 (C–N), 1057.0 (P–C), 590.2 (Cu–N), 532.4 (B–F). HRMS (ESI): calculated for [C_51_H_40_CuN_4_OP_2_]^+^: 849.1968, found: [M+] 849.1958.

Complex **C2**. Greenish-yellow-coloured solid, 81% yield. ^1^H NMR (400 MHz, CDCl_3_, 298 K) δ ppm: 8.03 (d, *J* = 8.1 Hz, 4H), 7.35 (q, *J* = 7.2 Hz, 12H), 7.23 (dd, *J* = 10.9, 4.3 Hz, 4H), 7.22–7.17 (m, 8H), 7.11–7.06 (m, 2H), 7.02 (t, *J* = 7.5 Hz, 2H), 6.88–6.85 (m, 2H), 6.82 (td, *J* = 7.8, 1.7 Hz, 2H), 6.68 (tdd, *J* = 8.5, 4.8, 2.6 Hz, 2H), 3.98–3.82 (m, 6H). ^13^C{^1^H} NMR (101 MHz, CDCl_3_, 298 K) δ ppm: 162.8, 157.8 (t, *J^C-P^* = 5.8 Hz), 140.6, 136.5, 134.4, 133.6 (t, *J^C-P^* = 8.1 Hz), 132.3, 130.9, 129.8, 129.2 (t, *J^C-P^* = 5.0 Hz), 129.1, 128.6, 128.3, 125.4, 125.2, 124.4, 123.7, 122.5 (t, *J^C-P^* = 16.6 Hz), 122.3, 120.0, 117.8, 110.6, 63.1, 52.5. ^19^F{^1^H} NMR (400 MHz, CDCl_3_, 298 K) δ ppm: −153.02. ^31^P{^1^H} NMR (160 MHz, CDCl_3_, 298 K) δ ppm = −14.42. ^11^B{^1^H} NMR (128 MHz, CDCl_3_, 298 K) δ ppm = −0.72. FT IR $\tilde{\nu }$_max_ cm^−1^ = 1720.5 (C=O), 1616.4 (C=N), 1570.1 (C=C), 1334.7 (C–N), 1203.6 (C–O), 1057.0 (P–C), 582.5 (Cu–N), 532.4 (B–F). HRMS (ESI): calculated for [C_55_H_44_CuN_4_O_5_P_2_]^+^: 965.2077, found: [M+] 965.2049.

Complex **C3**. Pink-coloured solid, 85% yield. ^1^H NMR (400 MHz, CDCl_3_, 298 K) δ ppm: 8.84 (s, 2H), 7.83 (d, *J* = 8.5 Hz, 2H), 7.51 (s, 2H), 7.44 (d, *J* = 8.5 Hz, 2H), 7.30 (d, *J* = 8.1 Hz, 2H), 7.25 (dd, *J* = 10.3, 6.9 Hz, 2H), 7.16 (t, *J* = 7.0 Hz, 12H), 7.09 (s, 2H), 7.08–7.04 (m, 8H), 7.00 (d, *J* = 7.8 Hz, 2H), 6.81 (p, *J* = 4.0 Hz, 2H), 4.03 (s, 6H). ^13^C{^1^H} NMR (101 MHz, CDCl_3_, 298 K) δ ppm: 166.8, 158.2 (t, *J^C-P^* = 6.0 Hz), 139.3, 138.0, 137.6, 134.7, 133.2 (t, *J^C-P^* = 8.0 Hz), 132.3, 131.5, 130.6, 130.3 (t, *J^C-P^* = 17.2 Hz), 129.2–129.1, 128.3, 125.4 (d, *J^C-P^* = 8.2 Hz), 123.8 (t, *J^C-P^* = 14.8 Hz), 123.4, 121.3, 120.3, 112.1, 58.4, 52.8. ^19^F{^1^H} NMR (400 MHz, CDCl_3_, 298 K) δ ppm: −152.52. ^31^P{^1^H} NMR (160 MHz, CDCl_3_, 298 K) δ ppm = −12.93. ^11^B{^1^H} NMR (128 MHz, CDCl_3_, 298 K) δ ppm = −0.67. FT IR $\tilde{\nu }$_max_ cm^−1^ = 1716.7 (C=O), 1620.2. (C=N), 1570.1 (C=C), 1377.2 (C–N), 1195.9 (C–O), 1072.4 (P–C), 578.6 (Cu–N), 509.2 (B–F). HRMS (ESI): calculated for [C_55_H_44_CuN_4_O_5_P_2_]^+^: 965.2077, found: [M+] 965.2061.

Complex **C4**. White-coloured solid, 87% yield. ^1^H NMR (400 MHz, CDCl_3_, 298 K) δ ppm: 8.82 (s, 2H), 7.83 (dd, *J* = 8.5, 1.1 Hz, 2H), 7.55 (s, 2H), 7.47 (d, *J* = 8.5 Hz, 2H), 7.30 (dq, *J* = 8.0, 3.1, 2.6 Hz, 2H), 7.22 (t, *J* = 7.5 Hz, 12H), 7.15–7.10 (m, 8H), 7.13 (m, 2H), 7.08 (s, 2H), 7.00 (t, *J* = 7.6 Hz, 2H), 6.79 (dtd, *J* = 8.0, 4.2, 1.6 Hz, 2H), 4.02 (s, 6H). ^13^C{^1^H} NMR (101 MHz, CDCl_3_, 298 K) δ ppm: 166.7, 158.1 (t, *J^C-P^* = 6.0 Hz), 139.2, 137.5, 134.6, 133.3 (t, *J^C-P^* = 8.0 Hz), 132.2, 131.4, 130.5, 130.4 (t, *J^C-P^* = 17.2 Hz), 129.1 (t, *J^C-P^* = 4.8 Hz), 125.4, 123.7 (t, *J^C-P^* = 14.9 Hz), 123.3, 122.8 (d, *J^C-P^* = 497.9 Hz), 121.3, 117.7, 112.0, 58.5, 52.8. ^19^F{^1^H} NMR (400 MHz, CDCl_3_, 298 K) δ ppm: −153.0. ^31^P{^1^H} NMR (160 MHz, CDCl_3_, 298 K) δ ppm = −13,19. ^11^B{^1^H} NMR (128 MHz, CDCl_3_, 298 K) δ ppm = −0.73. FT IR $\tilde{\nu }$_max_ cm^−1^ = 1720.5 (C=O), 1620.2 (C=N), 1573.9 (C=C), 1357.9 (C–N), 1207.4 (C–O), 1084.0 (P–C), 624.9 (Cu–N), 513.1 (B–F). HRMS (ESI): calculated for [C_55_H_44_CuN_4_O_5_P_2_]^+^: 965.2077, found: [M+] 965.2044.

### 3.4. Computational Details

The molecular structure of complexes **C1-4** were obtained with proper optimisation at the B3LYP-D3/6-31g(d,p)-LANL2DZ(Cu) level of theory. The level of theory was selected since it nicely predicts the λ_max_ (384 nm in CH_2_Cl_2_) for a related [Cu(bpy)(xantphos)]+ complex to be 388 nm [46]. On the other hand, frequency calculations were performed to corroborate the nature of the systems as a minimum on the potential energy surface, giving only the real frequencies for all four systems. Aiming to match the experimental conditions, all computations were performed in CH_2_Cl_2_ using the polarisable continuum model (PCM) [47]. Excited states for each system were computed using time-dependent density functional theory (TD-DFT) at the same level of theory. A collection of the first 50 excited states were calculated for each system. All quantum chemistry calculations were carried out in the Gaussian 16 software package [48]. Atomic contributions to molecular orbitals were obtained utilising the natural atomic orbital (NAO) basis, and hole–electron distributions were computed using Multiwfn software, version 3.7 [49].

## 4. Conclusions

The successful synthesis and characterisation of a series of bis(1*H*-indazol-1-yl)methane bidentate ligands and their respective copper heteroleptic–cationic complexes [Cu(*N,N*)(*P,P*)]BF_4_ was described. The four complexes were obtained in good yields and were stable in air and water in the solid state and solution. The electrochemical characterisation of **C1-4**, supported by DFT calculations, showed that the oxidation process involves the copper centre and is independent of the presence and position of the methoxycarbonyl groups in the *N,N* ligand, while the reduction mainly occurs on the *N,N* ligand and is affected by the presence and position of the –COOCH_3_ groups. All complexes absorbed mainly in the UV region and exhibited green-yellow luminescence in degassed CH_2_Cl_2_ solution. **C1** showed a structured emission assignable to phosphorescence from a triplet excited state of LC nature involving the *N*,*N* ligand, while complexes **C2-4** displayed phosphorescence from a triplet MLCT excited state which resulted at lower energies than the local LC state due to the presence of the electron-withdrawing groups. The presence of a closely lying triplet LC state in complexes **C2-4** was, however, confirmed by emission measurements at 77 K and πobserved to influence the emission behaviour of the complexes. A luminescence quantum yield of 2.5% in solution conditions was attained with complex **C4**.

The results collected here highlight the potential of heteroleptic copper(I) complexes based on derivatives of the bis(1*H*-indazol-1-yl)methane ligand for the obtainment of noble luminescent metal compounds and how the chemical modification of the *N,N* ligand appears critical to finely tune the photophysical properties of the resulting metal complexes towards profitable applications, e.g., in luminescent devices or photocatalysis.

## Figures and Tables

**Table 1:** Electrochemical characterisation of complexes **C1-4** in CH_2_Cl_2_ ^a^.

Complex	E_ox_/V vs. Fc^+^/Fc ^b^	E_red_/V vs. Fc^+^/Fc ^b^	ΔE_ox/red_/V
**C1**	0.68	−1.41	2.09
**C2**	0.65	−1.32	1.97
**C3**	0.64	−1.30	1.94
**C4**	0.68	−1.22	1.90

^a^ Measured by CV in CH_2_Cl_2_ with TBAPF_6_ as the supporting electrolyte, scan rate 0.1 V/s, using glassy carbon as the WE, platinum as the CE, Ag wire as a quasi-reference, and ferrocene as an internal standard; ^b^ peak potentials were given due to the irreversible nature of the redox process.

**Table 2:** Properties of the first singlet excited states of complexes under study ^a^.

	State	Absorption λ [nm] (E [eV])	*f*	Monoexcitations (CI Coef. and% Contribution)	Description of the Electronic Transition
**C1**	S_1_	349 (3.55)	0.059	H→L (0.70; 98%)	Cu(*d*) + *P,P*(π) → *N,N*(π*); ^1^MLCT/^1^LLCT
**C2**	S_1_	415 (2.99)	0.004	H–1→L (−0.37; 27%) H→L (0.59; 71%)	Cu(*d*) + *P,P*(π) + *N,N*(π) → *N,N*(π*); ^1^MLCT/^1^LLCT/^1^IL Cu(*d*) + *P,P*(π) → *N,N*(π*); ^1^MLCT/^1^LLCT
	S_2_	404 (3.06)	0.040	H–1→L (0.59; 70%) H→L (0.37; 27%)	Cu(*d*) + *P,P*(π) + *N,N*(π) → *N,N*(π*); ^1^MLCT/^1^LLCT/^1^IL Cu(*d*) + *P,P*(π) → *N,N*(π*); ^1^MLCT/^1^LLCT
	S_3_	384 (3.23)	0.008	H–1→L + 1 (−0.16, 5%) H→L + 1 (0.67, 91%)	Cu(*d*) + *P,P*(π) + *N,N*(π) → *N,N*(π*); ^1^MLCT/^1^LLCT/^1^IL Cu(*d*) + *P,P*(π) → *N,N*(π*); ^1^MLCT/^1^LLCT
	S_4_	378 (3.28)	0.030	H–1→L + 1 (0.68; 91%) H→L + 1 (0.16; 5%)	Cu(*d*) + *P,P*(π) + *N,N*(π) → *N,N*(π*); ^1^MLCT/^1^LLCT/^1^IL Cu(*d*) + *P,P*(π) → *N,N*(π*); ^1^MLCT/^1^LLCT
**C3**	S_1_	369 (3.36)	0.043	H→L (0.69; 96%)	Cu(*d*) + *P,P*(π) → *N,N*(π*); ^1^MLCT/^1^LLCT
	S_2_	345 (3.59)	0.054	H→L + 1 (0.69; 96%)	Cu(*d*) + *P,P*(π) → *N,N*(π*); ^1^MLCT/^1^LLCT
**C4**	S_1_	397 (3.12)	0.073	H→L (0.70; 98%)	Cu(*d*) + *P,P*(π) → *N,N*(π*); ^1^MLCT/^1^LLCT
	S_2_	365 (3.40)	0.036	H→L + 1 (0.70; 98%)	Cu(*d*) + *P,P*(π) → *N,N*(π*); ^1^MLCT/^1^LLCT

^a^ Excited states for the most intense transitions are listed. Transition energies (E), oscillator strength (f), configuration interaction (CI) coefficient, and contribution percentage to the excited state wave function. H and L stand for HOMO and LUMO, respectively. Orbital composition analysis is provided in Table S9 in the *ESI*. Values were obtained at the B3LYP-D3/6-31G(d,p)-LANL2DZ(Cu) in CH_2_Cl_2_.

**Table 3:** Summary of the luminescence data of complexes **C1-4**.

Complex	CH_2_Cl_2_ at Room Temperature			77 K
	λ_em_ [nm]	Φ/%	τ [μs] ^d^	λ_em_ [nm]
**C1**	440, 466, 495	2.2 ^a^	4.2	431, 489, 490, 525
**C2**	545	<0.1 ^b^	1.3, 10	449, 477, 514
**C3**	473, 533	0.3 ^b,c^	1.8, 7.2	435, 464, 495, 531
**C4**	574	2.5 ^b^	2.9, 7.7	455, 489, 522

^a^ Estimated using fluorescein in 0.1 M NaOH as a standard (Φ = 94%); ^b^ estimated using Ru(bpy)_3_Cl_2_·6H_2_O in water as a standard (Φ = 2.8%); ^c^ excitation at 363 nm; ^d^ laser excitation at 355 nm.

## Data Availability

Data are contained within the article and supplementary materials.

## Supplementary Materials

The following supporting information can be downloaded at: https://www.mdpi.com/article/10.3390/molecules29010047/s1. (Figures S1–S57 and Tables S1–S9).
