# Supplementary material for: Air- and Water-Stable Heteroleptic Copper (I) Complexes Bearing Bis(indazol-1-yl)methane Ligands: Synthesis, Characterisation, and Computational Studies
Source: Molecules. 2023 Dec 20;29(1):47. doi: 10.3390/molecules29010047 (PMC10780253; doi:10.3390/molecules29010047)
Supplement: Supplementary file 1 [file molecules-29-00047-s001.zip › molecules-2748511-supplementary.pdf]

ELECTRONIC SUPPORTING INFORMATION

Air and water-stable heteroleptic copper (I) complexes bearing bis(indazol-1-yl)methane ligands: synthesis, characterisation, and computational studies

David Moreno da Costa,[a,*] César Zúñiga Loyola,[b] Federico Droghetti,[c] Stephania Robles,[b] Alondra Villegas-Menares,[a] Nery Villegas-Escobar,[d] Ivan Gonzalez P.,[e] Elies Molins,[f] Mirco Natali,[c,*] and Alan R. Cabrera[a,*]

[a] *Departamento de Química Inorgánica, Facultad de Química y de Farmacia, Pontificia Universidad Católica de Chile, Avenida Vicuña Mackenna 4860, Macul, Santiago, Chile.*

[b] *Departamento de Química de Los Materiales, Facultad de Química y Biología, Universidad de Santiago de Chile, Casilla 40, Correo 33, Sucursal Matucana, Santiago 9170022, Chile.*

[c] *Department of Chemical, Pharmaceutical and Agricultural Sciences, University of Ferrara, via L. Borsari 46, 44121 Ferrara, Italy.*

[d] *Departamento de Físico-Química, Facultad de Ciencias Químicas, Universidad de Concepción, Concepción, Chile.*

[e] *Departamento de Química, Facultad de Ciencias Naturales, Matemática y del Medio Ambiente, Universidad Tecnológica Metropolitana, Las Palmeras 3360, Ñuñoa, Santiago 7800003, Chile.*

[f] *Institut de Ciència de Materials de Barcelona, Consejo Superior de Investiggaciones Científicas, Campus de la UAB, 08193 Cerdanyola, Barcelona, Spain.*

*Corresponding Author

E-mail address: drmoreno@uc.cl; ntlmrc@unife.it; arcabrer@uc.cl

TABLE OF CONTENTS

1. SYNTHESIS AND STRUCTURAL CHARACTERISATION ______ 3

1.1 General considerations ______ 3

1.2 General synthesis of bis(indazol-1-yl)methane analogues. ______ 4

1.2.1 Dimethyl 1,1'-methylenebis(1*H*-indazole-3-carboxylate) (L2) ______ 5

1.2.2 Dimethyl 1,1'-methylenebis(1*H*-indazole-5-carboxylate) (L3) ______ 8

1.2.3 Dimethyl 1,1'-methylenebis(1*H*-indazole-6-carboxylate) (L4) ______ 13

1.3 General synthesis of heteroleptic Cu(I) complexes. ______ 16

1.3.1 Compound C1 ______ 16

1.3.2 Compound C2 ______ 24

1.3.3 Compound C3 ______ 29

1.3.4 Compound C4 ______ 36

2. UV-VISIBLE SPECTROSCOPY ______ 43

3. ELECTROCHEMICAL MEASUREMENTS ______ 45

4. EXCITED STATE LIFETIME MEASUREMENTS ______ 47

5. COMPUTATIONAL CALCULATIONS ______ 50

5.1 Orbital Composition Analysis ______ 50

5.2 Cartesian Coordinates (atom X Y Z) ______ 51

1. SYNTHESIS AND STRUCTURAL CHARACTERISATION

1.1 General considerations

Reagents were purchased from commercial sources and were used without further purification. The chromatography columns were performed on silica gel Merck 60 (70–230 mesh) (Merck, Darmstadt, Germany). NMR spectra were recorded on a Bruker AVANCE 400 instrument. Chemical shifts are given in parts per million relative to TMS [^{1}H and ^{13}C, $\delta(SiMe_4) = 0$] or an external standard [$\delta(CFCl_3) = 0$ for ^{19}F NMR, $\delta(H_3PO_4) = 0$ for ^{31}P NMR, and $\delta(BF_3) = 0$ for ^{11}B NMR]. Infrared spectra (ν_{max}) were recorded on an FT-IR Shimadzu Tracer 100 spectrophotometer, using potassium bromide (KBr) as the solid matrix. Melting points were measured on a Stuart Scientific SMP3 apparatus and were uncorrected. High-resolution mass spectra were obtained using a Thermo Finnigan mass spectrometer Model MAT 95XP. The ultraviolet-visible spectra (UV-Vis) were recorded in an Agilent Technologies UV-Vis-NIR spectrophotometer, using 0.025, 0.02, 0.015, 0.01, and 0.005 mmol L^{-1} concentration dilutions from a 1 mmol L^{-1} stock solution of each complex in anhydrous and degassed dichloromethane, at 25 ºC. Cyclic voltammograms were obtained using an Eco Chemie PGSAT 101 Potentiostat in a three-electrode cell configuration; the working electrode was a glassy carbon electrode, Pt was used as the counter electrode, and a silver wire was used as a quasi-reference electrode. Ferrocene was added as an internal standard, and the potentials were referenced to the Fc^{+}/Fc redox couple. The supporting electrolyte was 0.1 mol L^{-1} tetrabutylammonium hexafluorophosphate ($TBAPF_6$) in dichloromethane, while a 1 mmol L^{-1} complex concentration was used. Time-resolved luminescence data were taken on a laser flash photolysis apparatus comprised of a Continuum Surelite II Nd:YAG laser (excitation at 355 nm, FWHM = 6–8 ns, was provided by THG from the 1064-nm fundamental). Light emitted by the sample was focused onto the entrance slit of a 300 mm focal length Acton SpectraPro 2300i triple grating, flat field, and double exit monochromator equipped with a photomultiplier detector (Hamamatsu R3896). Signals from the photomultiplier were processed using a TeledyneLeCroy 604Zi (400 MHz, 20 GS/s) digital oscilloscope. The CH_2Cl_2 solutions of metal complexes **C1-4** were purged using nitrogen gas for 20 minutes before steady-state and time-resolved emission measurements.

Single-crystal X-ray diffraction data were recorded on a Bruker APEX-II CCD diffractometer, using a graphite monochromated Mo Kα radiation (λ = 0.71073 Å). Crystallographic data were collected at 294(2) K. Data reduction was performed using SAINT V6.45A and SORTAV[1] in the diffractometer package. Data were corrected for Lorentz and polarization effects and for absorption by SADABS.[2] The structural resolution procedure was made using SHELXT.[3] Non-hydrogen atoms were refined anisotropically. Hydrogen atoms were introduced in calculated positions and refined riding on their parent atoms. Selected crystal and data collection parameters are reported in the corresponding Table 1.

1.2 General synthesis of bis(indazol-1-yl)methane analogues.

The bis(indazol-1-yl)methane analogues **L1-4** were synthesised according to the procedure of Ballesteros *et al.*,[4] where to a solution of indazole analogue in 20 % hydrochloric acid with stirring was added 40 % formalin. A slurry is formed, and it is necessary to stir vigorously. After 1 h, the mixture was diluted with water and put aside for 1 h. The precipitate was collected and washed with cold water. Recrystallization from water yielded 80-90 % of the corresponding l-(hydroxyalky1)azole according to the indazole analogue precursor. Due to the less solubility in water, the aqueous phases were extracted three times with dichloromethane, and the organic phases were dried and evaporated under vacuum to obtain an extra 8-12 % of the corresponding product. No further purification was realized. The 1-(hydroxyalky1)azole crude products, and the corresponding azole analogue, were poured into a one-necked round-bottomed flask equipped with a Dean-Stark water separator. The flask was charged with 10 mol% of *p*-toluenesulfonic acid (TsOH) and dried toluene. The mixture was heated under reflux and monitored by TLC up to the total consumption of the starting materials or the no advance of the reaction. After that, the solvent was evaporated under reduced pressure, and the crude was crystallised from water/1,4-dioxane. The solids obtained were dry by air, and the liquid phases were portioned in water/dichloromethane, and extracted

1 Blessing, R. H. *Acta Crystallogr., Sect. A: Found. Adv.* **1995**, *51*, 33-38.

2 SAINT, Bruker, A. X. S., Ed.; Inc.: Madison, Wisconsin, USA.

3 Sheldrick, G. M. *Acta Crystallogr., Sect. A: Found. Adv.* **2015**, *71*, 3-8.

4 Ballesteros, P. and Elguero, J., *Tetrahedron*, **1985**, *41*, 24, 5955 – 5963.

for further drying under reduced pressure. Samples for analysis were previously purified by chromatographic column with a gradient of 0 % to 50 % ethyl acetate – hexanes mixtures as eluents.

1.2.1 Dimethyl 1,1'-methylenebis(1*H*-indazole-3-carboxylate) (L2)

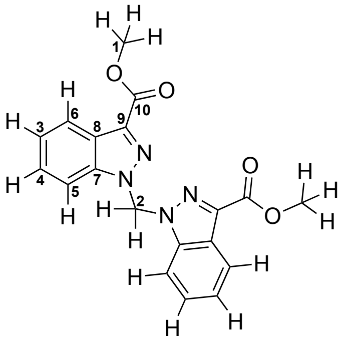

A pale-yellow solid is obtained with an 88 % yield following the general procedure.

^{1}H-NMR (400 MHz, $CDCl_3$, 298 K) δ ppm = 7.27 (d, *J* = 8.0 Hz, 2H, H_6), 6.98 (d, *J* = 8.5 Hz, 2H, H_5), 6.57 (ddd, *J* = 8.4, 7.0, 1.1 Hz, 2H, H_4), 6.42 (ddd, *J* = 8.4, 7.0 1.1 Hz 2H, H_3), 6.22 (s, 2H, H_2), 3.19 (s, 6H, H_1).

^{13}C{^{1}H}-NMR (101 MHz, $CDCl_3$, 298 K) δ ppm = 162.8 (C_{10}), 140.6 (C_7), 136.6 (C_9), 128.2 (C_4) 124.2 (C_8), 124.1 (C_3), 122.3 (C_6), 110.7 (C_5), 63.5 (C_2), 52.4 (C_1).

FT-IR (*w* weak, *m* medium, *s* strong) $\tilde{\nu}_{max}$ cm^{-1} = 3059.1 (*w*, stretching C*sp*^{2}-H), 2993.5 (*w*, stretching C*sp*^{2}-H), 2947.2 (*w*, stretching C*sp*^{3}-H), 1728.2 (*s*, stretching C=O ester), 1616.4 (*s*, stretching C=N), 1577.8 (*m*, n.a.), 1489.0 (*s*, bending C-H methylene group), 1442.8 (*s*, bending C-H methyl group), 1408.0 (*s*, n.a.), 1338.6 (*m*, stretching C-N), 1269.2 (*m*, stretching C-N), 1253.7 (*s*, stretching Ar-C-O ester), 1192.0 (*s*, stretching C-O), 1149.6 (*s*, n.a.), 1111.0 (*s*, n.a.), 1033.9 (*s*, n.a.), 1006.8 (*s*, n.a.), 976.0 (*s*, n.a.), 941.3 (*s*, n.a.), 825.5 (*s*, stretching C-N heterocycle), 744.5 (*s*, stretching C-H 1,2-disubstituted Ar,), 690.5 (*m*, n.a.), 621.1 (*s*, n.a.), 578.6 (*s*, n.a.), 428.2 (*s*, n.a.).

Melting point = 218.8 – 219.5 ºC (not corrected)

HRMS (ESI) = calculated for $C_{19}H_{16}N_4O_4$: 364.1172, found: [M+1] 365.1246.

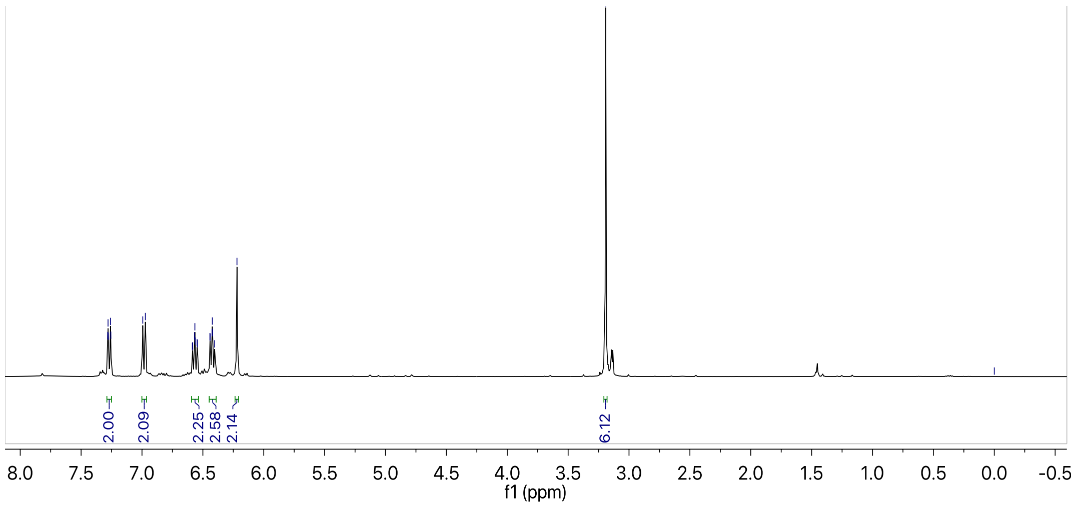

Figure S1. ^{1}H-NMR (400 MHz, $CDCl_3$, 298 K) spectrum of **L2**.

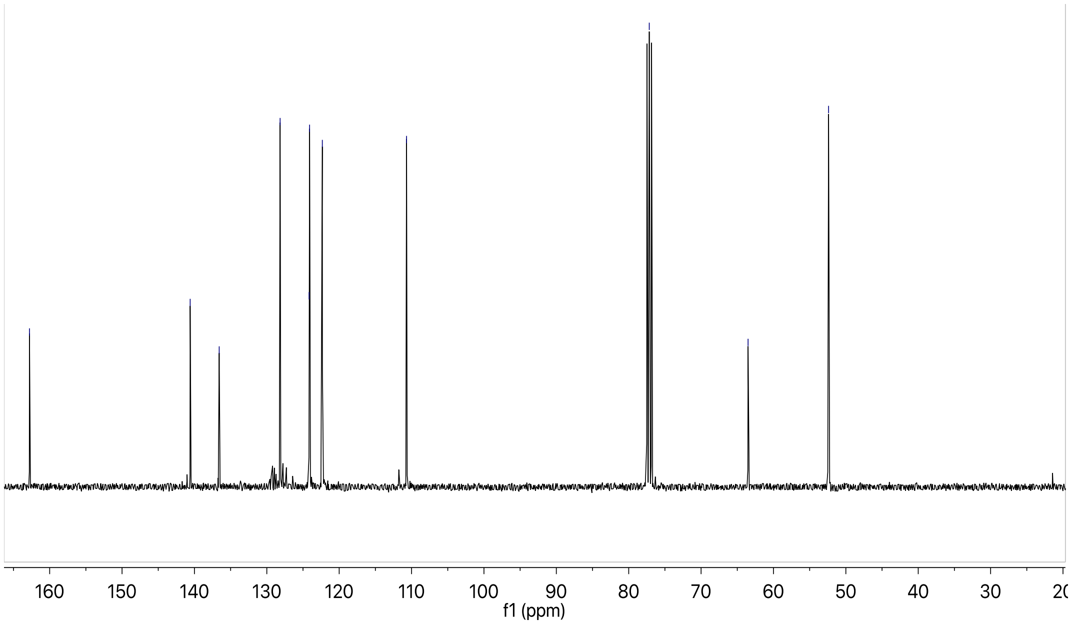

Figure S2. $^{13}C\{^{1}H\}$-NMR (101 MHz, $CDCl_3$, 298 K) spectrum of **L2**.

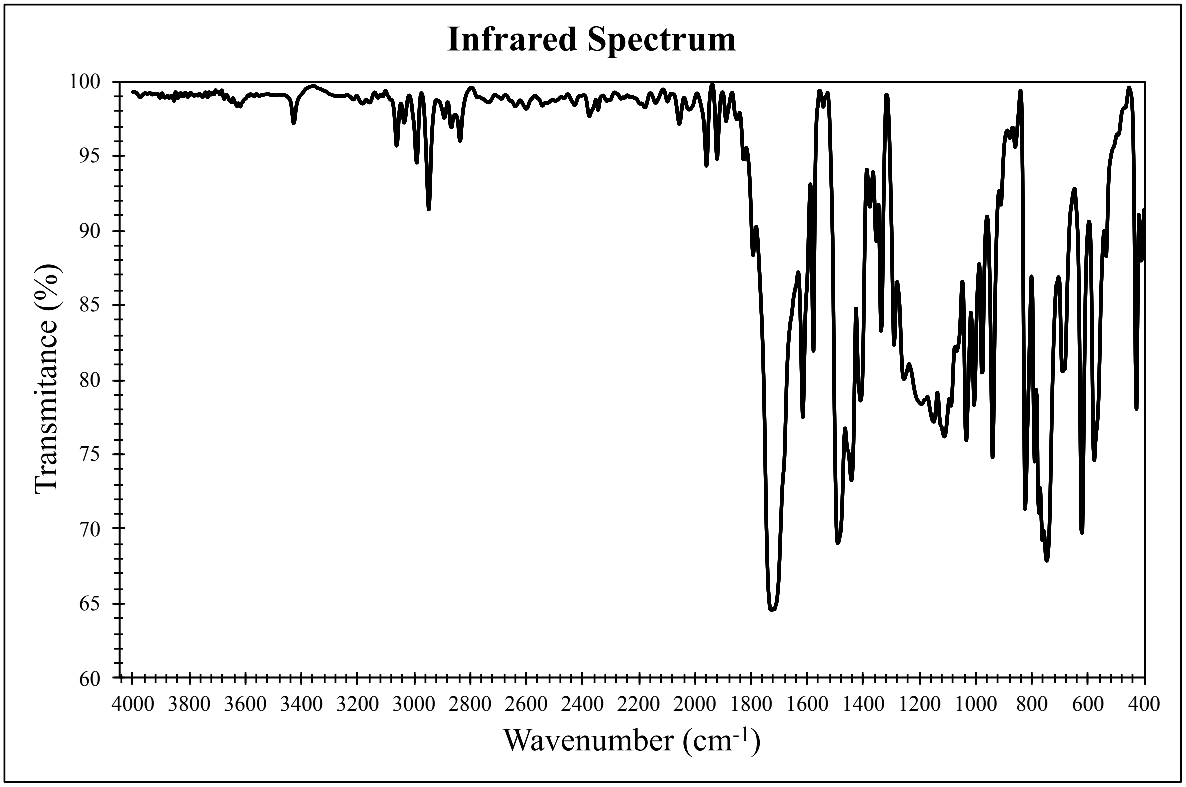

Figure S3. Infrared spectrum of **L2**.

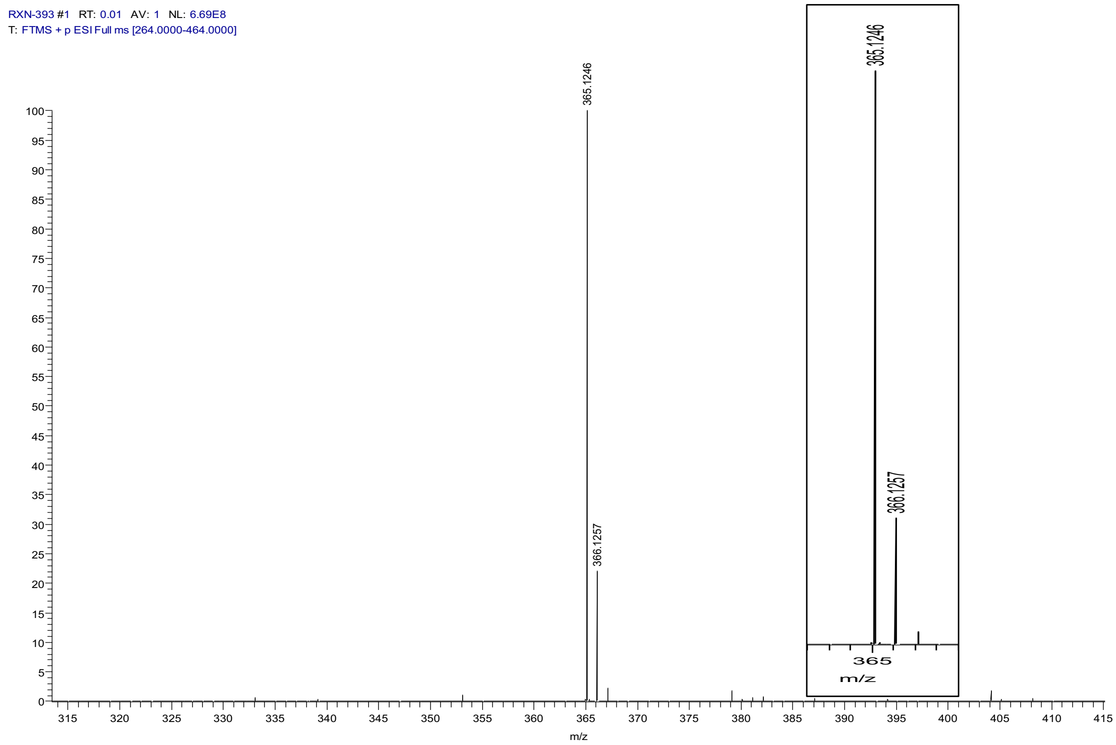

Figure S4. HRMS analysis of **L2** (positive ion mode).

1.2.2 Dimethyl 1,1'-methylenebis(1*H*-indazole-5-carboxylate) (L3)

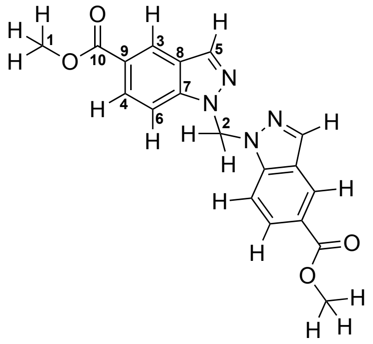

Following the general procedure, an orange-pink solid is obtained with a 91 % yield.

^{1}**H-NMR** (400 MHz, $CDCl_3$) δ ppm = 8.44 (s, 2H, H_6), 8.11 (s, 2H, H_5), 8.08 (dd, *J* = 8.9, 1.5 Hz, 2H, H_4), 7.87 (d, *J* = 8.9 Hz, 2H, H_3), 6.92 (s, 2H, H_2), 3.89 (s, 6H, H_1).

^{13}**C{^{1}H}-NMR** (101 MHz, $CDCl_3$) δ ppm = 167.3 (C_{10}), 136.7 (C_5), 128.3 (C_4), 125.1 (C_8), 124.8 (C_6), 124.7 (C_7), 110.4 (C_3), 62.0 (C_2), 52.6. (C_1).

FT-IR (*w* weak, *m* medium, *s* strong) $\tilde{\nu}_{max}$ cm^{-1} = 3063.0 (*w*, stretching Csp^2-H), 2958.8 (*w*, stretching Csp^3-H), 1720.5 (*s*, stretching C=O ester), 1616.4 (*m*, stretching C=N), 1500.6 (*s*,n.a.), 1462.0 (*s*, bending C-H methylene group), 1419.6 (*s*, bending C-H methyl group group), 1415.8 (*s*, n.a.), 1357.9 (*s*, stretching C-N) 1342.5 (*m*, stretching C-N), 1280.7 (*s*, stretching Ar-C-O ester), 1203.6 (*s*, stretching C-O ester), 1165.0 (*s*, n.a.), 1149.6 (*s*, n.a.), 1099.4 (*m*, n.a.), 1095.6 (*m*, n.a.), 1010.7 (*w*, n.a.), 1003.0 (*m*, n.a.), 933.6 (*s*, n.a.), 906.5 (*s*, n.a.), 856.4 (*m*, n.a.), 829.4 (*s*, stretching C-N heterocycle), 740.6 (*s*, stretching C-H 1,2-disubstituted Ar), 736.8 (*s*, n.a.), 628.8 (*w*, n.a.), 613.3 (*m*, n.a.), 609.5 (*s*, n.a.), 590.2 (*m*, n.a.), 470.6 (*w*, n.a.), 428.2 (*s*, n.a.).

Melting point = 147.9 – 148.3 ºC (not corrected)

HRMS (ESI) = calculated for $C_{19}H_{16}N_4O_4$: 364.1172, found: [M+1] 365.1245.

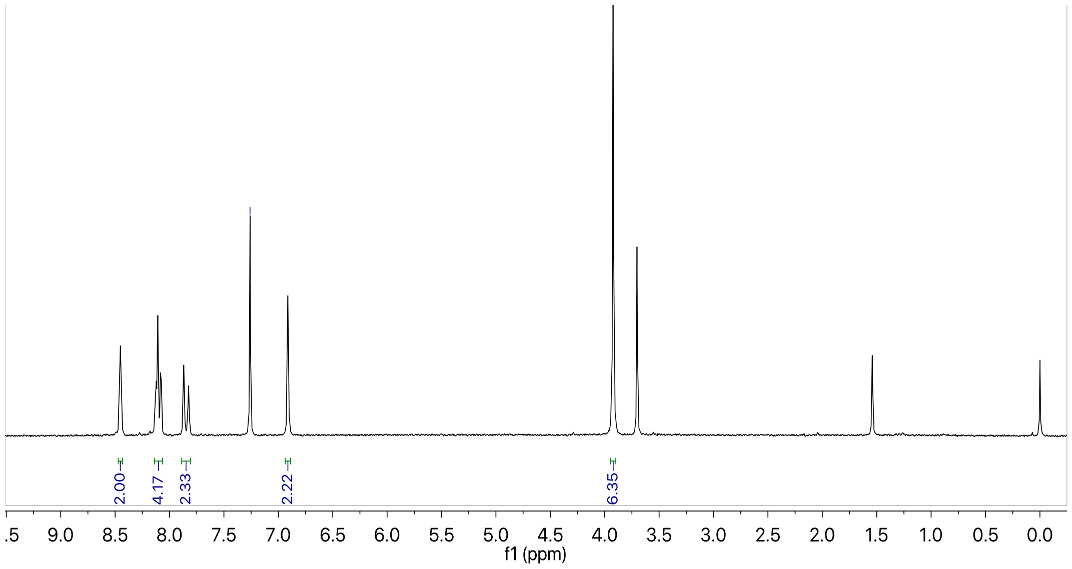

Figure S5. ^{1}H-NMR (400 MHz, $CDCl_3$, 298 K) spectrum of **L3**.

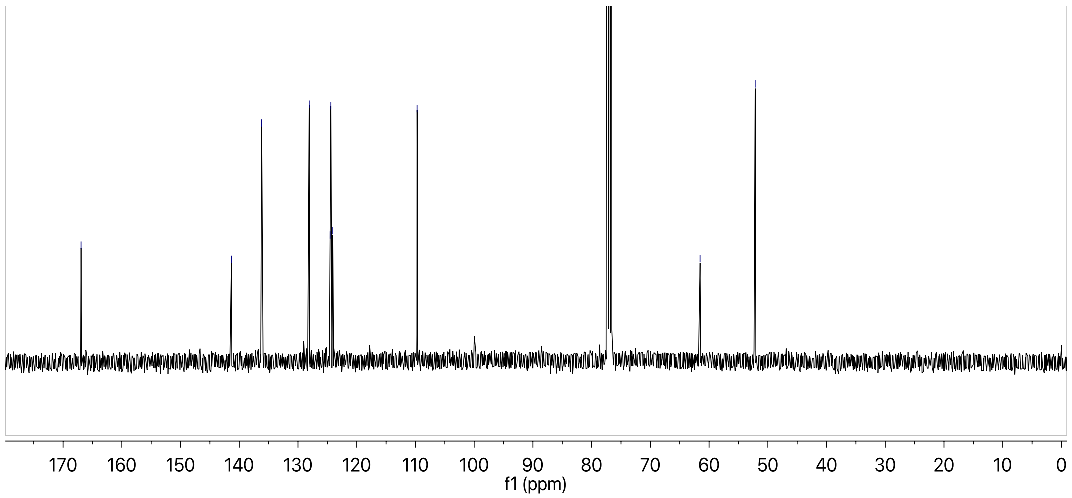

Figure S6. $^{13}C\{^{1}H\}$-NMR (101 MHz, $CDCl_3$, 298 K) spectrum of **L3**.

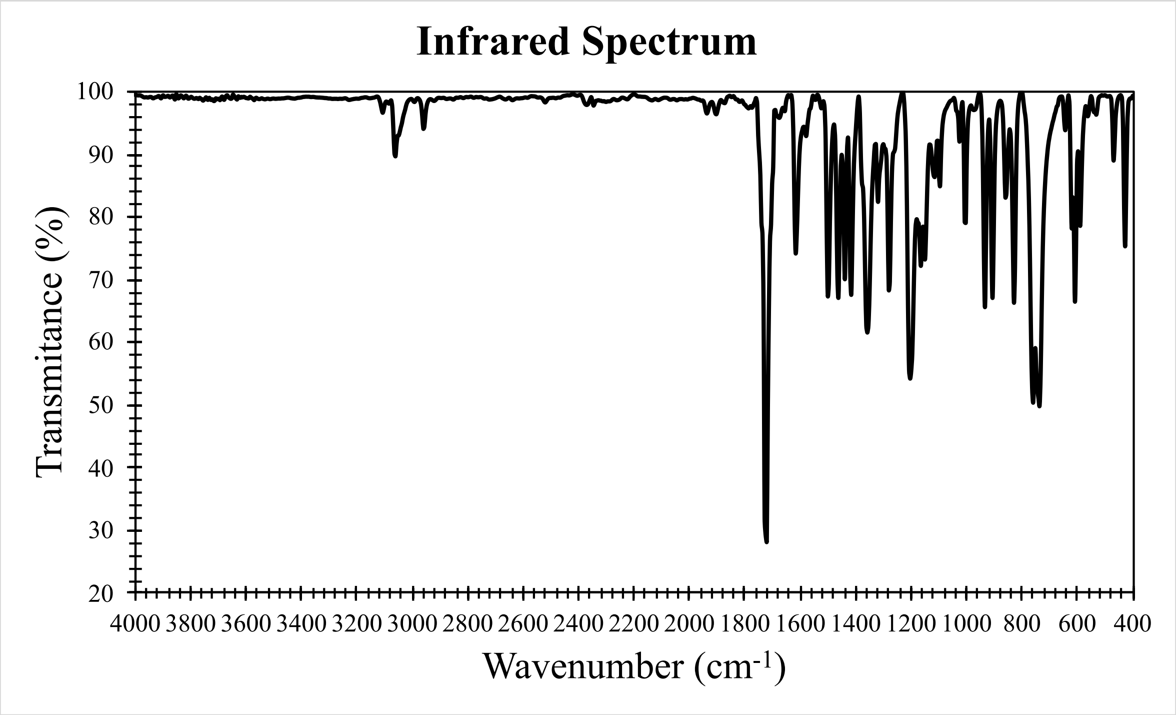

Figure S7. Infrared spectrum of **L3**.

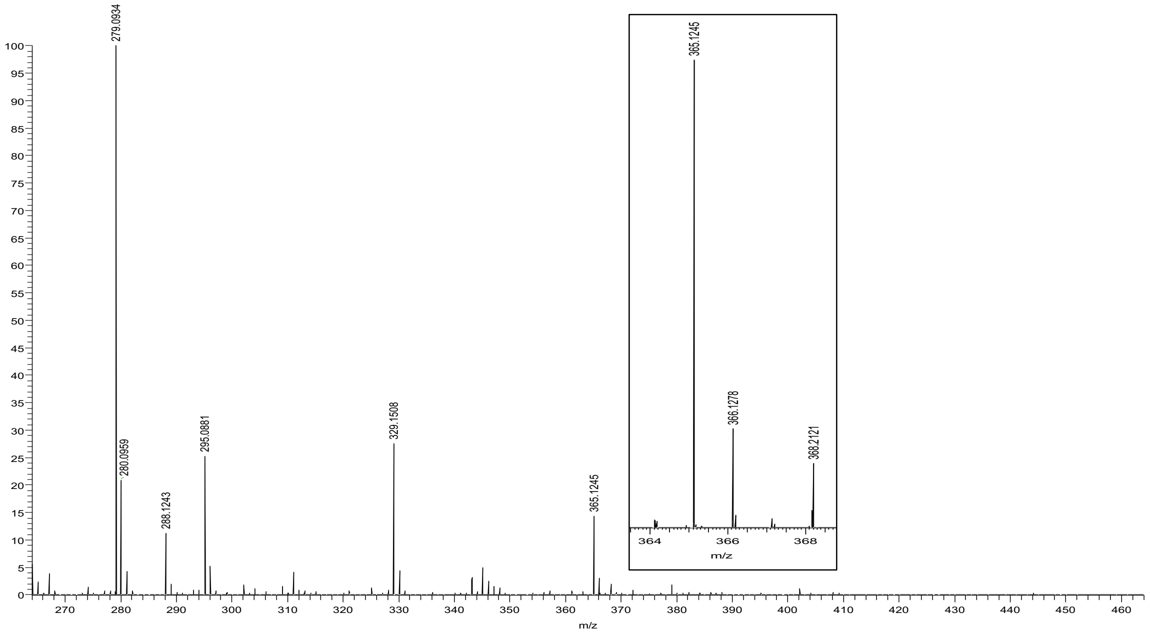

Figure S8. HRMS analysis of **L3** (positive ion mode).

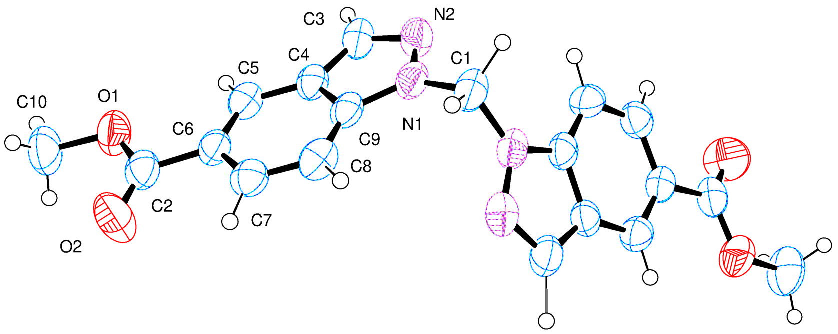

Figure S9. XRD molecular structure of **L3**.

Table S1. Selected crystallographic data of compound **L3**.

Bond distances

Atom 1	Atom 2	Length (A)
N1	N2	1.364(8)
N1	C1	1.444
N1	C9	1.388(7)
N2	C3	1.320(7)
C2	C6	1.496(8)
O1	C2	1.330(9)
O2	C2	1.196(9)

Selected Angles

Atom 1	Atom 2	Atom 3	Angle (º)
N2	N1	C1	120.2
N2	N1	C9	110.4(4)
N1	N2	C3	107.4(5)
N1	C1	N1	112.4
O1	C2	O2	124.6(6)
O1	C2	C6	112.1(5)

Selected Torsions

Atom 1	Atom 2	Atom 3	Atom 4	Torsion (º)
N1	N2	C3	C4	-0.3(6)
N1	C1	N1	N2	-93,4
N1	C1	N1	C9	85,9
O1	C2	C6	C5	-7.8(8)
C3	C4	C5	C6	-179.9(6)

Table S2. Crystal data and structure refinement for **L3**.

Identification code	Ch88
Empirical formula	C19 H16 N4 O4
Formula weight	364.36
Temperature	295(2) K
Wavelength	0.71073 Å
Crystal system	Orthorhombic
Space group	P 21 21 2
Unit cell dimensions	a = 18.333(6) Å α = 90°
	b = 11.202(4) Å β = 90°
	c = 4.0991(14) Å γ = 90°
Volume	841.8(5) $Å^3$
Z	2
Density (calculated)	1.437 Mg/m^3
Absorption coefficient	0.104 mm^{-1}
F(000)	380
Crystal size	0.32 x 0.09 x 0.08 mm^3
Theta range for data collection	2.130 to 28.524°.
Index ranges	-24<=h<=24, -14<=k<=14, -5<=l<=5
Reflections collected	20899
Independent reflections	2130
Completeness to theta = 25.242°	100.0 %
Absorption correction	Semi-empirical from equivalents
Max. and min. transmission	1 and 0.725
Refinement method	Full-matrix least-squares on F^2
Data / restraints / parameters	2130 / 6 / 155
Goodness-of-fit on F^2	0.751
Final R indices [I>2sigma(I)]	R1 = 0.0566, wR2 = 0.1104
R indices (all data)	R1 = 0.1850, wR2 = 0.1463
Largest diff. peak and hole	0.151 and -0.192 e.$Å^{-3}$

1.2.3 Dimethyl 1,1'-methylenebis(1*H*-indazole-6-carboxylate) (L4)

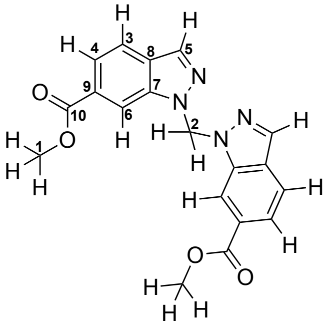

A white solid is obtained with an 87 % yield following the general procedure.

^{1}H-NMR (400 MHz, $CDCl_3$, 298 K) δ ppm = 8.59 (s, 2H, H_6), 8.07 (s, 2H, H_5), 7.83 (dd, *J* = 8.5, 1.1 Hz, 2H, H_4), 7.71 (dd, *J* = 8.5, 1.1 Hz, 2H, H_3), 6.98 (s, 2H, H_2), 3.98 (s, 6H, H_1).

^{13}C{^{1}H}-NMR (101 MHz, $CDCl_3$, 298 K) δ ppm = 167.2. (C_{10}), 139.2 (C_9), 135.0 (C_5), 129.1 (C_8), 127.4 (C_7), 122.4 (C_4), 121.1 (C_3), 112.3 (C_6), 61.4 (C_2), 52.5 (C_1).

FT-IR (*w* weak, *m* medium, *s* strong) $\tilde{\nu}_{max}$ cm^{-1} = 3414.0 (*w*, stretching C*sp*^{2}-H), 3109.3 (*w*, stretching C*sp*^{2}-H), 3047.5 (*w*, stretching C*sp*^{3}-H), 3009.0 (*w*, C*sp*^{3}-H), 2955.0 (*w*, n.a.), 2889.4 (*w*, n.a.), 2839.2 (*w*, n.a.), 1724.4 (*s*, stretching C=O ester), 1620.2 (*m*, stretching C=N), 1573.9 (*s*, n.a), 1481.3 (*s*, bending C-H methylene group), 1473.6 (*s*, bending C-H methyl group), 1435.0 (*s*, n.a.), 1415.8 (*s*, n.a.), 1354.0 (*s*, stretching C-N), 1284.6 (*s*, stretching Ar-C-O ester), 1257.6 (*s*, n.a.), 1203.6 (*s*, stretching C-O ester), 1114.9 (*s*, n.a.), 1110.9 (*s*, n.a.), 1087.9 (*s*, n.a.), 1068.6 (*s*, n.a.), 972.1 (*s*, n.a.), 937.4 (*s* n.a.), 871.8 (*s*, stretching C-N heterocycle), 848.7 (*s*, n.a.), 763.8 (*s*, n.a.), 740.7 (*s*, stretching C-H 1,2-disubstituted Ar), 659.7 (*s*, n.a.), 624.9 (*s*, n.a.), 559.4 (*m*, n.a.), 505.4 (*w*, n.a.), 432.1 (*w*, n.a.), 428.2 (*w*, n.a.).

Melting point = 249.6 – 250.4 ºC (not corrected)

HRMS (ESI) = calculated for $C_{19}H_{16}N_4O_4$: 364.1172, found: [M+1] 365.1248.

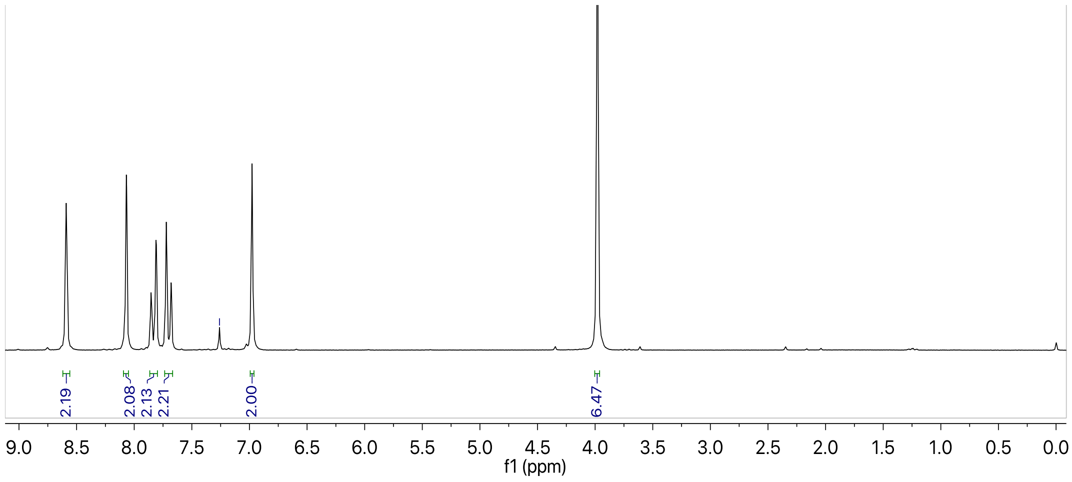

Figure S10. ^{1}H-NMR (400 MHz, $CDCl_3$, 298 K) spectrum of **L4**.

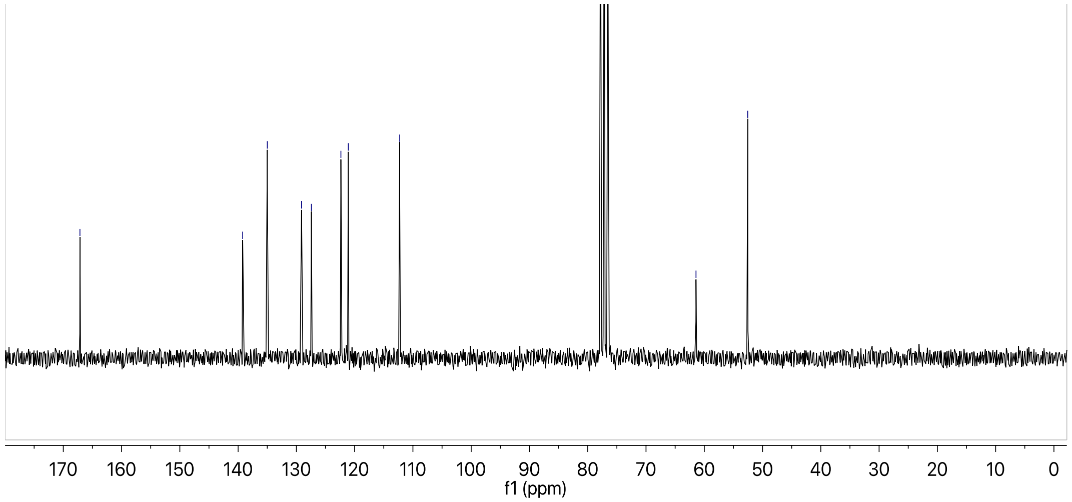

Figure S11. $^{13}C\{^{1}H\}$-NMR (101 MHz, $CDCl_3$, 298 K) spectrum of **L4**.

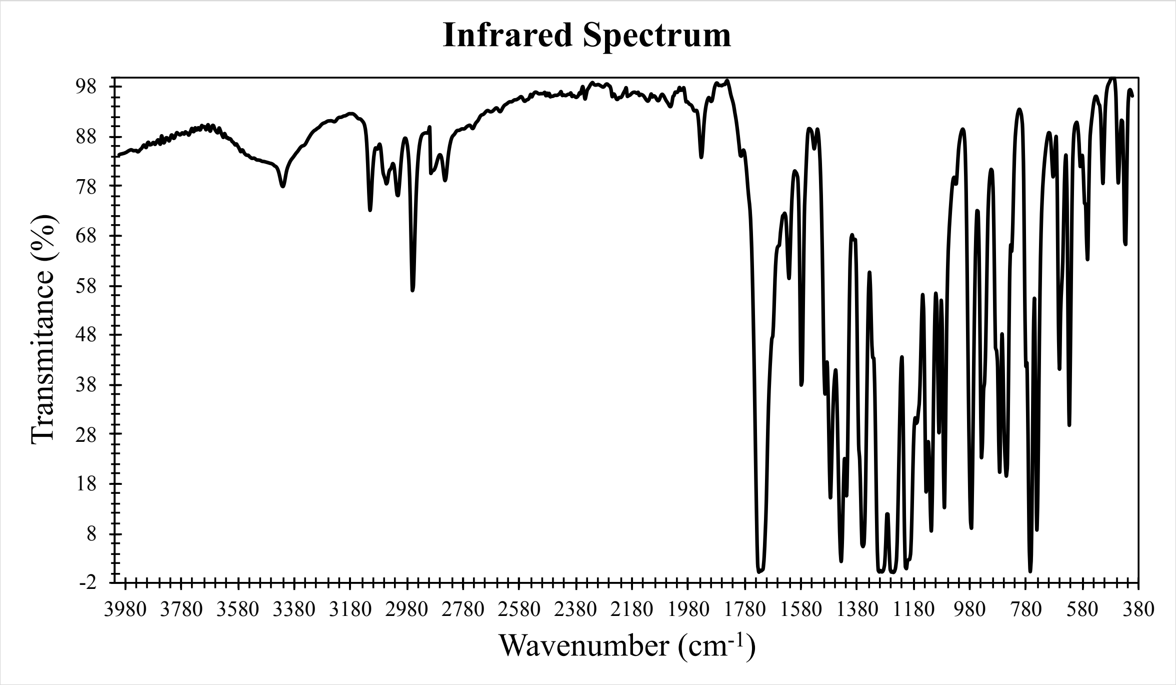

Figure S12. Infrared spectrum of **L4**.

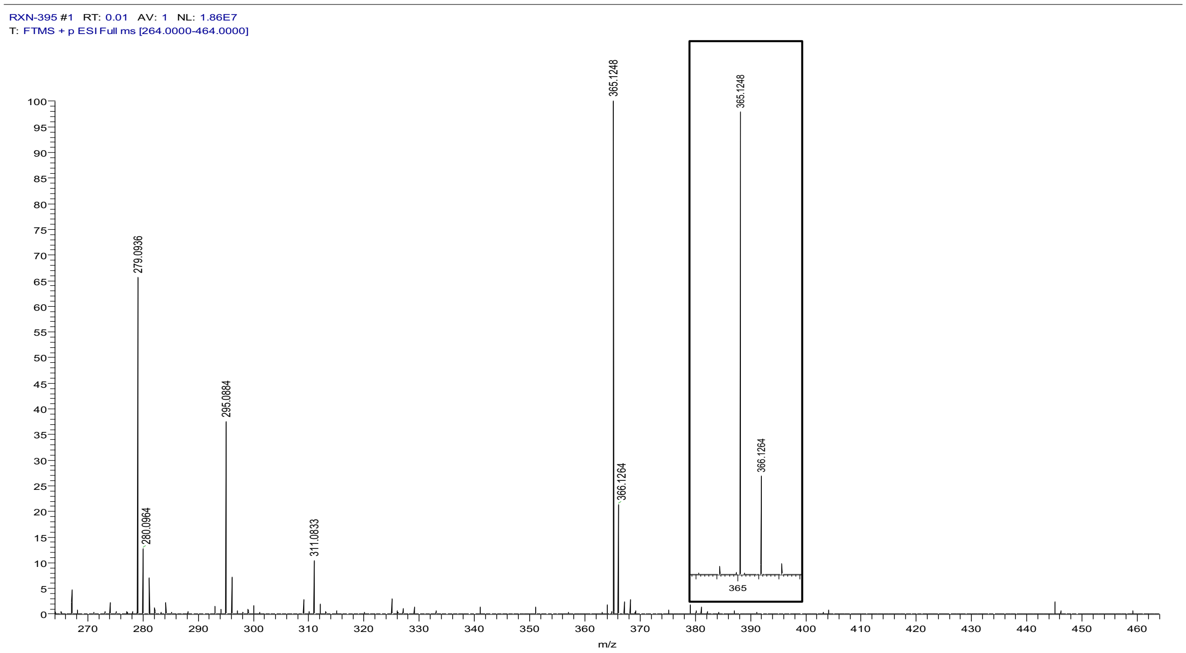

Figure S13. HRMS analysis of **L4** (positive ion mode).

1.3 General synthesis of heteroleptic Cu(I) complexes.

To the solution of the ligand (1 equiv.) in CH_2Cl_2/CH_3CN 1:1 (10 mL) was added $[Cu(CH_3CN)]BF_4$ (1 equiv.) in 1:1 molar ratio. The reaction mixture was stirred for 30 min, and after this time, a solution of the phosphine (1 equiv.) in CH_2Cl_2 (5 mL) was added and stirred for another 30 min. After this time, the mixture was evaporated. The crude is dissolved in CH_2Cl_2 and toluene is added, and the mixture is allowed to stand overnight at -20 ºC, where crystals appear. The crystals were filtered, washed with Et_2O, and dried in vacuo.

1.3.1 Compound C1

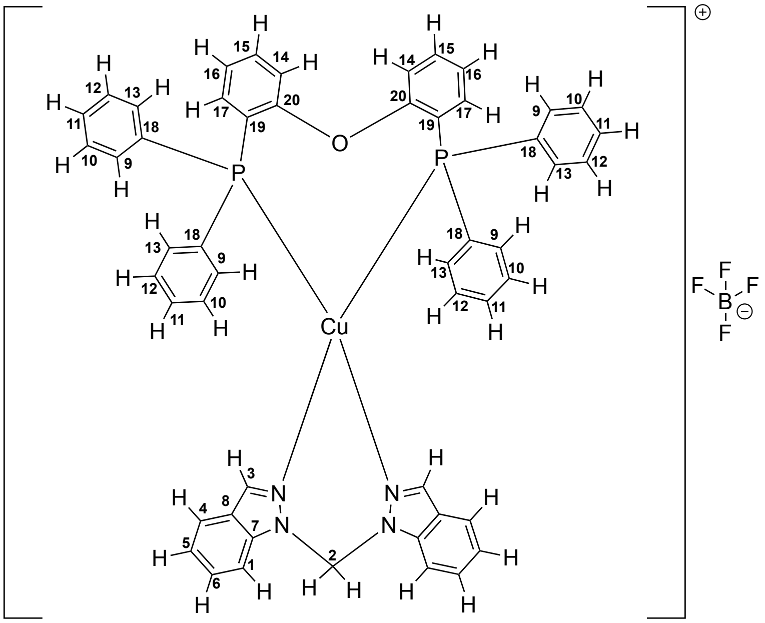

A yellow solid is obtained with a 76 % yield following the general procedure.

^{1}H-NMR (400 MHz, $CDCl_3$, 298 K) δ ppm = 8.26 (d, J = 8.5 Hz, 2H, H_1), 7.59 (t, J = 7.7 Hz, 2H, H_6), 7.37 (s, 2H, H_3), 7.32 (m, 2H, H_{16}), 7.26 (m, 12H, H_{10}, H_{11}, H_{12}), 7.18 (d, J = 8.0 Hz, 2H, H_{17}), 7.16 (dt, J = 8.1, 5.7 Hz, 8H, H_9, H_{13}), 7.15 (m, 2H, H_4), 7.13. (m, 2H, H_5), 7.07 (d, J = 5.9 Hz, 2H, H_2), 7.01 (t, J = 7.6 Hz, 2H, H_{15}), 6.83 (dtd, J = 7.9, 4.0, 1.6 Hz, 2H, H_{14}).

^{13}**C{^{1}H}-NMR** (101 MHz, $CDCl_3$, 298 K) δ ppm = 158.3 (t, J^{C-P} = 6.0 Hz, C_{19}), 139.9 (C_7), 138.0 (n.a.), 137.2 (C_3), 134.7 (C_{14}), 133.3 (t, J^{C-P} = 8.0 Hz, C_{17}), 132.1 (n.a.), 130.7 (t, J^{C-P} = 16.7 Hz, C_{18}), 130.5 (C_{10}, C_{12}), 129.8 (C_6), 129.1 (t, J^{C-P} = 4.7 Hz, C_9, C_{13}), 128.8 (d, J^{C-P} = 81.5 Hz, C_{11}), 125.4 (C_4), 125.2 (C_8), 124.4 (t, J^{C-P} = 14.3 Hz, C_{20}), 122.8 (C_5), 120.7 (C_{16}), 120.3 (C_{15}), 110.6 (C_1), 57.9 (C_2).

^{19}**F-NMR** (400 MHz, $CDCl_3$, 298 K) δ ppm = –152.15 (4 F, $^-BF_4$).

^{31}**P{^{1}H}-NMR** (160 MHz, $CDCl_3$, 298 K) δ ppm = –13,36 (2 P, DPEPhos).

^{11}**B{^{1}H}-NMR** (128 MHz, $CDCl_3$, 298 K) δ ppm = –0.57 (1 B, $^-BF_4$).

FT-IR (*w* weak, *m* medium, *s* strong) $\tilde{\nu}_{max}$ cm^{-1} = 3128.5 – 2989.7 (*w*, stretching Csp^2-H, Csp^3-H), 1620.2 (*m*, stretching C=N), 1589.3 (*m*, stretching C=C), 1566.2 (*m*, n.a.), 1504.5 (*m*,), 1462.0 (*s*, bending C-H methylene group), 1435.0 (*s*, n.a.), 1400.3 (*m*, n.a.), 1396.5 (*m*, stretching C-N), 1377.2 (*m*, stretching C-N), 1319.3 (*m*, n.a.), 1284.6 (*s*, n.a.), 1261.5 (*s*, stretching C-O Ar-O-Ar), 1215.2 (*s*, n.a.), 1180.4 (*m*, n.a.), 1157.3 (*m*, n.a.), 1057.0 (*s*, stretching P-C), 952.8 (*m*, n.a.), 910.4 (*m*, n.a.), 871.8 (*m*, stretching C-N heterocycle), 860.3 (*m*, n.a.), 802.4 (*m*, n.a.), 744.5 (*s*, stretching C-H 1,2-disubstituted Ar), 698.2 (*s*, n.a.), 644.2 (*w*, n.a.), 613.4 (*m*, n.a.), 590.2 (*w*, stretching Cu-N), 532.4 (*m*, stretching $^-BF_4$), 513.1 (*s*, n.a.), 482.2 (*s*, n.a.), 428.2 (*m*, n.a.).

Melting point = Not determined

HRMS (ESI) = calculated for $[C_{51}H_{40}CuN_4OP_2]^+$: 849.1968, found: [M+] 849.1958.

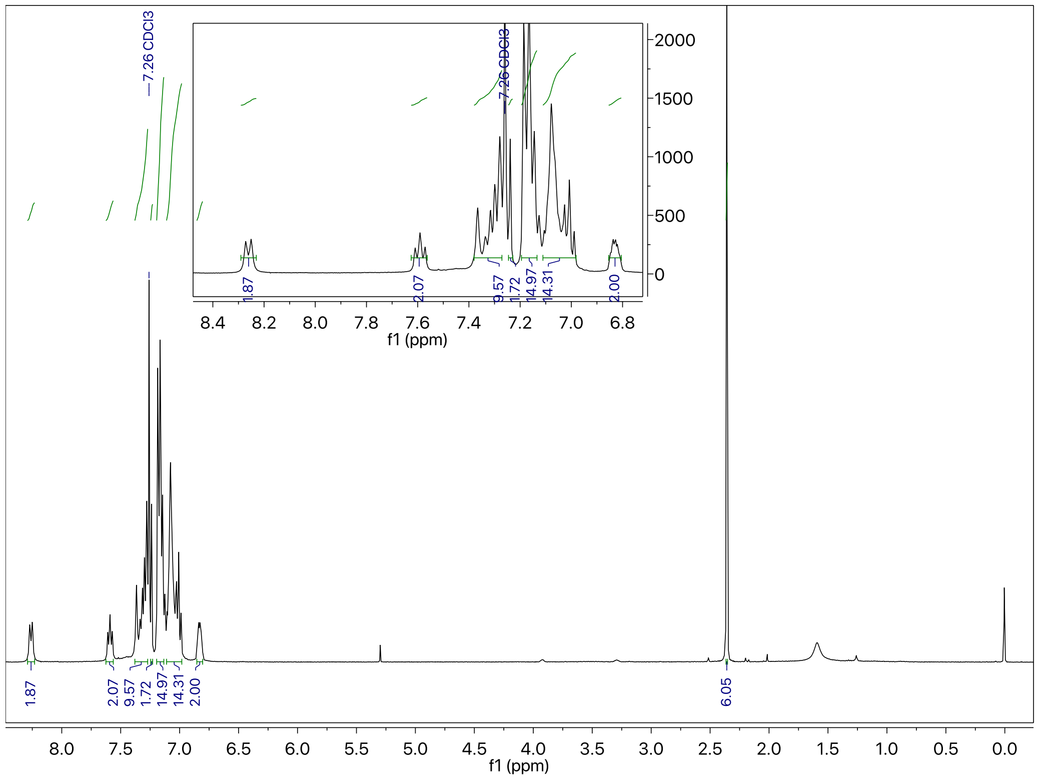

Figure S14. ^{1}H-NMR (400 MHz, $CDCl_3$, 298 K) spectrum of **C1**.

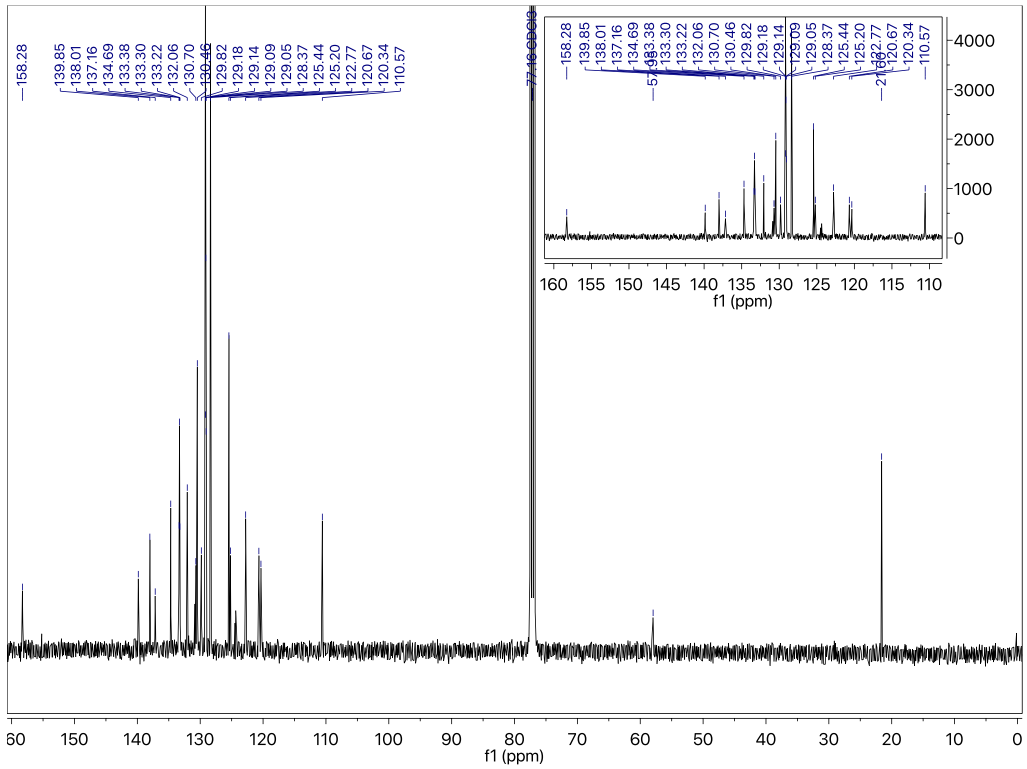

Figure S15. $^{13}C\{^{1}H\}$-NMR (101 MHz, $CDCl_3$, 298 K) spectrum of **C1**.

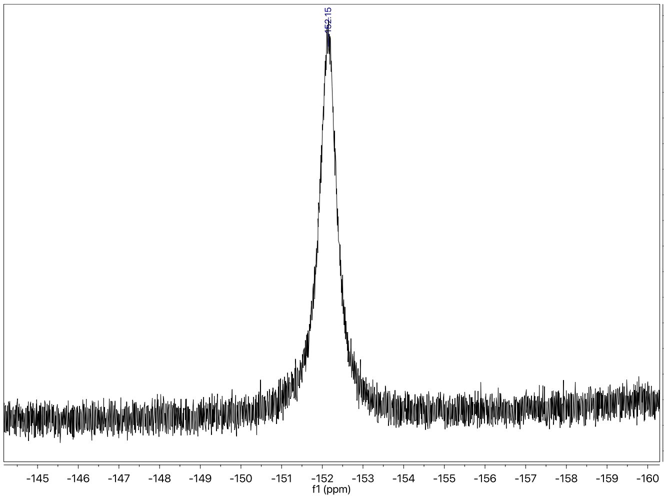

Figure S16. $^{19}F\{^{1}H\}$-NMR (400 MHz, $CDCl_3$, 298 K) spectrum of **C1**.

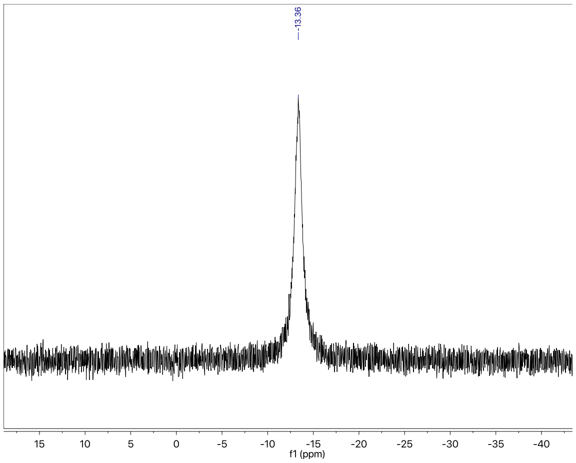

Figure S17. $^{31}P\{^{1}H\}$-NMR (160 MHz, $CDCl_3$, 298 K) spectrum of **C1**.

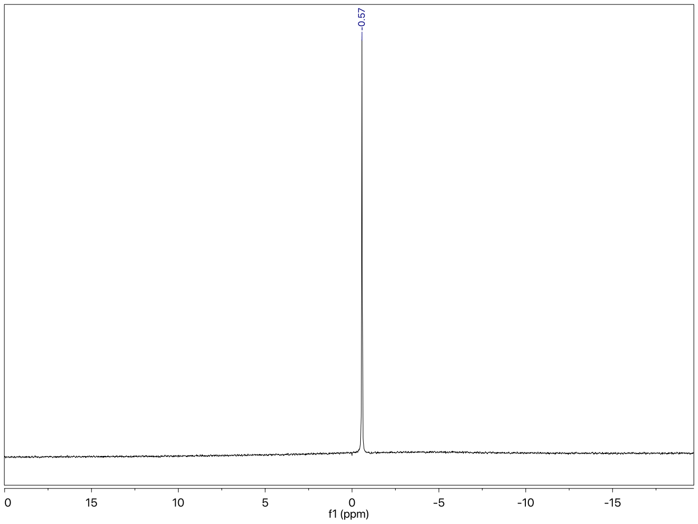

Figure S18. $^{11}B\{^{1}H\}$-NMR (128 MHz, $CDCl_3$, 298 K) spectrum of **C1**.

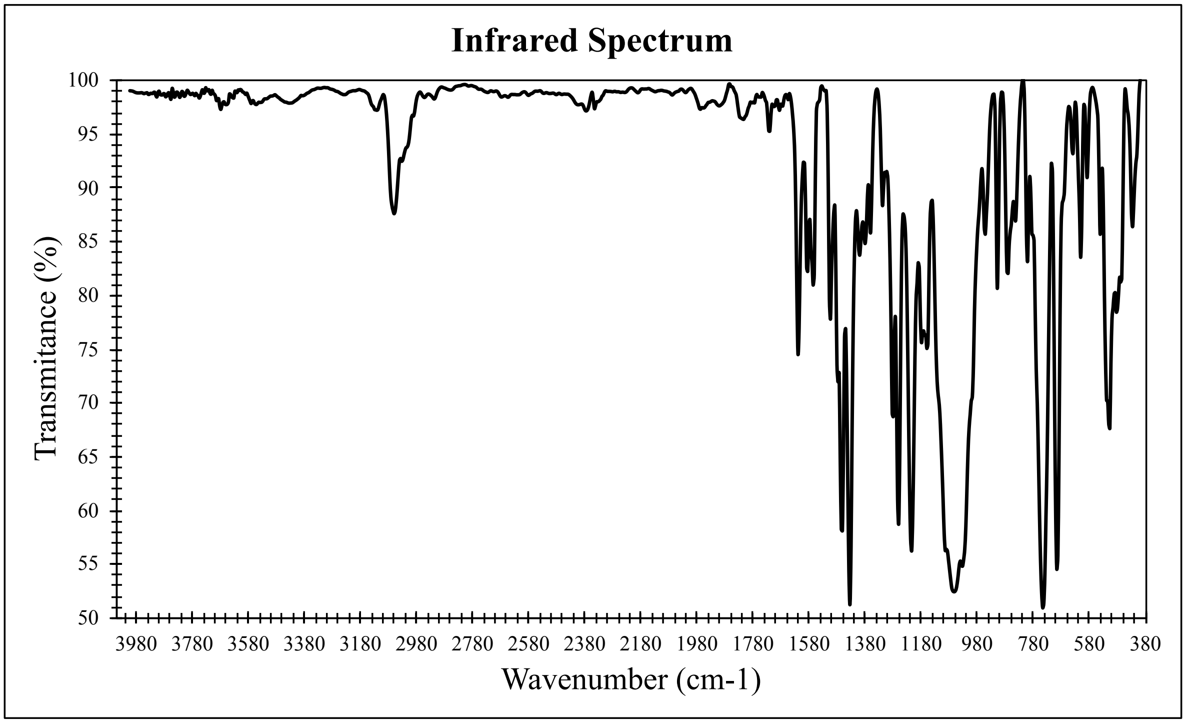

Figure S19. Infrared spectrum of **C1**.

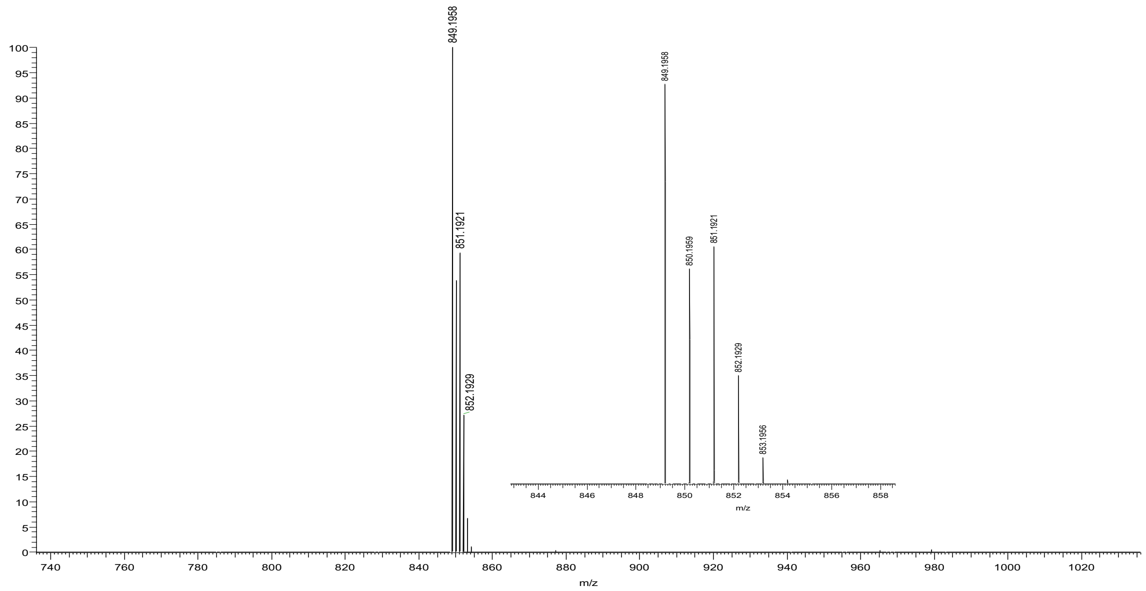

Figure S20. HRMS analysis of **C1** (positive ion mode).

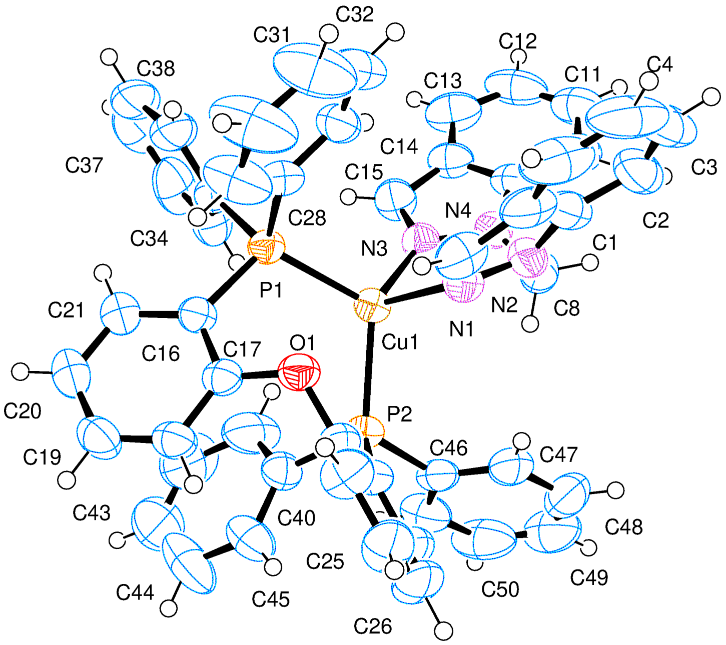

Figure S21. XRD molecular structure of **C1**.

Table S3. Selected crystallographic data of compound **C1**.

Bond distances			Selected Angles			
**Atom 1**	**Atom 2**	**Length (A)**	**Atom 1**	**Atom 2**	**Atom 3**	**Angle (º)**
Cu1	N1	2.140(5)	P1	Cu1	P2	114.23(5)
Cu1	N3	2.102(5)	P1	Cu1	N1	108.1(1)
N1	N2	1.353(7)	P1	Cu1	N3	110.1(1)
N2	C8	1.423(9)	P2	Cu1	N1	104.1(1)
N3	N4	1.352(7)	P2	Cu1	N3	124.8(1)
N4	C8	1.436(9)	N1	Cu1	N3	91.4(2)
			C17	O1	C23	117.2(4)
			Cu1	N1	N2	116.9(3)
			Cu1	N3	N4	119.1(3)

Selected Torsions				
**Atom 1**	**Atom 2**	**Atom 3**	**Atom 4**	**Torsion (º)**
P2	Cu1	N1	C7	-105.4(5)
P1	Cu1	N3	C15	-32.4(6)
N1	Cu1	N3	N4	22.1(4)
C17	O1	C23	C22	83.7(6)
N1	N2	C8	N4	72.1(7)
C15	N3	N4	C8	-177.4(5)

Table S4. Crystal data and structure refinement for **C1**.

Identification code	Ch81
Empirical formula	C51 H40 B Cu F4 N4 O P2
Formula weight	937.16
Temperature	294(2) K
Wavelength	0.71073 Å
Crystal system	Orthorhombic
Space group	P 21 21 21
Unit cell dimensions	a = 15.0928(18) Å α = 90º
	b = 16.164(2) Å β = 90º
	c = 20.550(3) Å γ = 90º
Volume	5013.4(11) Å^3
Z	4
Density (calculated)	1.242 Mg/m^3

Absorption coefficient	0.553 mm^{-1}
F(000)	1928
Crystal size	0.220 x 0.140 x 0.130 mm^3
Theta range for data collection	1.603 to 28.536°.
Index ranges	-20<=h<=20, -21<=k<=21, -27<=l<=27
Reflections collected	122726
Independent reflections	12635 [R(int) = 0.0638]
Completeness to theta = 25.242°	100.0 %
Absorption correction	Semi-empirical from equivalents
Max. and min. transmission	1 and 0.834
Refinement method	Full-matrix least-squares on F^2
Data / restraints / parameters	12635 / 6 / 577
Goodness-of-fit on F^2	1.100
Final R indices [I>2sigma(I)]	R1 = 0.0610, wR2 = 0.1748
R indices (all data)	R1 = 0.0845, wR2 = 0.1949
Absolute structure parameter	0.017(4)
Largest diff. peak and hole	0.955 and -0.357 e.$Å^{-3}$

1.3.2 Compound C2

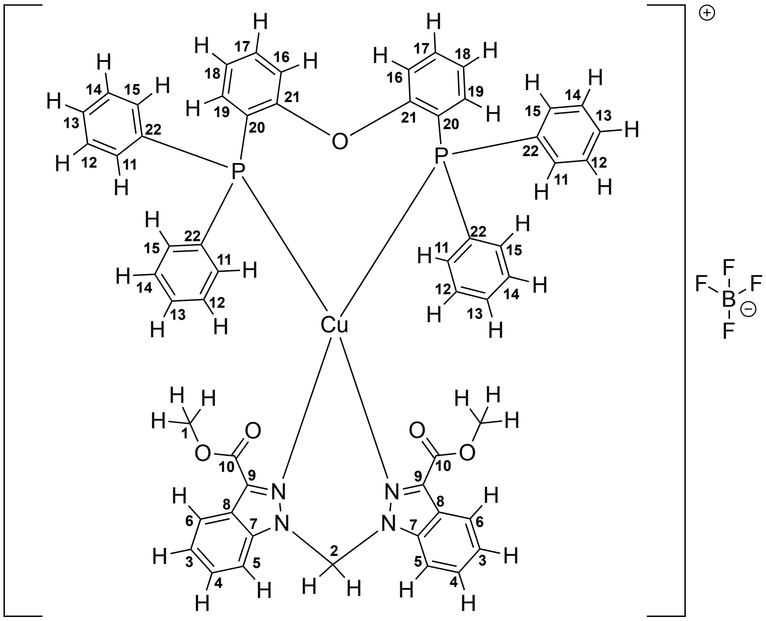

A white solid is obtained with an 81 % yield following the general procedure.

^{1}H-NMR (400 MHz, $CDCl_3$, 298 K) δ ppm = 8.03 (d, J = 8.1 Hz, 4H, H_5, H_6), 7.35 (q, J = 7.2 Hz, 12H, H_{12}, H_{13}, H_{14}), 7.23 (dd, J = 10.9, 4.3 Hz, 4H, H_3, H_4), 7.22 – 7.17 (m, 8H, H_{11}, H_{15}), 7.11 – 7.06 (m, 2H, H_{19}), 7.02 (t, J = 7.5 Hz, 2H, H_{17}), 6.88 – 6.85 (m, 2H, H_2), 6.82 (td, J = 7.8, 1.7 Hz, 2H, H_{18}), 6.68 (tdd, J = 8.5, 4.8, 2.6 Hz, 2H, H_{16}), 3.98 – 3.82 (m, 6H, H_1).

^{13}C{^{1}H}-NMR (101 MHz, $CDCl_3$, 298 K) δ ppm = 162.8 (C_{10}), 157.8 (t, J^{C-P} = 5.8 Hz, C_{20}), 140.6 (n.a.), 136.5 (n.a.), 134.4 (C_{16}), 133.6 (t, J^{C-P} = 8.1 Hz, C_{11}), 132.3 (C_{17}), 130.9 (C_{12}, C_{14}), 129.8 (C_{22}), 129.2 (t, J^{C-P} = 5.0 Hz, C_{15}), 129.1 (C_{19}), 128.6 (C_{13}), 128.3 (n.a.), 125.4 (C_{18}), 125.2 (n.a.), 124.4 (C_3, C_4), 123.7 (n.a.), 122.5 (t, J^{C-P} = 16.6 Hz, C_{21}), 122.3 (C_5, C_6), 120.0 (C_{16}), 117.8 (n.a.), 110.6 (n.a.), 63.1 (C_2), 52.5 (C_1).

^{19}F{^{1}H}-NMR (400 MHz, $CDCl_3$, 298 K) δ ppm = –153.02 (4F, $^{-}BF_4$).

^{31}P{^{1}H}-NMR (160 MHz, $CDCl_3$, 298 K) δ ppm = –14.42 (2P, DPEPhos).

^{11}B{^{1}H}-NMR (128 MHz, $CDCl_3$, 298 K) δ ppm = –0.72 (1B, $^{-}BF_4$)

FT-IR (*w* weak, *m* medium, *s* strong) $\tilde{\nu}_{max}$ cm^{-1} = 3055.2 (*w*, stretching C*sp*^{2}-H), 2993.5 (*w*, stretching C*sp*^{2}-H), 2947.2 (*w*, stretching C*sp*^{3}-H), 1720.5 (*s*, stretching C=O ester), 1678.1 (*s*, n.a.), 1616.4 (*s*, stretching C=N), 1570.1 (*s*, stretching C=C), 1566.2 (*s*, n.a.), 1481.3 (*s*, bending C-H methylene group), 1442.8 (*s*, bending C-H methyl group), 1438.9 (*s*, n.a.), 1411.9 (*s*, stretching C-N), 1334.7 (*s*, stretching C-N), 1292.3 (*s*, stretching Ar-C-O ester), 1257.6 (*s*, stretching C-O Ar-O-Ar), 1203.6 (*s*, stretching C-O ester), 1157.3 (*s*, n.a.), 1111.0 (*s*, n.a.), 1057.0 (*s*, stretching P-C), 941.3 (*s*, n.a.), 875.7 (*m*, stretching C-N heterocycle), 825.5 (*s*, n.a.), 775.4 (*m*,), 744.5 (*s*, stretching C-H 1,2-disubstituted Ar), 694.4 (*s*, n.a.), 621.1 (*s*, n.a.), 582.5 (*w*, stretching Cu-N), 532.4 (*w*, stretching $^-BF_4$), 513.1 (*s*, n.a.), 428.2 (*w*, n.a.).

Melting point = Not determined

HRMS (ESI) = calculated for $[C_{55}H_{44}CuN_4O_5P_2]^+$: 965.2077, found: [M+] 965.2049.

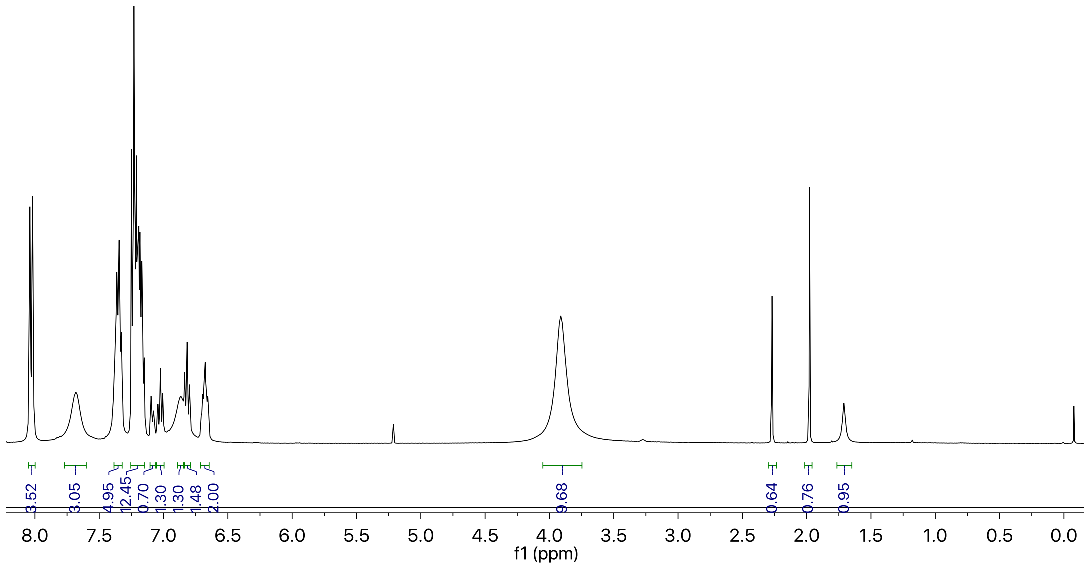

Figure S22. ^{1}H-NMR (400 MHz, $CDCl_3$, 298 K) spectrum of **C2**.

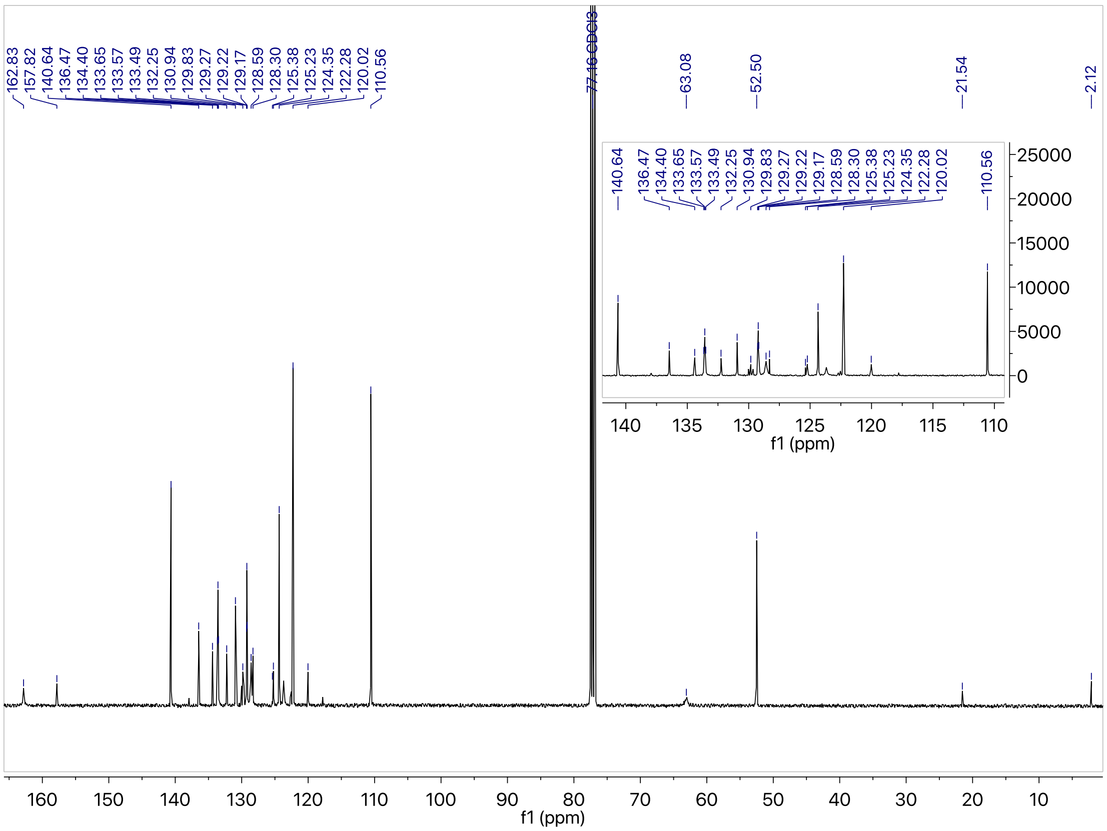

Figure S23. $^{13}C\{^1H\}$-NMR (101 MHz, $CDCl_3$, 298 K) spectrum of **C2**.

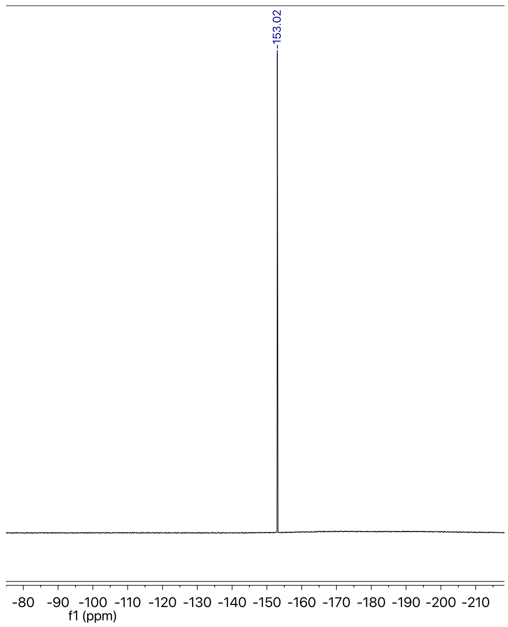

Figure S24. $^{19}F\{^1H\}$-NMR (400 MHz, $CDCl_3$, 298 K) spectrum of **C2**.

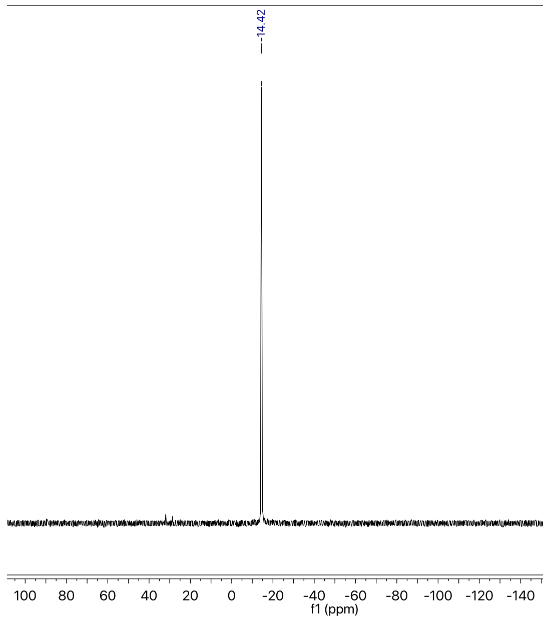

Figure S25. $^{31}P\{^{1}H\}$-NMR (160 MHz, $CDCl_3$, 298 K) spectrum of **C2**.

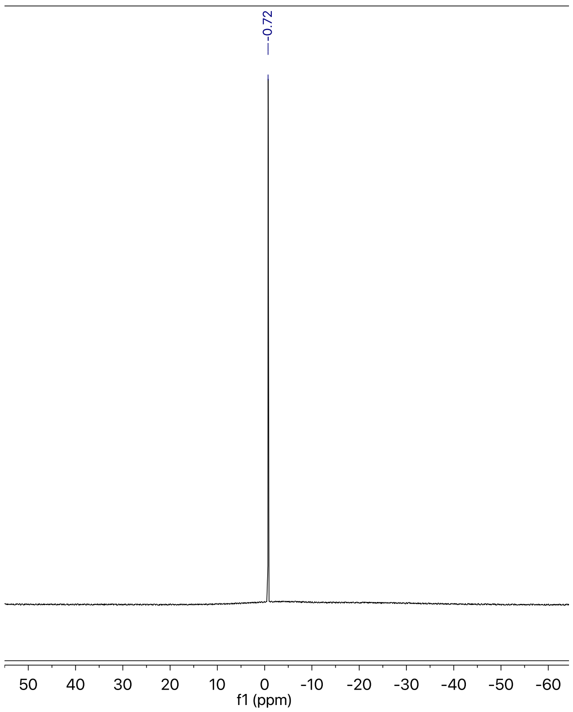

Figure S26. $^{11}B\{^{1}H\}$-NMR (128 MHz, $CDCl_3$, 298 K) spectrum of **C2**.

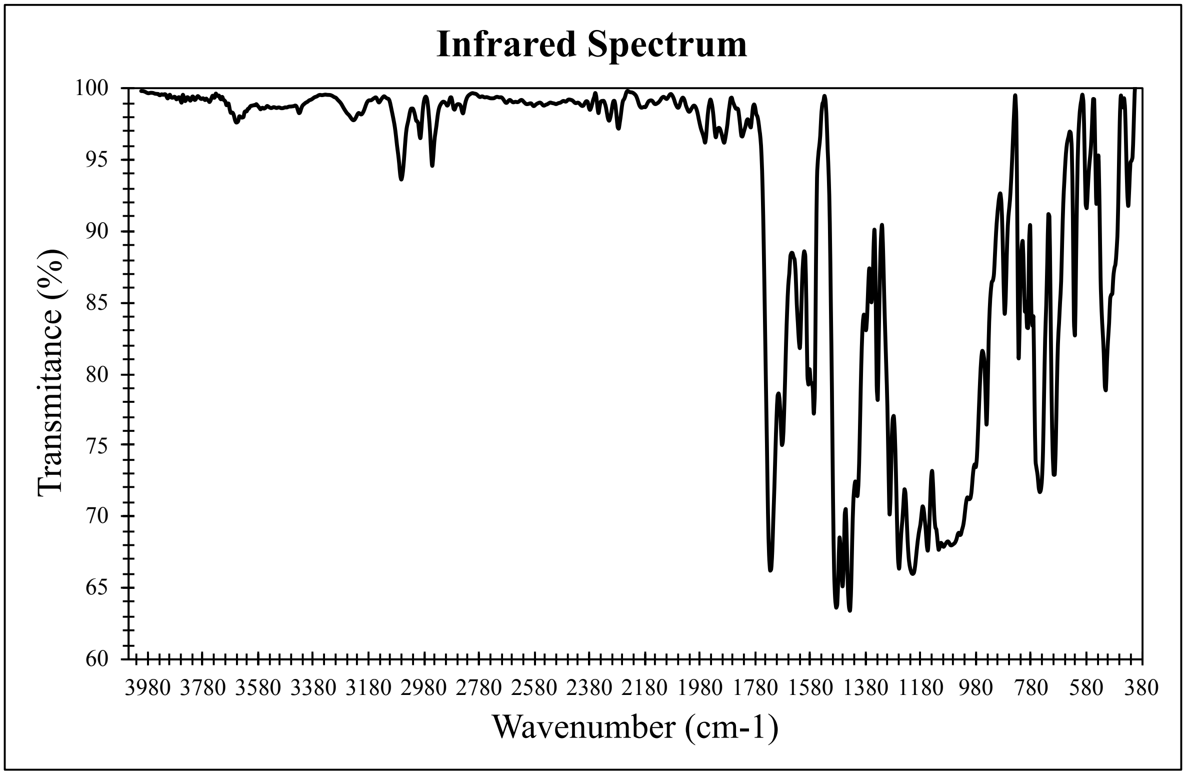

Figure S27. Infrared spectrum of **C2**.

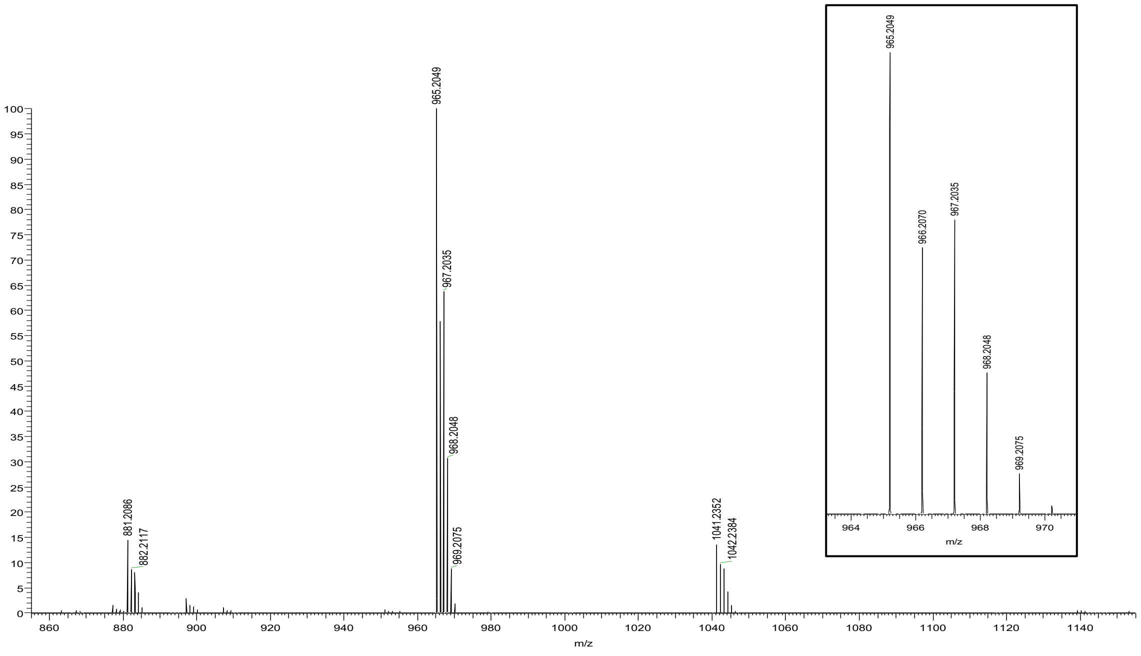

Figure S28. HRMS analysis of **C2** (positive ion mode).

1.3.3 Compound C3

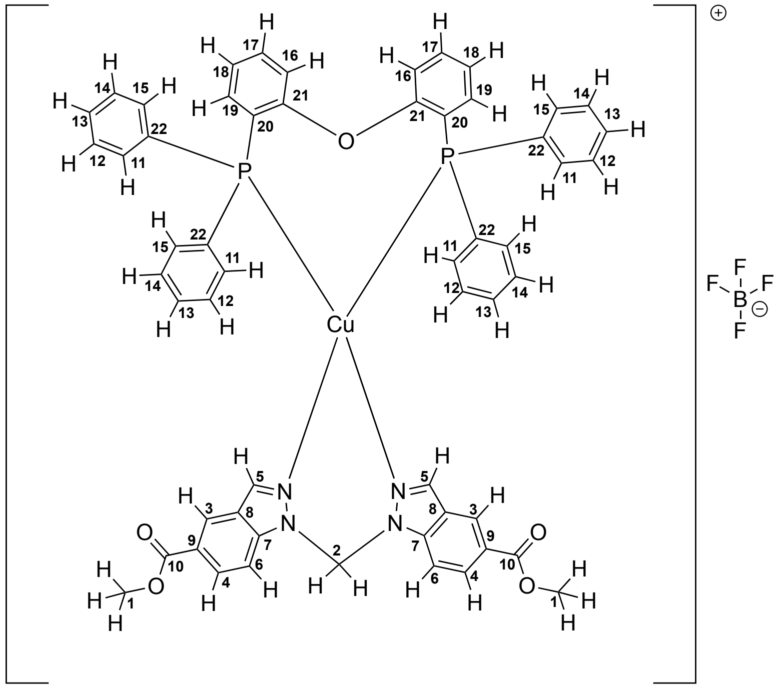

A pink solid is obtained with an 85 % yield following the general procedure.

¹H-NMR (400 MHz, $CDCl_3$, 298 K) δ ppm = 8.84 (s, 2H, H_3), 7.83 (d, J = 8.5 Hz, 2H, H_4), 7.51 (s, 2H, H_5), 7.44 (d, J = 8.5 Hz, 2H, H_6), 7.30 (d, J = 8.1 Hz, 2H, H_{18}), 7.25 (dd, J = 10.3, 6.9 Hz, 2H, H_{19}), 7.16 (t, J = 7.0 Hz, 12H, H_{12}, H_{13}, H_{14}), 7.09 (s, 2H, H_2), 7.08 – 7.04 (*m*, 8H, H_{11}, H_{15}), 7.00 (d, J = 7.8 Hz, 2H, H_{17}), 6.81 (p, J = 4.0 Hz, 2H, H_{16}), 4.03 (s, 6H, H_1).

¹³C{¹H}-NMR (101 MHz, $CDCl_3$, 298 K) δ ppm = 166.8 (C_{10}), 158.2 (t, J^{C-P} = 6.0 Hz, C_{20}), 139.3 (C_8), 138.0 (n.a), 137.6 (C_5), 134.7 (C_{16}), 133.2 (t, J^{C-P} = 8.0 Hz, C_{11}, C_{15}), 132.3 (C_{18}), 131.5 (C_7), 130.6 (C_{19}), 130.3 (t, J^{C-P} = 17.2 Hz, C_{22}), 129.2 – 129.1 (m, C_{12}, C_{13}, C_{14}), 128.3 (n.a.), 125.4 (d, J^{C-P} = 8.2 Hz, C_{17}), 123.8 (t, J^{C-P} = 14.8 Hz, C_{21}), 123.4 (C_4), 121.3 (C_6), 120.3 (n.a.), 112.1 (C_3), 58.4 (C_2), 52.8 (C_1).

¹⁹F{¹H}-NMR (400 MHz, $CDCl_3$, 298 K) δ ppm = –152.52 (4F, $^-BF_4$).

³¹P{¹H}-NMR (160 MHz, $CDCl_3$, 298 K) δ ppm = –12.93 (2P, DPEPhos).

^{11}B{^{1}H}-NMR (128 MHz, $CDCl_3$, 298 K) δ ppm = –0.67 (1B, $^{-}BF_4$)

FT-IR (*w* weak, *m* medium, *s* strong) $\tilde{\nu}_{max}$ cm^{-1} = 3120.8 (*w*, stretching C*sp*^{2}-H), 3059.1 (*w*, stretching C*sp*^{2}-H), 2993.5 (*w*, stretching C*sp*^{3}-H), 2951.1 (*w*, stretching C*sp*^{3}-H), 1716.7 (*s*, stretching C=O ester), 1620.2. (*s*, stretching C=N), 1570.1 (*m*, stretching C=C), 1566.2 (*m*, n.a.), 1558.4 (*m*, n.a.), 1508.3 (*m*, n.a.), 1446.6 (*s*, bending C-H methylene group), 1435.0 (*s*, bending C-H methyl group), 1377.2 (*s*, stretching C-N), 1369.5 (*s*, stretching C-N), 1354.0 (*s*, n.a.), 1315.5 (*s*, stretching Ar-C-O ester), 1265.3 (*s*, stretching C-O Ar-O-Ar), 1249.9 (*s*, n.a.), 1195.9 (*s*, stretching C-O ester), 1091.7 (*s*, n.a.), 1072.4 (*s*, stretching P-C), 987.6 (*m*, n.a.), 933.6 (*m*, n.a.), 864.1 (*m*, stretching C-N heterocycle), 844.8 (*m*, n.a.), 837.1 (*m*, n.a.), 787.0 (*m*, n.a.), 763.8 (*s*, stretching C-H 1,2-disubstituted Ar), 694.4(*s*, n.a.), 632.7 (*m*, n.a.), 624.9 (*m*, n.a.), 578.6 (*w*, stretching Cu-N), 509.2 (*m*, stretching $^{-}BF_4$), 435.9 (*m*, n.a.).

Melting point = Not determined

HRMS (ESI) = calculated for $[C_{55}H_{44}CuN_4O_5P_2]^{+}$: 965.2077, found: [M+] 965.2061.

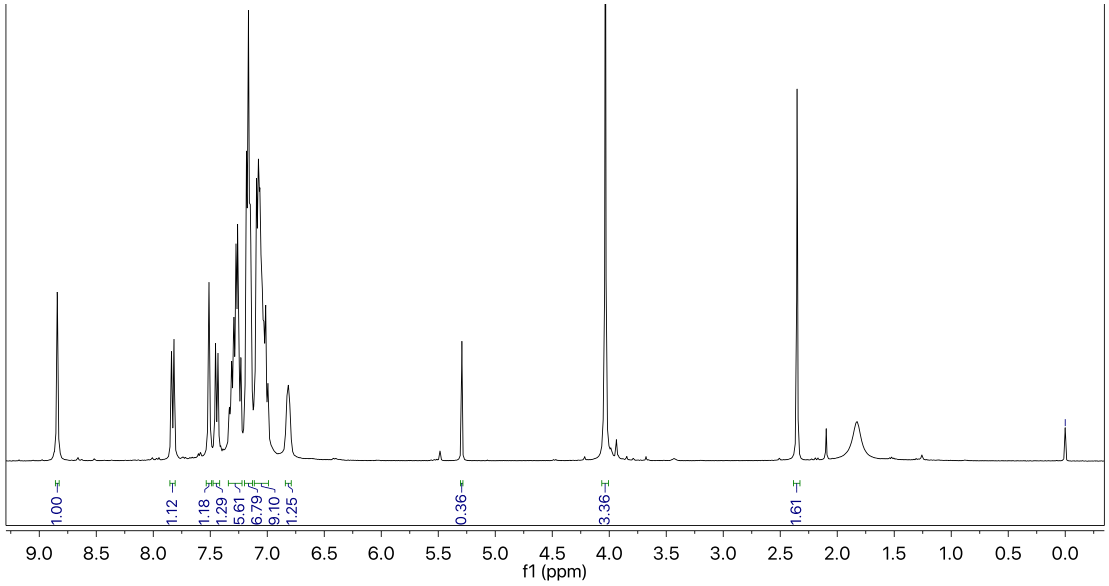

Figure S29. ^{1}H-NMR (400 MHz, $CDCl_3$, 298 K) spectrum of **C3**.

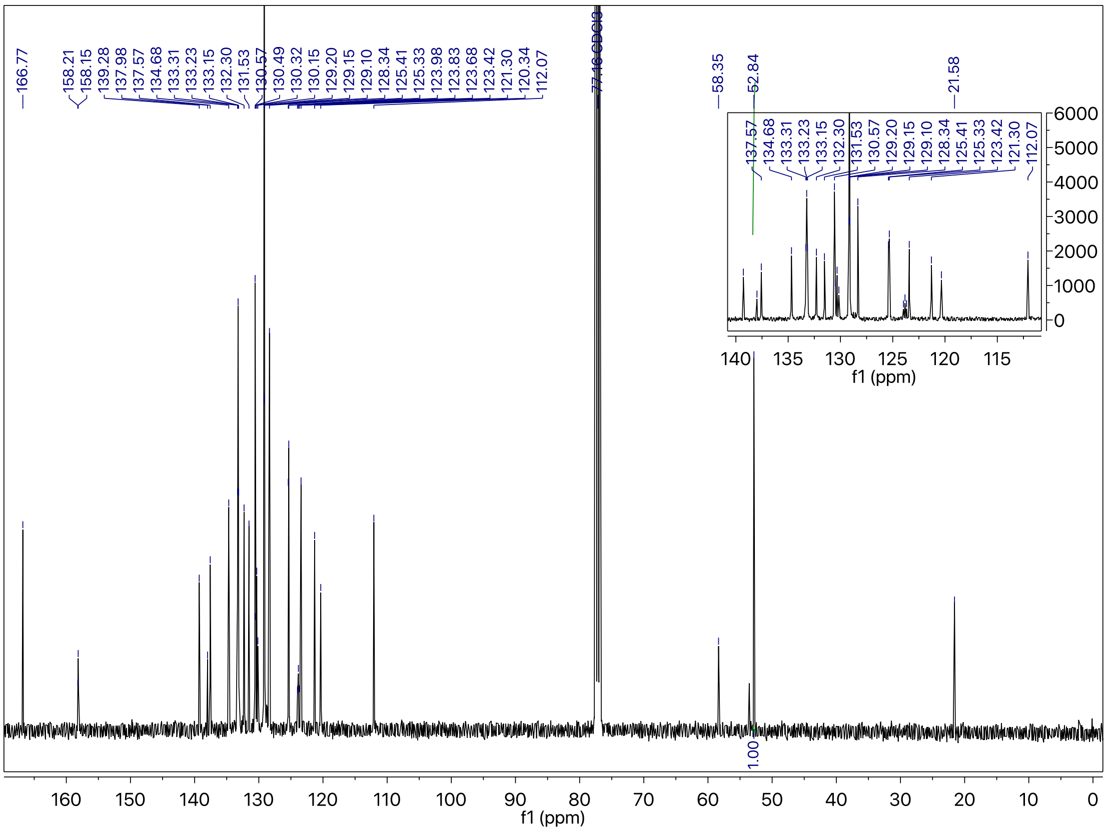

Figure S30. $^{13}C\{^{1}H\}$-NMR (101 MHz, $CDCl_3$, 298 K) spectrum of **C3**.

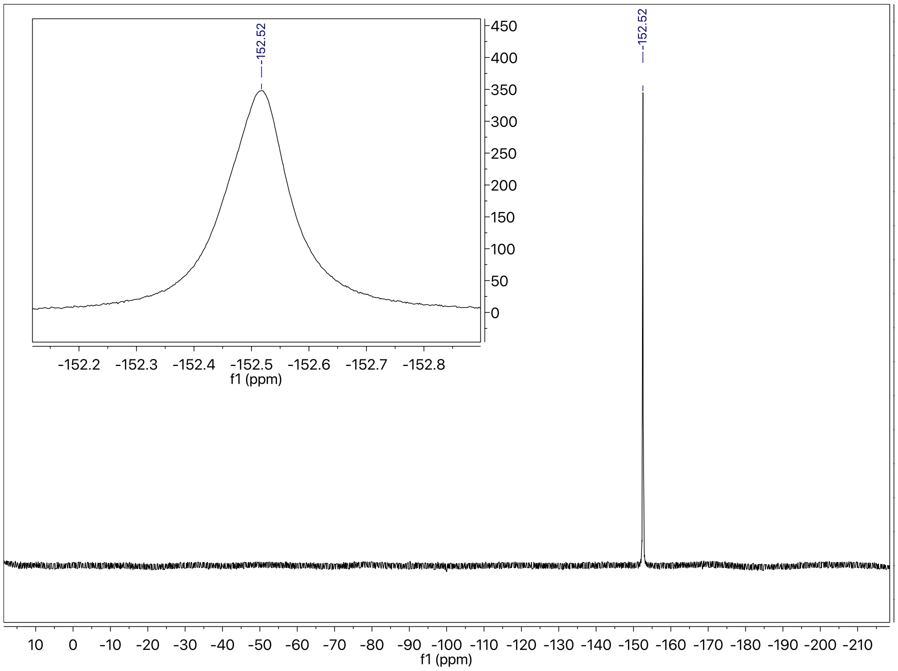

Figure S31. $^{19}F\{^{1}H\}$-NMR (400 MHz, $CDCl_3$, 298 K) spectrum of **C3**.

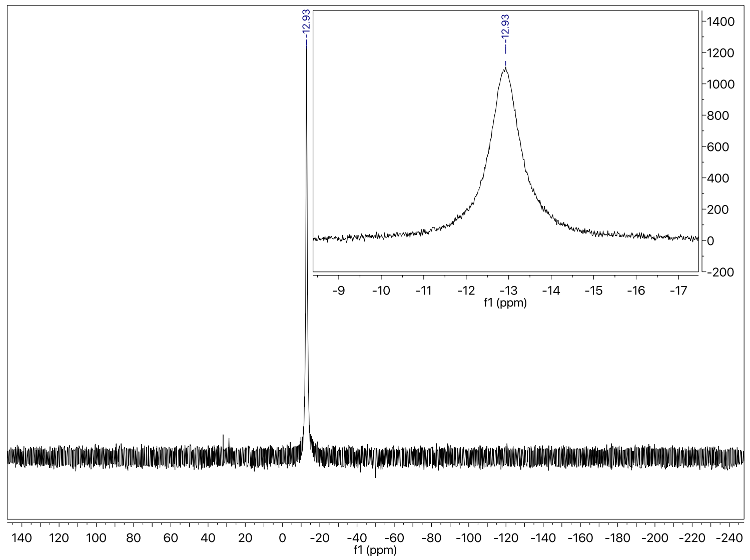

Figure S32. $^{31}P\{^1H\}$-NMR (160 MHz, $CDCl_3$, 298 K) spectrum of **C3**.

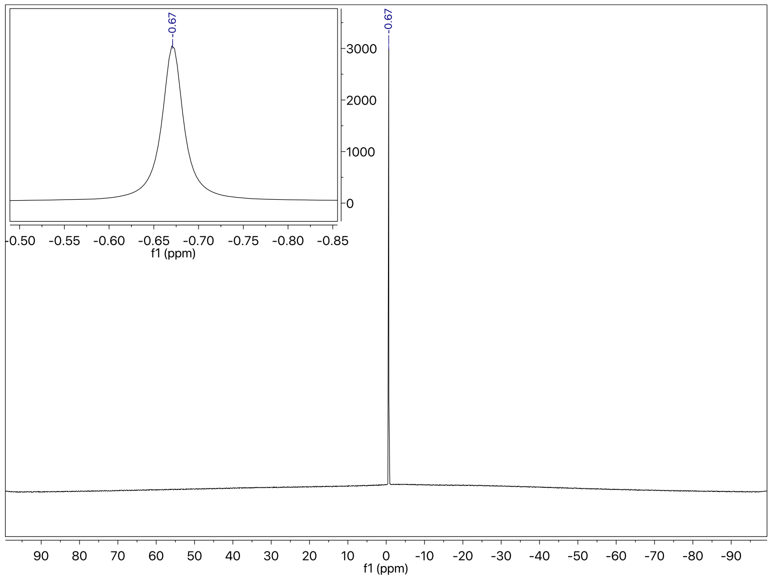

Figure S33. $^{11}B\{^1H\}$-NMR (128 MHz, $CDCl_3$, 298 K) spectrum of **C3**.

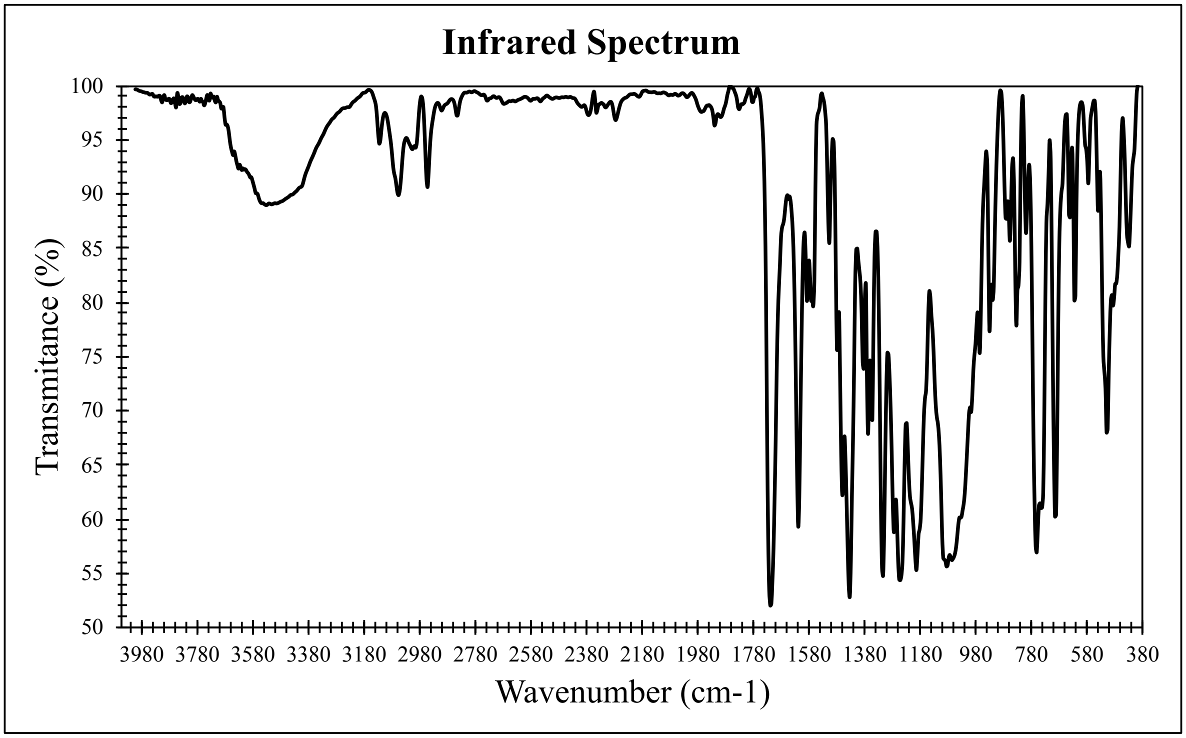

Figure S34. Infrared spectrum of **C3**.

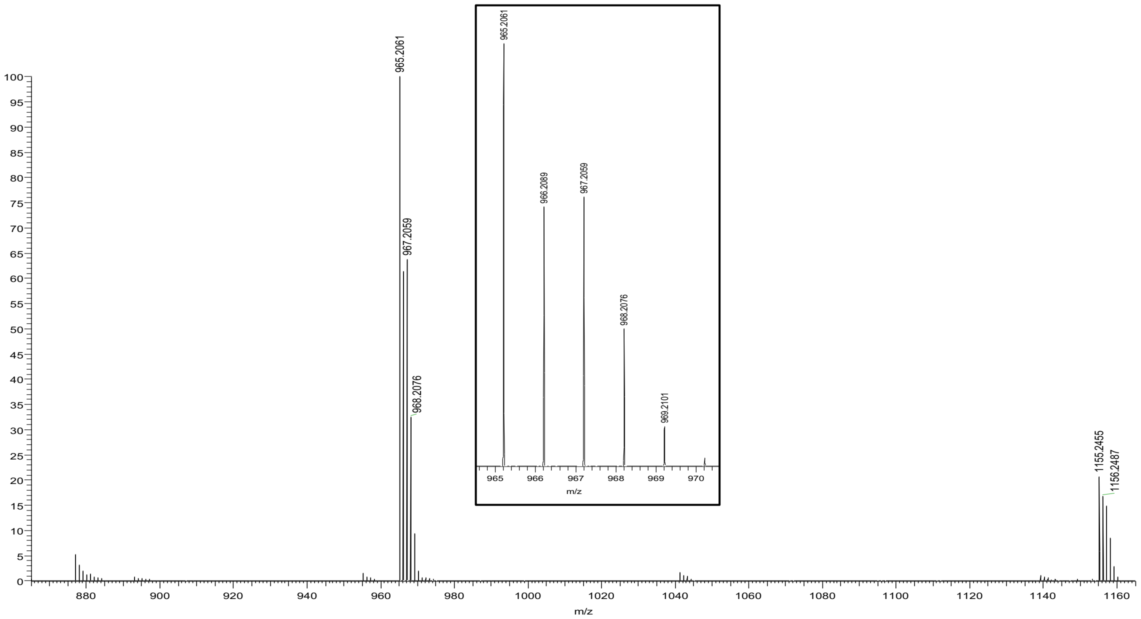

Figure S35. HRMS analysis of **C3** (positive mode ion).

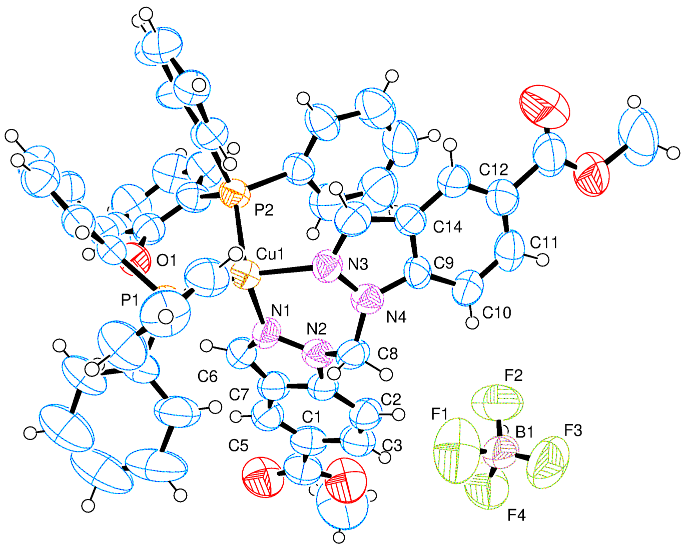

Figure S36. XRD molecular structure of **C3**.

Table S5. Selected crystallographic data of compound **C3**.

**Bond distances**		
**Atom 1**	**Atom 2**	**Length (A)**
Cu1	P1	2,237(2)
Cu1	P2	2,295(3)
Cu1	N1	2,042(7)
Cu1	N3	2,097(6)
O1	C37	1,398(9)
O1	C38	1,404(9)
O2	C16	1,20(1)
O3	C16	1,32(1)
O4	C18	1,19(2)
O5	C18	1,29(2)
N1	N2	1,37(1)
N2	C8	1,44(1)
N3	N4	1,38(1)
N4	C8	1,43(1)

**Selected Angles**			
**Atom 1**	**Atom 2**	**Atom 3**	**Angle (º)**
P1	Cu1	P2	112,32(8)
P1	Cu1	N1	110,4(2)
P1	Cu1	N3	127,1(2)
P2	Cu1	N1	109,0(2)
P2	Cu1	N3	101,7(2)
N1	Cu1	N3	94,2(2)
C37	O1	C38	116,0(6)
N2	C8	N4	112,0(6)
O2	C16	O3	125(1)
O4	C18	O5	126(1)

**Selected Torsions**				
**Atom 1**	**Atom 2**	**Atom 3**	**Atom 4**	**Torsion (º)**
P1	Cu1	N1	N2	-128.2(5)
P2	Cu1	N1	N2	108.0(5)
N1	Cu1	N3	N4	1.0(5)
C38	O1	C37	C36	32(1)
N1	N2	C8	N4	-66.6(8)
C5	C4	C16	O2	-13(2)
C13	C12	C18	O4	-3(2)

Table S6. Crystal data and structure refinement for **C3**.

Identification code	Ch87
Empirical formula	C58.50 H47.50 B Cu F4 N4 O5.25 P2
Formula weight	1102.79
Temperature	295(2) K
Wavelength	0.71073 Å
Crystal system	Triclinic
Space group	P -1
Unit cell dimensions	a = 12.248(10) Å α = 104.848(16)°
	b = 13.934(12) Å β = 102.281(17)°
	c = 16.871(14) Å γ = 94.511(17)°
Volume	2692(4) $Å^3$
Z	2
Density (calculated)	1.360 Mg/m^3
Absorption coefficient	0.532 mm^{-1}
F(000)	1137
Crystal size	0.270 x 0.200 x 0.100 mm^3
Theta range for data collection	1.527 to 30.659°.
Index ranges	-16<=h<=17, -19<=k<=18, -22<=l<=23
Reflections collected	79473
Independent reflections	14794 [R(int) = 0.3967]
Completeness to theta = 25.242°	100.0 %
Refinement method	Full-matrix least-squares on F^2
Data / restraints / parameters	14794 / 51 / 697
Goodness-of-fit on F^2	0.984
Final R indices [I>2sigma(I)]	R1 = 0.1127, wR2 = 0.2205
R indices (all data)	R1 = 0.3314, wR2 = 0.3147
Largest diff. peak and hole	0.518 and -0.487 e.$Å^{-3}$

1.3.4 Compound C4

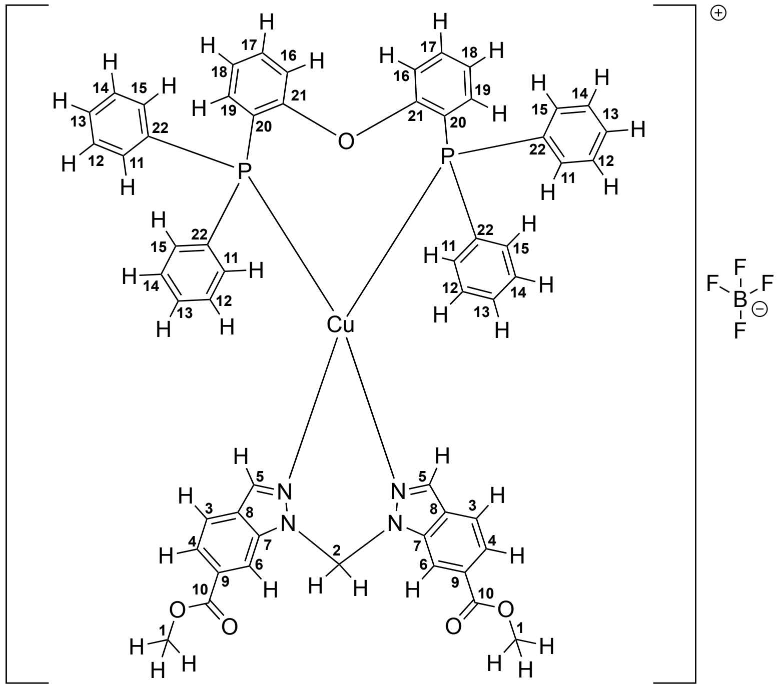

A white solid is obtained with an 87 % yield following the general procedure.

^{1}H-NMR (400 MHz, $CDCl_3$, 298 K) δ ppm = 8.82 (s, 2H, H_6), 7.83 (dd, *J* = 8.5, 1.1 Hz, 2H, H_4), 7.55 (s, 2H, H_5), 7.47 (d, *J* = 8.5 Hz, 2H, H_3), 7.30 (dq, *J* = 8.0, 3.1, 2.6 Hz, 2H, H_{18}), 7.22 (t, *J* = 7.5 Hz, 12H, H_{12}, H_{13}, H_{14}), 7.15 – 7.10 (m, 8H, H_{11}, H_{15}), 7.13 (m, 2H, H_{19}), 7.08 (*s*, 2H, H_2), 7.00 (t, *J* = 7.6 Hz, 2H, H_{17}), 6.79 (dtd, J = 8.0, 4.2, 1.6 Hz, 2H, H_{16}), 4.02 (s, 6H, H_1).

^{13}C{^{1}H}-NMR (101 MHz, $CDCl_3$, 298 K) δ ppm = 166.7 (C_{10}), 158.1 (t, $J^{C\text{-}P}$ = 6.0 Hz, C_{20}), 139.2 (C_7), 137.5 (C_5), 134.6 (C_{16}), 133.3 (t, $J^{C\text{-}P}$ = 8.0 Hz, C_{11}, C_{15}), 132.2 (C_{12}, C_{14}), 131.4 (C_8), 130.5 (C_{18}), 130.4 (t, $J^{C\text{-}P}$ = 17.2 Hz, C_{22}), 129.1 (t, $J^{C\text{-}P}$ = 4.8 Hz, C_{19}), 125.4 (C_{13}), 123.7 (t, $J^{C\text{-}P}$= 14.9 Hz, C_{21}), 123.3 (C_4), 122.8 (d, $J^{C\text{-}P}$ = 497.9 Hz, C_{17}), 121.3 (C_3), 117.7 (C_9), 112.0 (C_6), 58.5 (C_2), 52.8 (C_1).

^{19}F{^{1}H}-NMR (400 MHz, $CDCl_3$, 298 K) δ ppm = -153.0 (4F, $^{-}BF_4$)

^{31}P{^{1}H}-NMR (160 MHz, $CDCl_3$, 298 K) δ ppm = –13,19 (2P, DPEPhos)

$^{11}B\{^1H\}$-NMR (128 MHz, $CDCl_3$, 298 K) δ ppm = –0.73 (1B, $^-BF_4$)

FT-IR (*w* weak, *m* medium, *s* strong) $\tilde{\nu}_{max}$ cm^{-1} = 3055.3 (*w*, stretching C*sp*^{2}-H), 3005.1 (*w*, stretching C*sp*^{2}-H), 2955.0 (*w*, stretching C*sp*^{3}-H), 1720.5 (*s*, stretching C=O ester), 1620.2 (*m*, stretching C=N), 1573.9 (*s*, stretching C=C), 1462.0 (*s*, bending C-H methylene group), 1435.0 (*s*, bending C-H methyl group), 1357.9 (*s*, stretching C-N), 1300 (*s*, stretching Ar-C-O ester), 1288.5 (*s*, stretching C-O Ar-O-Ar), 1253.7 (*s*, n.a.), 1207.4 (*s*, stretching C-O ester), 1084.0 (*s*, stretching P-C), 1064.7 (*s*, n.a.), 972.1 (*s*, n.a.), 937.4 (*m*, n.a.), 871.8 (*s*, stretching C-N heterocycle), 848.7 (*s*, n.a.), 802.4 (*m*, n.a.), 763.8 (*s*, stretching C-H 1,2-disubstituted Ar), 740.7 (*s*, n.a.), 698.2 (*s*, n.a.), 659.7 (*m*, n.a.), 624.9 (*m*, stretching Cu-N), 513.1 (*s*, stretching $^-BF_4$), 424.3 (*m*, n.a.).

Melting point = Not determined

HRMS (ESI) = calculated for $[C_{55}H_{44}CuN_4O_5P_2]^+$: 965.2077, found: [M+] 965.2044.

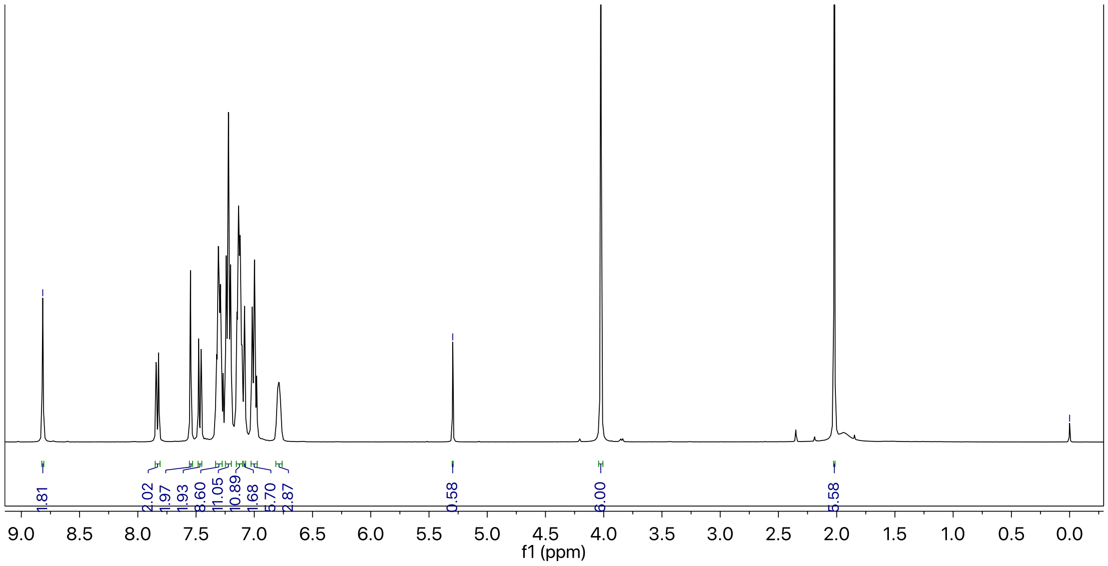

Figure S37. ^{1}H-NMR (400 MHz, $CDCl_3$, 298 K) spectrum of **C4**.

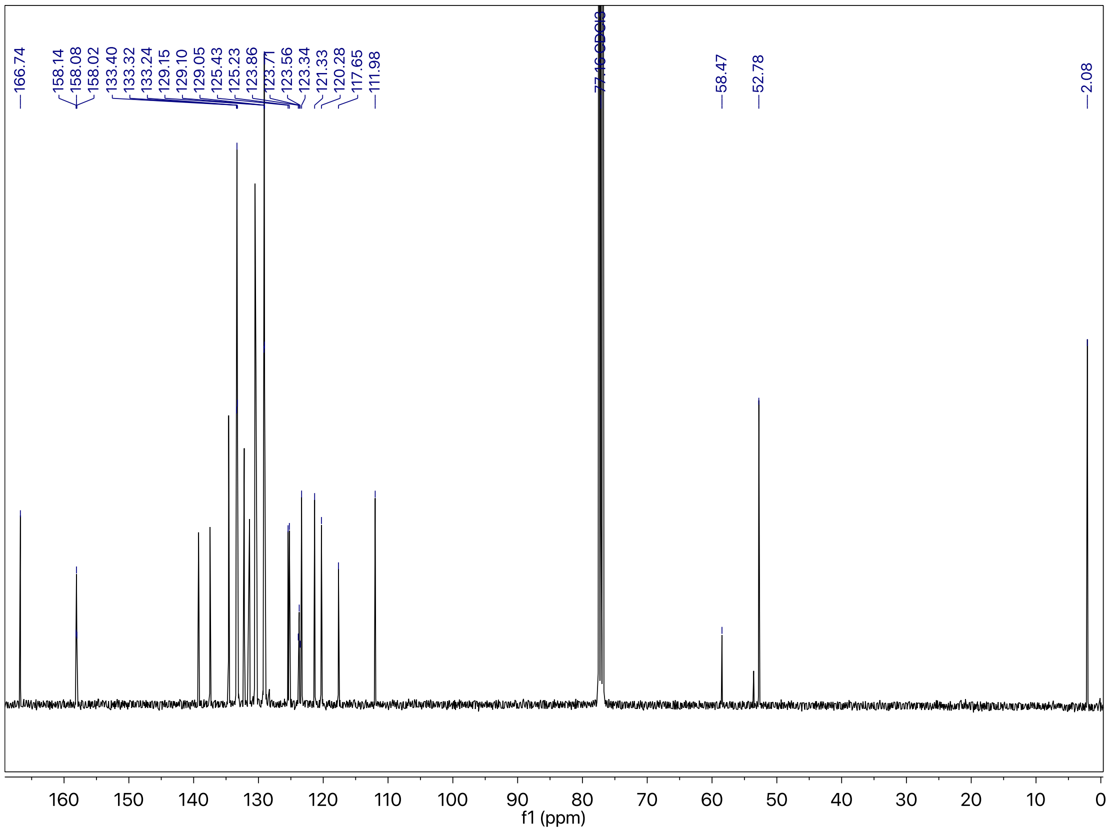

Figure S38. $^{13}C\{^{1}H\}$-NMR (101 MHz, $CDCl_3$, 298 K) spectrum of **C4**.

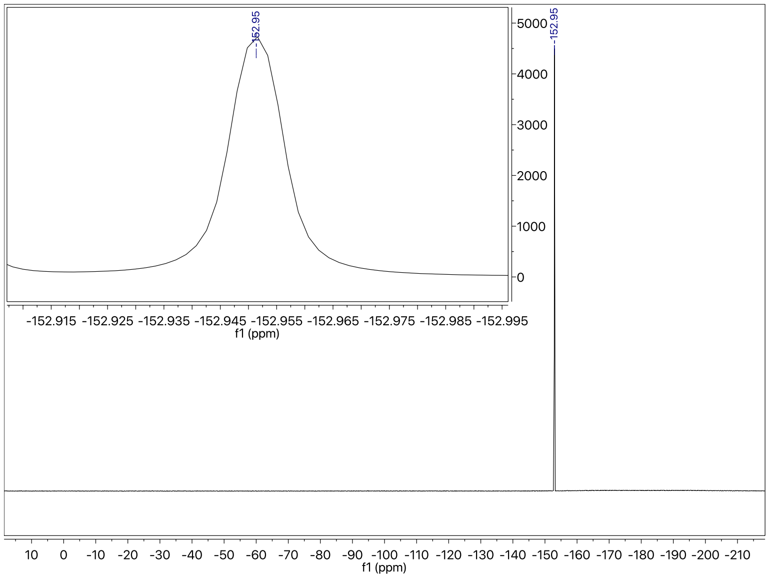

Figure S39. $^{19}F\{^{1}H\}$-NMR (400 MHz, $CDCl_3$, 298 K) spectrum of **C4**.

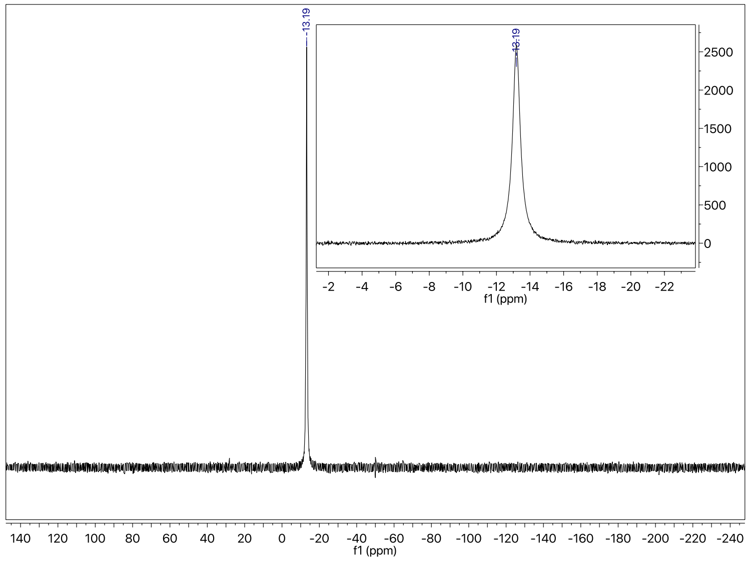

Figure S40. $^{31}P\{^{1}H\}$-NMR (160 MHz, $CDCl_3$, 298 K) spectrum of **C4**.

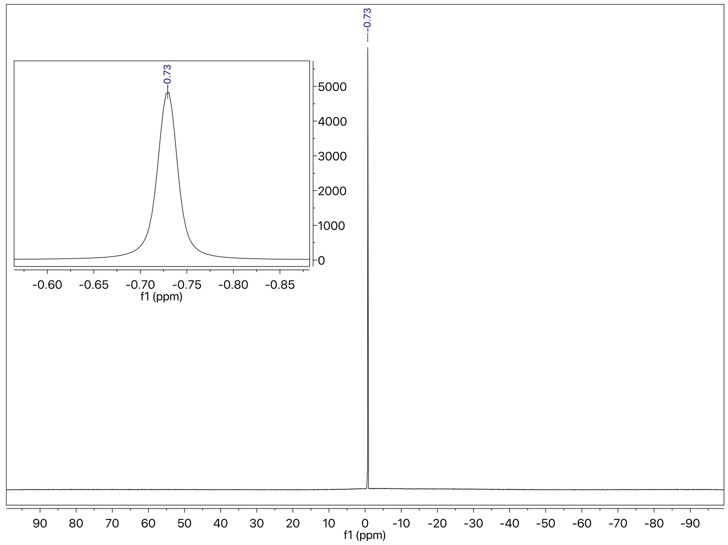

Figure S41. $^{11}B\{^{1}H\}$-NMR (128 MHz, $CDCl_3$, 298 K) spectrum of **C4**.

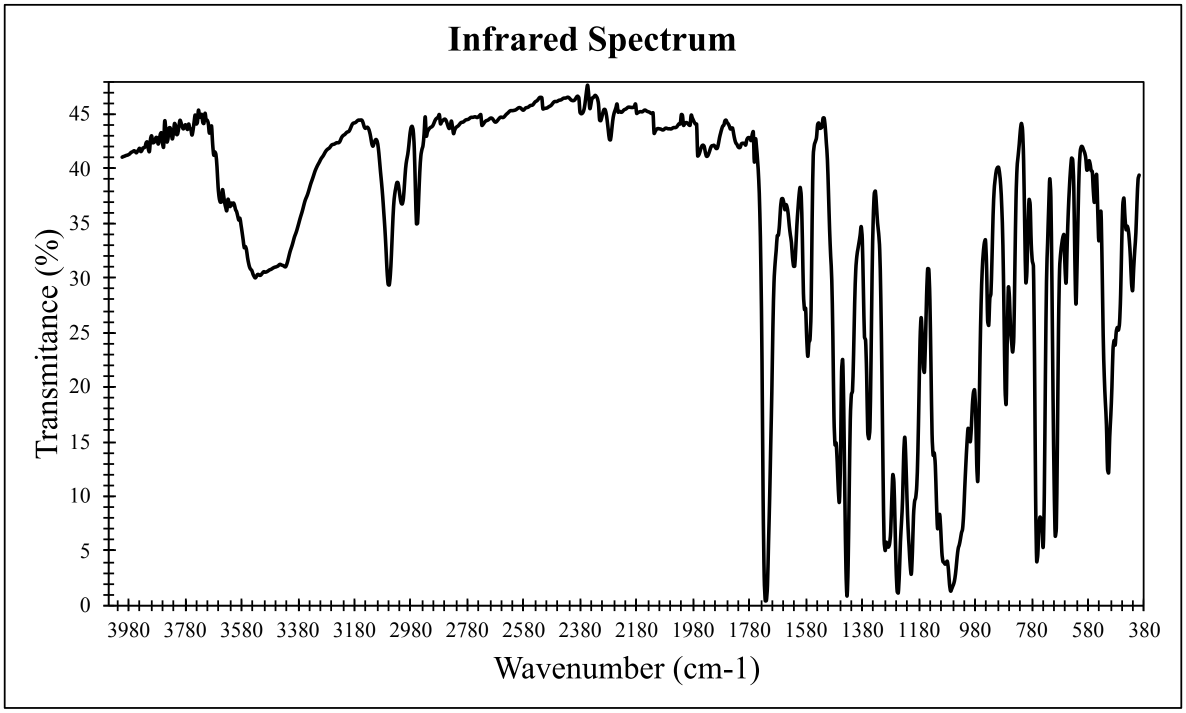

Figure S42. Infrared spectrum of **C4**.

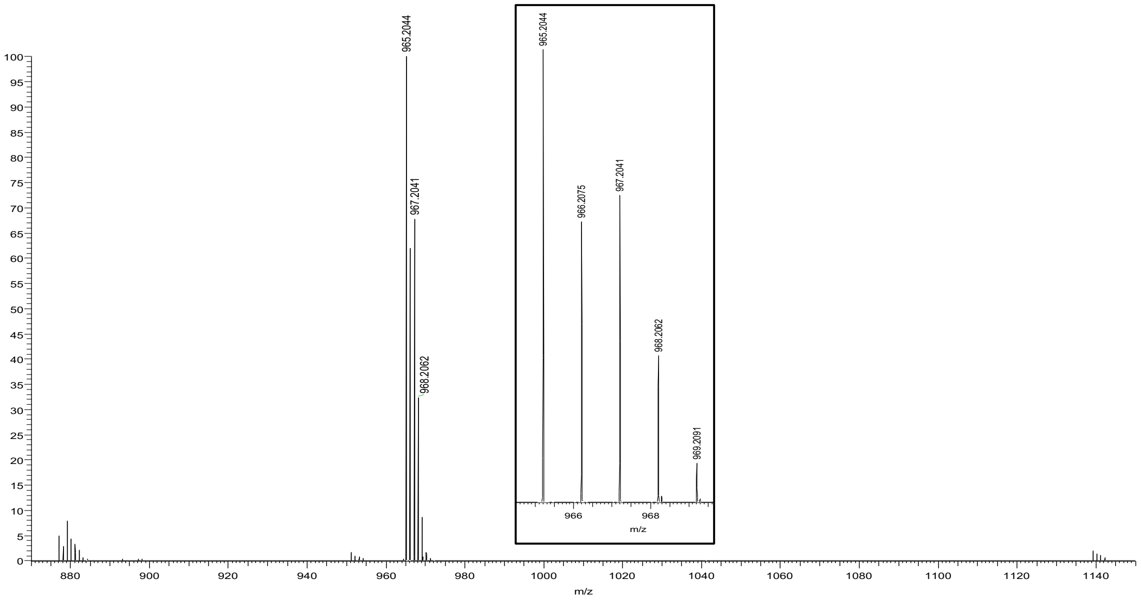

Figure S43. HRMS analysis of **C4** (positive ion mode).

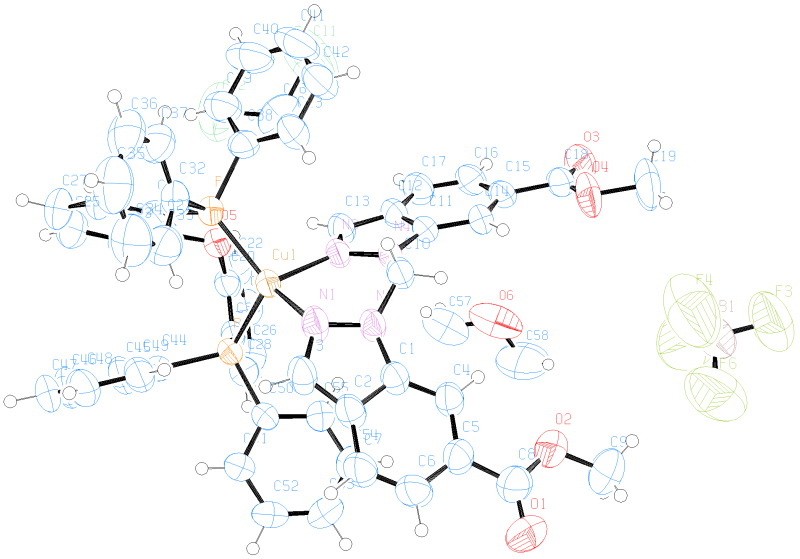

Figure S44. XRD molecular structure of **C4**.

Table S7. Selected crystallographic data of compound **C4**.

Bond distances

Atom 1	Atom 2	Length (A)
Cu1	P1	2.244(1)
Cu1	P2	2.292(2)
Cu1	N1	2.092(4)
Cu1	N3	2.094(3)
O1	C8	1.220(7)
O2	C8	1.337(7)
O3	C18	1.206(6)
O4	C18	1.325(7)
O5	C20	1.417(6)
O5	C21	1.406(5)
N1	N2	1.384(5)
N2	C10	1.434(6)
N3	N4	1.367(5)
N4	C10	1.444(5)

Selected Angles

Atom 1	Atom 2	Atom 3	Angle (º)
P1	Cu1	P2	113.49(4)
P1	Cu1	N1	124.8(1)
P1	Cu1	N3	107.1(1)
P2	Cu1	N1	105.7(1)
P2	Cu1	N3	111.0(1)
N1	Cu1	N3	92.6(1)
C20	O5	C21	118.6(3)
O1	C8	O2	123.5(6)
N2	C10	N4	112.7(3)
O3	C18	O4	124.2(5)

Selected Torsions

Atom 1	Atom 2	Atom 3	Atom 4	Torsion (º)
P1	Cu1	N1	N2	-124.0(3)
P2	Cu1	N3	N4	-118.1(3)
N1	Cu1	N3	N4	-10.2(3)
C20	O5	C21	C23	-33.0(6)
N1	N2	C10	N4	-80.9(5)
C6	C5	C8	O1	-4.7(9)
C16	C15	C18	O3	-4.5(8)

Table S8. Crystal data and structure refinement for **C4**.

Identification code	Ch80
Empirical formula	C116 H100 B2 Cl4 Cu2 F8 N8 O12 P4
Formula weight	2364.41
Temperature	294(2) K
Wavelength	0.71073 Å
Crystal system	Triclinic
Space group	P -1
Unit cell dimensions	a = 11.755(4) Å α = 77.868(8)°
	b = 12.771(4) Å β = 79.146(7)°
	c = 20.802(7) Å γ = 68.168(7)°
Volume	2813.3(16) $Å^3$
Z	1
Density (calculated)	1.396 Mg/m^3
Absorption coefficient	0.607 mm^{-1}
F(000)	1216
Crystal size	0.26 x 0.18 x 0.13 mm^3
Theta range for data collection	1.009 to 28.459°.
Index ranges	-15<=h<=15, -17<=k<=17, -27<=l<=27
Reflections collected	82929
Independent reflections	14100 [R(int) = 0.0875]
Completeness to theta = 25.242°	100.0 %
Absorption correction	Semi-empirical from equivalents
Max. and min. transmission	1 and 0.824
Refinement method	Full-matrix least-squares on F^2
Data / restraints / parameters	14100 / 0 / 705
Goodness-of-fit on F^2	1.026
Final R indices [I>2sigma(I)]	R1 = 0.0706, wR2 = 0.1853
R indices (all data)	R1 = 0.1393, wR2 = 0.2267
Largest diff. peak and hole	0.858 and -0.588 e.$Å^{-3}$

2. UV-VISIBLE SPECTROSCOPY

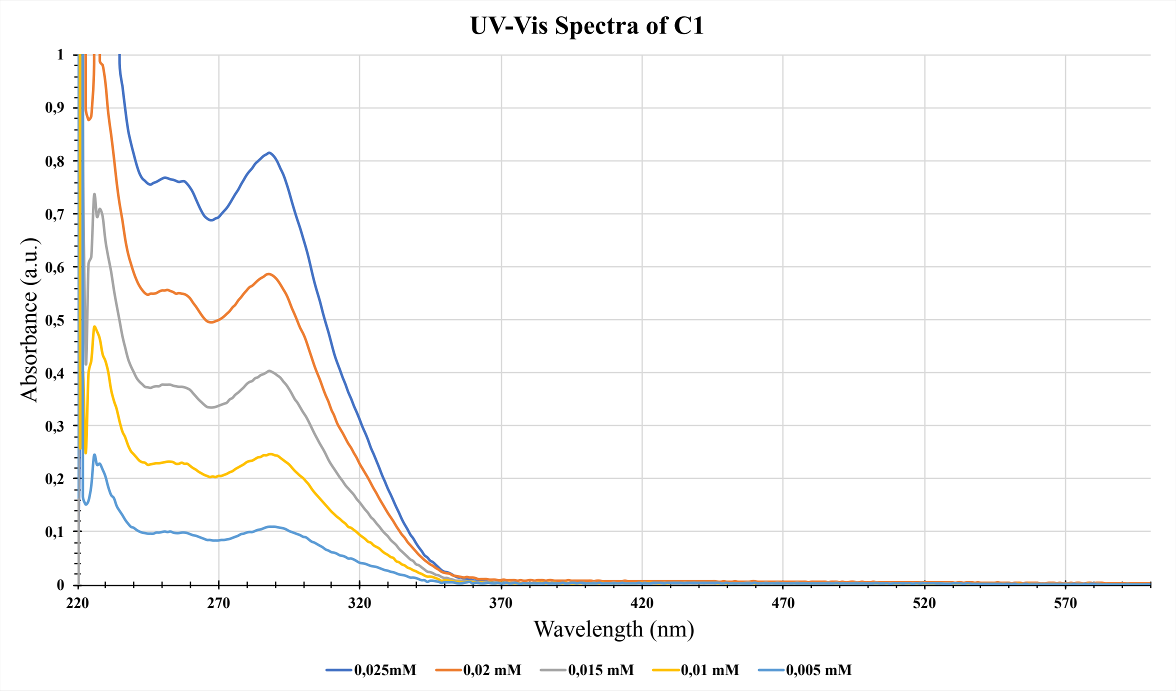

Figure S45. UV-Vis spectra of **C1** at different concentrations.

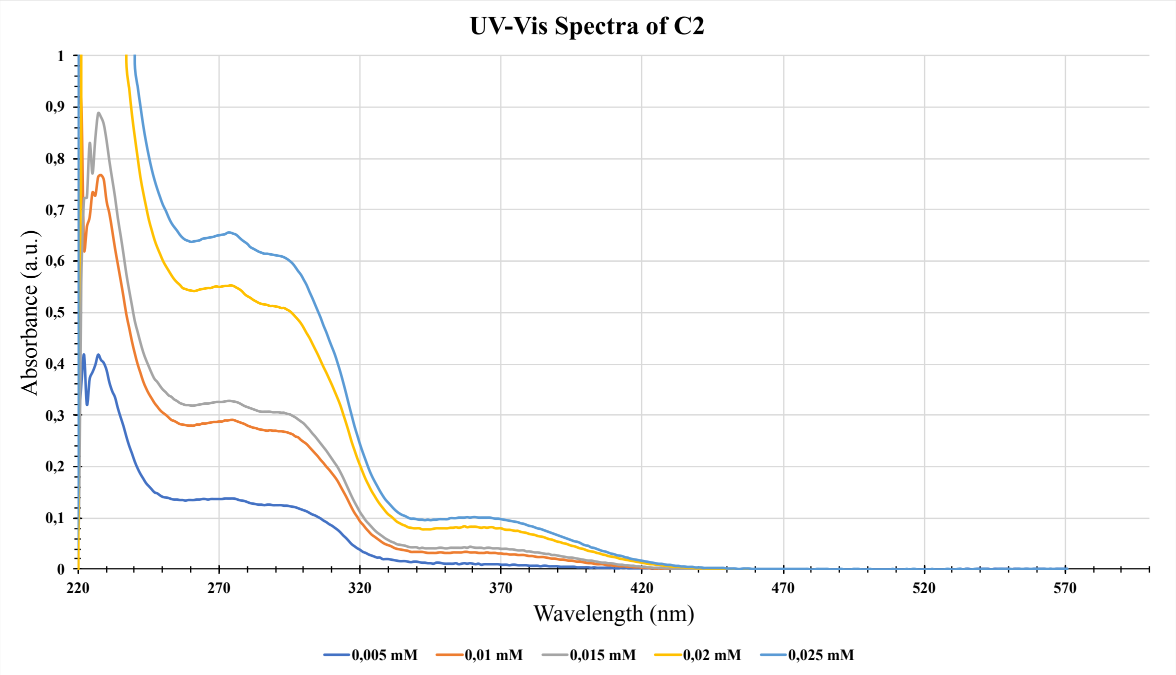

Figure S46. UV-Vis spectra of **C2** at different concentrations.

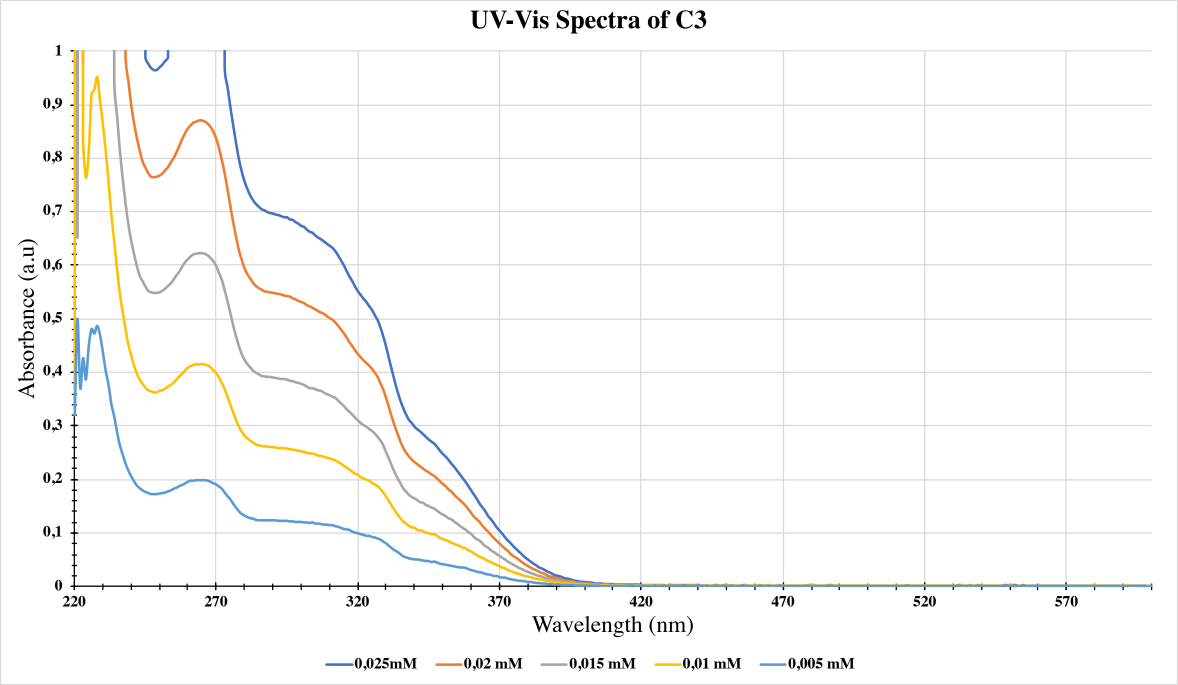

Figure S47. UV-Vis spectra of **C3** at different concentrations.

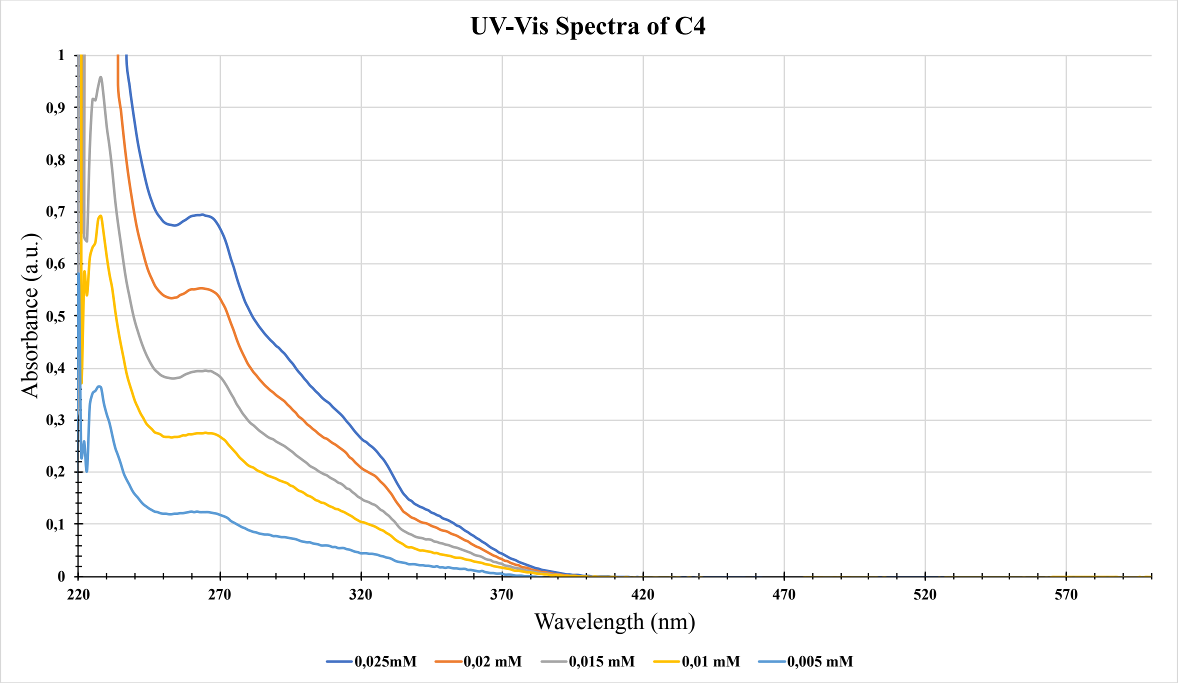

Figure S48. UV-Vis spectra of **C4** at different concentrations.

3. ELECTROCHEMICAL MEASUREMENTS

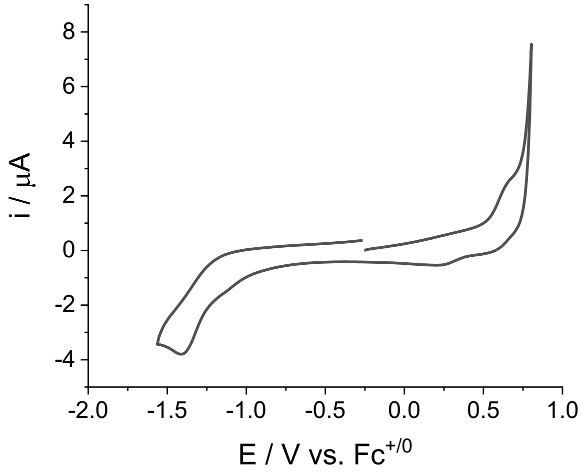

Figure S49. CV of complex **C1** in CH_2Cl_2 with 0.1 M $TBAPF_6$ as the supporting electrolyte and scan rate of 0.1 V/s.

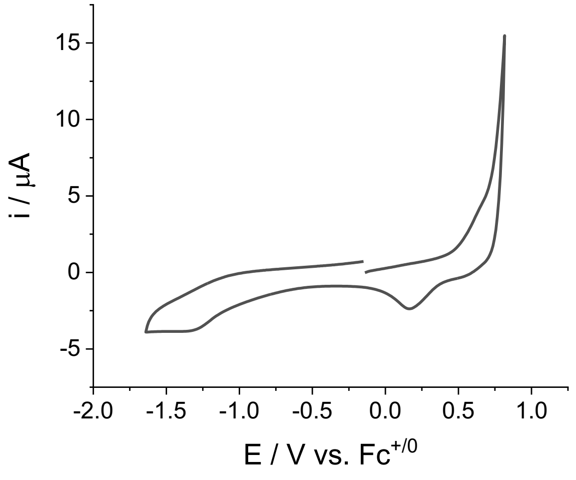

Figure S50. CV of complex **C2** in CH_2Cl_2 with 0.1 M $TBAPF_6$ as the supporting electrolyte and scan rate of 0.1 V/s (differentiation of the CV trace in the anodic scan was accomplished to extract the peak potential of the oxidation process reliably).

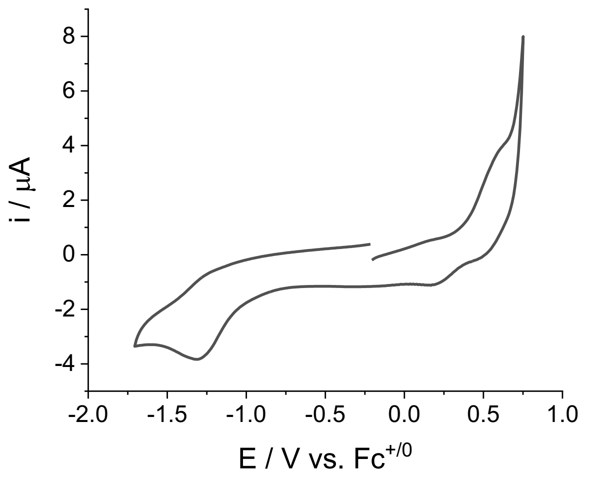

Figure S51. CV of complex **C3** in CH_2Cl_2 with 0.1 M $TBAPF_6$ as the supporting electrolyte and scan rate of 0.1 V/s.

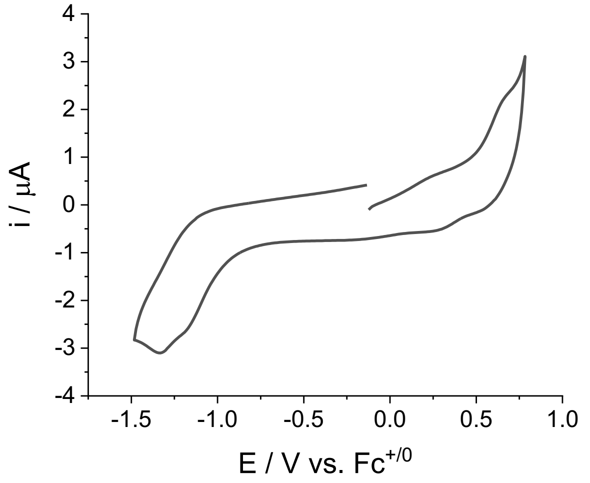

Figure S52. CV of complex **C4** in CH_2Cl_2 with 0.1 M $TBAPF_6$ as the supporting electrolyte and scan rate of 0.1 V/s.

4. EXCITED STATE LIFETIME MEASUREMENTS

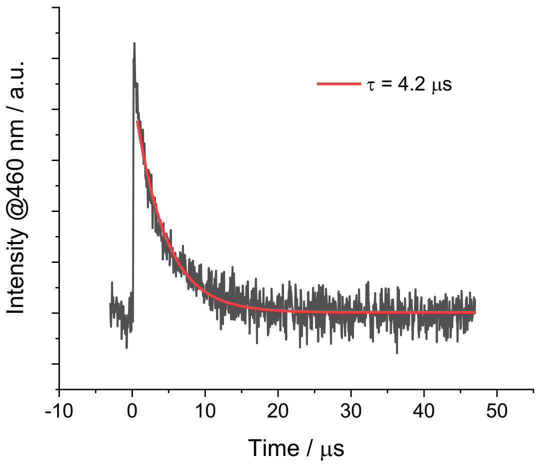

Figure S53. Time-resolved luminescence decay at 460 nm obtained by laser flash photolysis (excitation at 355 nm) of complex **C1** in N_2-purged CH_2Cl_2.

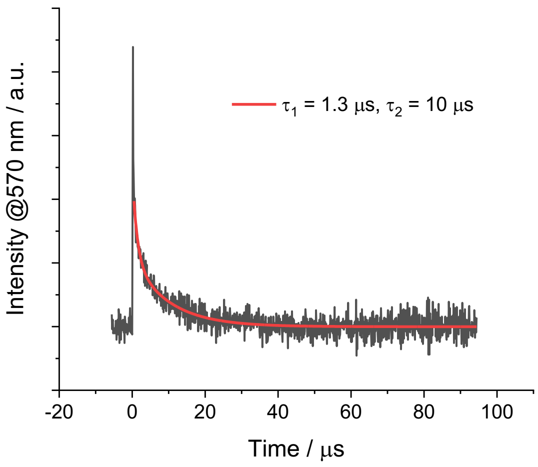

Figure S54. Time-resolved luminescence decay at 570 nm obtained by laser flash photolysis (excitation at 355 nm) of complex **C2** in N_2-purged CH_2Cl_2.

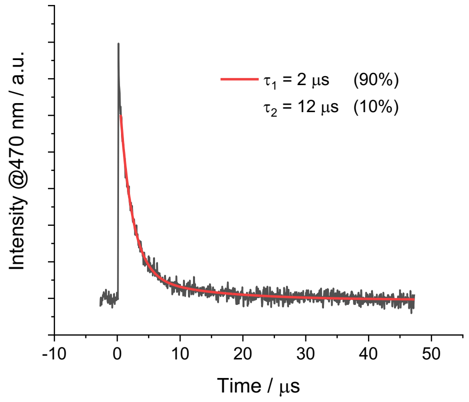

a)

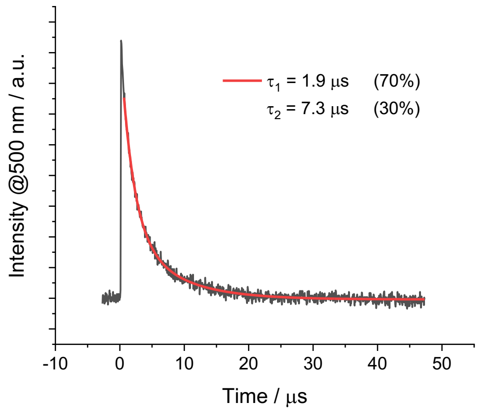

b)

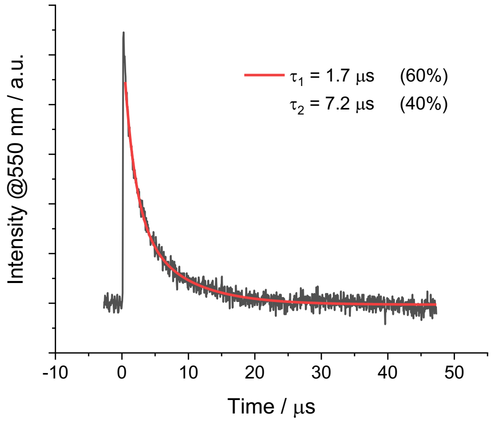

c)

Figure S55. Time-resolved luminescence decay at a) 470 nm, b) 500 nm, and c) 550 nm obtained by laser flash photolysis (excitation at 355 nm) of complex **C3** in N_2-purged CH_2Cl_2.

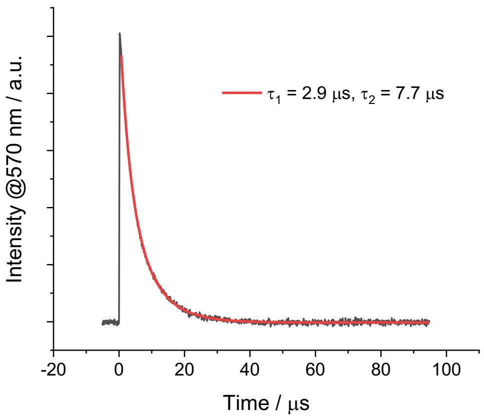

Figure S56. Time-resolved luminescence decay at 570 nm obtained by laser flash photolysis (excitation at 355 nm) of complex **C4** in N_2-purged CH_2Cl_2.

5. COMPUTATIONAL CALCULATIONS

5.1 Orbital Composition Analysis

Table S9: Orbital contributions using the natural atomic orbital basis over the metal centre (Cu), the *N,N*, and *P,P* ligand for selected orbitals in Table 2.

**Complex**	Orbital	*% Cu*	*% N,N*	*% P,P*
**C1**	HOMO	**29**	**9**	**63**
	LUMO	**1**	**95**	**4**
**C2**	HOMO-1	**39**	**12**	**49**
	HOMO	**24**	**4**	**72**
	LUMO	**2**	**95**	**3**
	LUMO+1	**1**	**97**	**2**
**C3**	HOMO	**27**	**7**	**66**
	LUMO	**1**	**96**	**3**
	LUMO+1	**1**	**96**	**3**
**C4**	HOMO	**26**	**7**	**67**
	LUMO	**1**	**97**	**2**
	LUMO+1	**1**	**99**	**1**

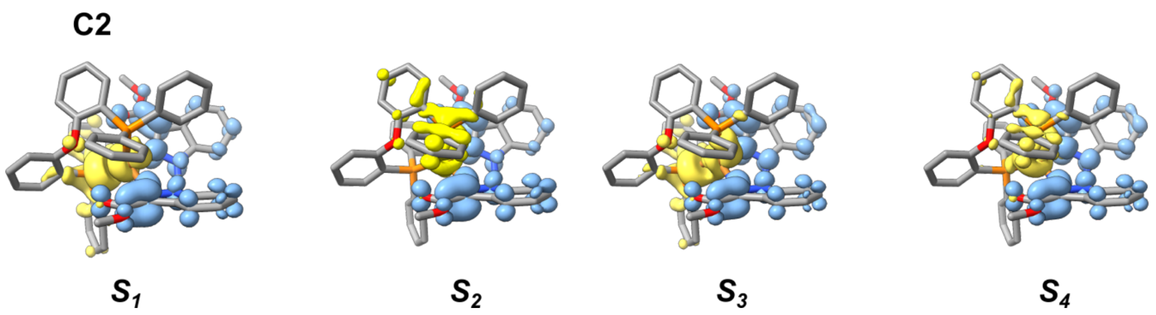

Figure S57: Hole-electron distributions (isovalue 0.0015 a.u.) for the first excited states of complex **C2**, in yellow and blue, respectively.

5.2 Cartesian Coordinates (atom X Y Z)

5.2.1 C1

Energy −3140.87016623 au

Cu	0.231978	0.106207	0.788661		H	−1.905742	1.752601	−5.349256
C	−4.750890	−0.705307	2.801790		H	−2.157907	0.004530	−3.619315
C	−3.416243	−0.985302	2.472729		C	4.647446	−1.966692	−1.713740
C	−2.981393	−2.293777	2.139414		C	4.099786	−1.557944	−2.929650
C	−3.901874	−3.358176	2.130570		C	3.157704	−0.529543	−2.970519
C	−5.218741	−3.085733	2.455168		C	2.760909	0.069928	−1.779569
C	−5.634364	−1.773215	2.785201		C	3.276159	−0.341250	−0.538859
C	−2.928406	2.780082	0.756407		C	4.235840	−1.360054	−0.526859
C	−2.233166	3.304337	−0.367015		H	5.382361	−2.764155	−1.685965
C	−2.911242	4.114285	−1.298642		H	4.402706	−2.037823	−3.854445
C	−4.253087	4.372261	−1.082629		H	2.726879	−0.204430	−3.910332
C	−4.929183	3.843118	0.044539		H	4.643064	−1.695002	0.420306
C	−4.288550	3.045627	0.980268		O	1.849478	1.112256	−1.740453
C	−0.914076	2.780214	−0.258985		P	−0.435549	−0.793271	−1.300121
N	−0.805870	2.005916	0.811452		P	2.499318	0.336977	0.973480
N	−2.018018	2.018713	1.445762		C	3.343591	−0.546401	2.332327
N	−2.306838	−0.180874	2.394130		C	2.650621	−1.595153	2.954548
N	−1.203430	−0.898461	2.015741		C	4.644267	−0.232826	2.752669
C	−1.593273	−2.156583	1.861234		C	3.256520	−2.333895	3.971428
C	−2.171455	1.233544	2.648453		H	1.634325	−1.822364	2.645231
H	−3.051889	1.578985	3.186975		C	5.245123	−0.968442	3.773702
H	−1.288114	1.391729	3.269234		H	5.182431	0.584629	2.283911
H	−0.067017	2.918020	−0.912993		C	4.554231	−2.020987	4.380681
H	−2.392263	4.509672	−2.165298		H	2.714388	−3.144963	4.447611
H	−4.805013	4.986391	−1.786056		H	6.251650	−0.721056	4.096386
H	−3.582841	−4.361049	1.868136		H	5.025029	−2.591096	5.175492
H	−5.951882	−3.885300	2.453140		C	3.102901	2.059163	1.069624
H	−6.676982	−1.597300	3.029724		C	2.343891	2.984440	1.800285
H	−5.086269	0.294421	3.052384		C	4.282622	2.476242	0.438329
H	−5.982951	4.065663	0.177179		C	2.770566	4.307559	1.912559
H	−4.822739	2.644046	1.833439		H	1.413392	2.668798	2.261198
H	−0.886168	−2.906644	1.537709		C	4.703543	3.801916	0.548033
C	1.028561	2.212426	−3.690093		H	4.864256	1.767222	−0.142154
C	0.020233	2.407602	−4.636161		C	3.950144	4.717500	1.286479
C	−1.120487	1.604127	−4.615439		H	2.178458	5.019784	2.479016
C	−1.259184	0.610486	−3.645187		H	5.617633	4.119890	0.056123
C	−0.263172	0.400045	−2.681787		H	4.278209	5.749098	1.368297
C	0.881124	1.216027	−2.731198		C	0.419182	−2.297300	−1.887613
H	1.923447	2.824900	−3.678442		C	0.329944	−2.732765	−3.217880
H	0.129045	3.185733	−5.384394		C	1.182967	−3.029310	−0.970263

C	0.997300	−3.887526	−3.620512		C	−2.665377	−2.546499	−1.459074
H	−0.252806	−2.164263	−3.935760		C	−4.480165	−0.523279	−0.805117
C	1.849516	−4.185819	−1.375362		H	−2.794615	0.799633	−0.863036
H	1.274069	−2.679053	0.053038		C	−4.018740	−2.844642	−1.286310
C	1.757228	−4.614838	−2.699690		C	−4.926008	−1.839118	−0.953676
H	0.928838	−4.219002	−4.651983		H	−5.175462	0.268420	−0.544411
H	2.449475	−4.741024	−0.661459		H	−4.360626	−3.868393	−1.402340
H	2.281519	−5.510666	−3.017606		H	−5.972926	−2.079509	−0.800829
C	−2.211060	−1.231460	−1.301839		H	−1.965908	−3.336995	−1.706890
C	−3.132065	−0.222891	−0.975955					

5.2.2 C2

Energy −3596.65472154 au

Cu	−0.229028	−0.017460	−0.428427		H	2.004479	5.468266	0.811672
C	3.573755	3.610259	−2.304477		H	4.081279	6.312279	−0.276912
C	2.411303	3.141860	−1.674710		C	−3.976989	−2.640551	1.880180
C	1.834049	3.801009	−0.559238		C	−5.216689	−3.001720	1.355564
C	2.437577	4.962910	−0.042450		C	−5.648884	−2.456297	0.144216
C	3.590096	5.422117	−0.654948		C	−4.838001	−1.554352	−0.541556
C	4.148616	4.753443	−1.771745		C	−3.590178	−1.167952	−0.028755
C	3.225651	−0.947731	−2.374351		C	−3.182730	−1.723922	1.192721
C	2.921040	−2.244232	−1.884646		H	−3.614838	−3.060740	2.812219
C	3.955067	−3.180189	−1.694220		H	−5.838842	−3.713267	1.888566
C	5.248765	−2.787060	−1.990199		H	−6.611261	−2.739358	−0.269271
C	5.532263	−1.488095	−2.477308		H	−5.168905	−1.147388	−1.490920
C	4.534042	−0.547954	−2.680528		C	−1.160523	1.242964	4.774240
C	1.506225	−2.227325	−1.673760		C	−2.476262	0.837953	4.551229
N	0.997665	−1.039782	−2.000139		C	−2.777527	−0.054333	3.520548
N	2.023645	−0.275641	−2.443921		C	−1.743427	−0.533083	2.721268
N	1.619628	2.039713	−1.916342		C	−0.408892	−0.133713	2.917335
N	0.605461	1.949016	−1.024389		C	−0.137631	0.764982	3.952934
C	0.710212	2.993517	−0.205169		H	−0.928901	1.935304	5.577015
C	1.720420	1.039260	−2.959400		H	−3.278340	1.214165	5.178298
H	0.766357	0.976702	−3.483462		H	−3.797680	−0.371789	3.342709
H	2.499219	1.342490	−3.655920		H	0.874141	1.120343	4.103423
C	0.582539	−3.270759	−1.182195		O	−1.925306	−1.419187	1.680985
O	−0.594094	−3.077911	−0.930268		P	−2.493898	−0.000456	−0.928477
O	1.198760	−4.453283	−1.073391		P	0.789859	−0.622249	1.616906
C	−0.248623	3.114480	0.909908		C	2.368359	0.214362	2.028760
O	−0.982118	2.215262	1.269083		C	3.017853	0.934384	1.019187
O	−0.202621	4.328176	1.475963		C	2.994094	0.083006	3.279998
C	0.383927	−5.538577	−0.582282		C	4.244731	1.555796	1.258321
H	−0.411948	−5.760082	−1.296203		H	2.569783	0.988188	0.038194
H	1.060325	−6.385063	−0.483470		C	4.216557	0.705993	3.522345

H	−0.043787	−5.274476	0.384565		H	2.531454	−0.516288	4.057373
C	−1.128007	4.525091	2.568655		C	4.839580	1.450899	2.515024
H	−0.919538	5.523733	2.947652		H	4.726189	2.117656	0.463959
H	−2.152598	4.454271	2.197972		H	4.688860	0.605335	4.494543
H	−0.966757	3.769430	3.339192		H	5.791558	1.934982	2.709402
H	6.560924	−1.219728	−2.693998		C	1.244998	−2.364756	2.001644
H	4.766037	0.444198	−3.049082		C	0.407356	−3.227765	2.720903
H	3.730332	−4.170264	−1.318718		C	2.480669	−2.843417	1.539435
H	6.067558	−3.483968	−1.846761		C	0.803379	−4.541553	2.975959
H	4.011061	3.108385	−3.159804		H	−0.547223	−2.876961	3.091231
H	5.052997	5.147478	−2.223343		C	2.869675	−4.158223	1.789304
H	3.148412	−2.186861	0.992500		H	−5.314153	3.308290	1.519921
C	2.033183	−5.011776	2.510436		H	−4.389354	5.252287	0.277519
H	0.149212	−5.196767	3.543165		C	−2.910197	−0.318042	−2.685712
H	3.828846	−4.511408	1.423584		C	−2.188497	−1.322306	−3.350415
H	2.337200	−6.034525	2.710011		C	−3.903311	0.390003	−3.377414
C	−3.227366	1.643574	−0.592401		C	−2.464483	−1.615752	−4.685608
C	−2.713118	2.745246	−1.295622		H	−1.421825	−1.869499	−2.814029
C	−4.169621	1.857085	0.418185		C	−4.170164	0.097536	−4.715978
C	−3.131562	4.036370	−0.986917		H	−4.462797	1.170502	−2.873207
H	−1.971238	2.590869	−2.072944		C	−3.452411	−0.904756	−5.371667
C	−4.584756	3.153797	0.730587		H	−1.904737	−2.396314	−5.192317
H	−4.574012	1.015892	0.969781		H	−4.939923	0.651511	−5.244623
C	−4.066057	4.245072	0.033421		H	−3.661335	−1.130032	−6.412990
H	−2.726042	4.880882	−1.535774					

5.2.3 C3

Energy −3596.65751482 au

Cu	−0.931150	0.666825	0.775978		O	6.090974	4.679535	−0.797479
C	4.222800	1.511013	2.348304		O	7.397021	3.528443	0.640783
C	2.936386	1.789491	1.857357		C	−1.930724	−3.670060	−1.634401
C	2.713209	2.756834	0.842767		C	−0.951858	−4.504240	−2.178316
C	3.794798	3.446671	0.282159		C	0.296448	−3.983137	−2.520859
C	5.071031	3.165041	0.752076		C	0.570405	−2.630355	−2.316667
C	5.274132	2.207202	1.781433		C	−0.393458	−1.775415	−1.763792
C	1.512151	−2.037388	2.215428		C	−1.646281	−2.322903	−1.438422
C	0.540991	−2.855136	1.576251		H	−2.907374	−4.047997	−1.352429
C	0.931505	−4.038780	0.937896		H	−1.163027	−5.558152	−2.324019
C	2.278600	−4.378498	0.949584		H	1.063592	−4.633229	−2.926381
C	3.231246	−3.567845	1.625592		H	1.549454	−2.237430	−2.564932
C	2.868508	−2.398877	2.270601		C	−5.035419	1.103727	−2.912456
C	−0.679914	−2.125831	1.680652		C	−4.514890	−0.011376	−3.569050
N	−0.482385	−0.966994	2.290662		C	−3.693957	−0.912391	−2.890523
N	0.842990	−0.924607	2.651799		C	−3.388332	−0.672743	−1.554908

N	1.708402	1.271068	2.168959		C	−3.876767	0.456840	−0.877343
N	0.720921	1.851017	1.412068		C	−4.716390	1.333609	−1.574519
C	1.308337	2.735565	0.619736		H	−5.676432	1.800294	−3.442124
C	1.354854	0.308971	3.193183		H	−4.744885	−0.186766	−4.614812
H	2.243030	0.104415	3.788120		H	−3.285403	−1.781561	−3.392308
H	0.580792	0.732611	3.833755		H	−5.100926	2.213521	−1.071849
H	−1.665506	−2.376967	1.319834		O	−2.598951	−1.530642	−0.806806
H	0.221861	−4.661910	0.408823		P	−0.079193	−0.015419	−1.350424
H	3.660492	4.172367	−0.510710		P	−3.226027	0.806611	0.796742
H	6.284623	2.017154	2.120818		C	−3.974905	2.412725	1.236632
H	4.398656	0.781564	3.130691		C	−3.170471	3.558011	1.153216
H	4.270362	−3.871996	1.616519		C	−5.315016	2.535281	1.631856
H	3.610397	−1.776319	2.756017		C	−3.703079	4.814540	1.443476
H	0.723488	3.300664	−0.091300		H	−2.126441	3.459520	0.868437
C	6.208446	3.875538	0.111875		C	−5.843166	3.791319	1.927748
C	8.547037	4.173183	0.063647		H	−5.938866	1.650329	1.707592
H	8.478239	5.256111	0.186612		C	−5.039567	4.930941	1.830498
H	9.405700	3.779952	0.605434		H	−3.075253	5.697476	1.375920
H	8.622453	3.936672	−0.999870		H	−6.880464	3.881901	2.234512
C	2.682518	−5.593956	0.193714		H	−5.453914	5.907073	2.062467
C	4.473527	−6.979246	−0.462845		C	−4.056828	−0.406938	1.882765
H	4.246437	−6.876997	−1.526182		C	−3.571574	−0.562038	3.189880
H	5.549861	−7.009788	−0.301785		C	−5.144749	−1.178020	1.453062
H	4.001638	−7.886644	−0.080183		C	−4.175810	−1.469542	4.058474
O	1.910128	−6.299393	−0.432876		H	−2.711421	0.013254	3.515037
O	4.006334	−5.826866	0.262141		C	−5.741564	−2.091683	2.323077
H	−5.522695	−1.064984	0.442450		H	−2.494143	3.763825	−3.424516
C	−5.259751	−2.237601	3.625394		H	−2.234549	2.923228	−5.747679
H	−3.795363	−1.584512	5.068743		C	1.729933	0.186954	−1.540663
H	−6.583028	−2.687618	1.983389		C	2.577697	−0.572579	−0.720061
H	−5.724793	−2.949759	4.299961		C	2.284789	1.171098	−2.369546
C	−0.773109	0.898591	−2.774879		C	3.952925	−0.356221	−0.728486
C	−0.631681	0.425533	−4.087738		H	2.163438	−1.340161	−0.079780
C	−1.442587	2.106041	−2.544421		C	3.663887	1.389147	−2.372113
C	−1.154624	1.154537	−5.153091		C	4.499440	0.630913	−1.551917
H	−0.120883	−0.514150	−4.272102		H	4.591938	−0.951561	−0.084490
C	−1.965713	2.834773	−3.612836		H	4.083688	2.160902	−3.009636
H	−1.571673	2.465001	−1.528369		H	5.568858	0.814408	−1.545795
C	−1.822133	2.359796	−4.916524		H	1.644250	1.769589	−3.007247
H	−1.045796	0.782346	−6.167030					

5.2.4 C4

Energy −3596.65556396 au

Cu	−1.100112	−0.057745	0.705648		H	4.367890	−1.672405	1.538933

C	3.859187	1.414854	1.958959		H	−0.312741	−2.733268	−1.156241
C	2.473088	1.573763	1.912197		C	−2.100705	−2.337117	−3.610110
C	1.858284	2.807189	1.578570		C	−1.207841	−2.383616	−4.682811
C	2.659889	3.937860	1.328480		C	−0.217908	−1.408343	−4.809145
C	4.032670	3.790919	1.378262		C	−0.113527	−0.389813	−3.860614
C	4.627300	2.535474	1.664600		C	−0.995977	−0.324901	−2.773680
C	2.500515	−2.128128	0.488324		C	−1.991085	−1.313878	−2.674383
C	1.895837	−2.738206	−0.643303		H	−2.876006	−3.084604	−3.482853
C	2.703583	−3.363401	−1.613735		H	−1.287855	−3.181270	−5.413873
C	4.074441	−3.346445	−1.437750		H	0.477368	−1.441296	−5.641387
C	4.659682	−2.730151	−0.298883		H	0.671244	0.352788	−3.951639
C	3.884099	−2.126015	0.683741		C	−6.088846	1.195650	−1.154097
C	0.504295	−2.466194	−0.503744		C	−5.637676	0.872709	−2.433891
N	0.278376	−1.749319	0.589791		C	−4.543548	0.023720	−2.605492
N	1.478351	−1.572097	1.215796		C	−3.900779	−0.482306	−1.480708
N	1.456687	0.670156	2.090989		C	−4.317579	−0.151083	−0.180637
N	0.240972	1.240334	1.830794		C	−5.429861	0.685604	−0.035180
C	0.464667	2.516776	1.541986		H	−6.941973	1.853184	−1.025277
C	1.528244	−0.737521	2.394321		H	−6.135000	1.280763	−3.307628
H	2.452850	−0.940317	2.932190		H	−4.185957	−0.232682	−3.595912
H	0.679508	−0.992573	3.029742		H	−5.764345	0.956333	0.959910
C	6.138497	−2.677328	−0.103977		O	−2.823913	−1.349393	−1.563687
O	6.677106	−2.178419	0.870050		P	−0.837888	0.910707	−1.427511
O	6.812650	−3.232092	−1.122113		P	−3.247126	−0.669379	1.209953
C	6.111134	2.424033	1.571285		C	−4.033803	0.084732	2.675993
O	6.864338	3.369202	1.418065		C	−3.461849	1.259663	3.186189
O	6.518425	1.144672	1.637023		C	−5.180319	−0.446161	3.284379
C	8.248779	−3.206670	−1.007758		C	−4.039270	1.906663	4.279062
H	8.566903	−3.735911	−0.107421		H	−2.560025	1.657703	2.729973
H	8.621157	−3.706493	−1.900101		C	−5.751316	0.198976	4.381111
H	8.606275	−2.175620	−0.966440		H	−5.621904	−1.361126	2.903236
C	7.932541	0.909542	1.504313		C	−5.184564	1.376530	4.876512
H	8.463043	1.289805	2.380622		H	−3.591750	2.816180	4.667600
H	8.032582	−0.170929	1.422835		H	−6.637845	−0.216774	4.849719
H	8.314902	1.405862	0.610019		H	−5.631564	1.875483	5.730685
H	−0.360356	3.167060	1.290094		C	−3.533387	−2.466234	1.371197
H	2.209082	4.890695	1.075192		C	−2.528266	−3.244138	1.963048
H	4.689019	4.625839	1.164443		C	−4.710268	−3.080500	0.921742
H	4.333263	0.468174	2.174527		C	−2.706965	−4.618341	2.120755
H	2.258196	−3.827971	−2.486522		H	−1.603419	−2.772956	2.280088
H	4.724522	−3.801483	−2.173894		C	−4.883431	−4.456293	1.075937
H	−5.483512	−2.484814	0.447000		H	−4.263812	4.304993	−0.393355
C	−3.884344	−5.225409	1.677300		H	−4.555325	5.035487	−2.749874
H	−1.925055	−5.215719	2.579535		C	0.821780	1.636106	−1.687463

H	−5.796486	−4.927430	0.725321		C	1.934519	0.822654	−1.419833
H	−4.020341	−6.296111	1.793896		C	1.023977	2.980160	−2.023858
C	−2.013775	2.230644	−1.886750		C	3.224833	1.339858	−1.487900
C	−2.186370	2.640793	−3.216978		H	1.791049	−0.218882	−1.164174
C	−2.765173	2.838132	−0.873132		C	2.319589	3.497051	−2.090445
C	−3.096578	3.649920	−3.523921		H	0.175346	3.622923	−2.228667
H	−1.616807	2.164498	−4.008607		C	3.419399	2.682898	−1.819669
C	−3.676039	3.847993	−1.182942		H	4.072627	0.699736	−1.265432
H	−2.655895	2.501595	0.152793		H	2.466882	4.541752	−2.345971
C	−3.841548	4.254441	−2.507296		H	4.422795	3.094424	−1.853744
H	−3.229024	3.962078	−4.555141					
